# Revisiting the Synthesis of Functionally Substituted 1,4-Dihydrobenzo[*e*][1,2,4]triazines

**DOI:** 10.3390/molecules27082575

**Published:** 2022-04-15

**Authors:** Margarita A. Epishina, Alexander S. Kulikov, Leonid L. Fershtat

**Affiliations:** Laboratory of Nitrogen Compounds, N.D. Zelinsky Institute of Organic Chemistry, Russian Academy of Sciences, Leninsky prosp., 47, 119991 Moscow, Russia; margarita-epishina@yandex.ru (M.A.E.); chimalex@yandex.ru (A.S.K.)

**Keywords:** 1,4-dihydrobenzo[*e*][1,2,4]triazine, Blatter radicals, oxidative cyclization, aerial oxidation, amidrazones, thermal stability

## Abstract

A series of novel 1,4-dihydrobenzo[1,2,4][*e*]triazines bearing an acetyl or ester moiety as a functional group at the C(3) atom of the 1,2,4-triazine ring were synthesized. The synthetic protocol is based on an oxidative cyclization of functionally substituted amidrazones in the presence of DBU and Pd/C. It was found that the developed approach is suitable for the preparation of 1,4-dihydrobenzo[*e*][1,2,4]triazines, but the corresponding Blatter radicals were isolated only in few cases. In addition, a previously unknown dihydrobenzo[*e*][1,2,4]triazolo[3,4-*c*][1,2,4]triazine tricyclic open-shell derivative was prepared. Studies of thermal behavior of the synthesized 1,4-dihydrobenzo[1,2,4][*e*]triazines revealed their high thermal stability (up to 240–250 °C), which enables their application potential as components of functional organic materials.

## 1. Introduction

A creation of novel functional organic materials remains one of the urgent goals in modern chemistry and materials science [1,2,3,4]. Such materials constitute a large variety of usually conjugated organic compounds with different chemical and physical properties. Recent achievements of numerous research groups worldwide confirmed that an incorporation of a nitrogen heteroaromatic motif usually enhances the quality of materials compared to their carbocyclic analogues [5,6,7].

Among nitrogen heterocyclic species, 1,4-dihydrobenzo[*e*][1,2,4]triazine motif is of special importance due to a wide range of applications inherent to compounds derived thereof. Such fused heterocyclic systems display various pharmacological activities, including antibacterial and antiproliferative activity [8,9,10]. Moreover, dihydrobenzo[*e*][1,2,4]triazines are direct precursors to the corresponding nitrogen-centered benzotriazinyl radicals, also known as Blatter radicals [11,12,13], which possess ferromagnetic and antiferromagnetic properties [14,15,16,17], and are valuable components of functional organic materials used in molecular grafting [18], preparation of liquid crystals [19,20], molecular electronics and spintronics [21,22], and for some other applications [23,24,25]. Therefore, synthetic strategies toward the construction of the 1,4-dihydrobenzo[*e*][1,2,4]triazine scaffold need to be constantly explored.

**Scheme 1:**
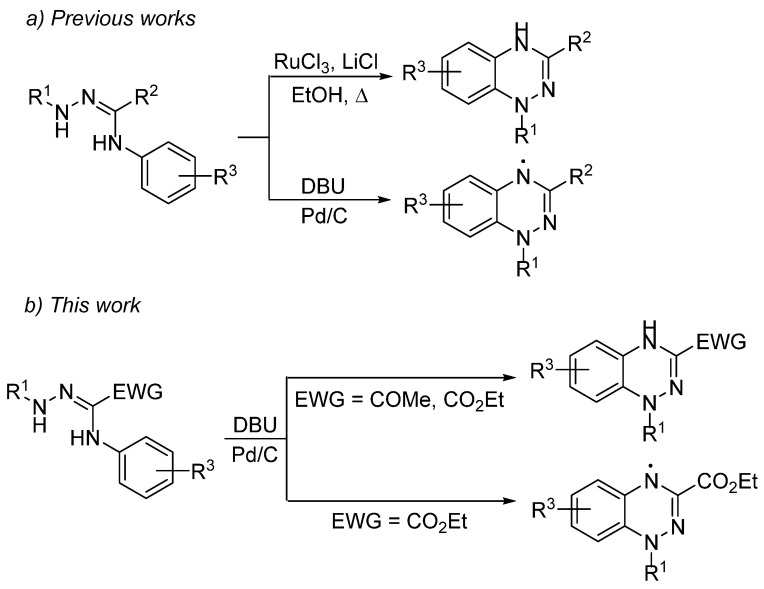
Methods for the construction of the 1,4-dihydrobenzo[*e*][1,2,4]triazine scaffold: (**a**) known methods and (**b**) method described in this manuscript.

There are a number of commonly used synthetic methods for the assembly of the 1,4-dihydrobenzo[*e*][1,2,4]triazine framework from various acyclic precursors [8,12]. The most widely explored protocol is based on an oxidative cyclization of (arylamino)hydrazones, also known as amidrazones. An interesting feature of this approach is its regiodiversity: RuCl_3_-mediated oxidation of amidrazones affords dihydrobenzo[*e*][1,2,4]triazines [26], while DBU-catalyzed aerial oxidation in the presence of Pd/C results in a direct formation of stable Blatter radicals (Scheme 1a) [12,27,28,29]. The latter protocol is generally used, and usually provides benzotriazinyl radicals in moderate to good yields, although only aromatic and heteroaromatic substituents are known to be installed onto the 1,2,4-triazine ring [11,12,27]. In this regard, a thorough investigation of scope and limitations of this approach for an assembly of the 1,4-dihydrobenzo[*e*][1,2,4]triazine core (either as Blatter radical or not) bearing additional functional groups remains relevant. It should be noted that electron-withdrawing groups at the fused benzo[1,2,4]triazine system may significantly affect both the reactivity and stability of Blatter radicals, and may be used for tuning functional properties of the resulted compounds. Herein, we disclose a divergent approach toward the construction of 1,4-dihydrobenzo[*e*][1,2,4]triazines bearing functional moieties at the triazine ring via DBU-catalyzed aerial oxidation of amidrazones in the presence of Pd/C (Scheme 1b).

## 2. Results and Discussion

**Scheme 2:**
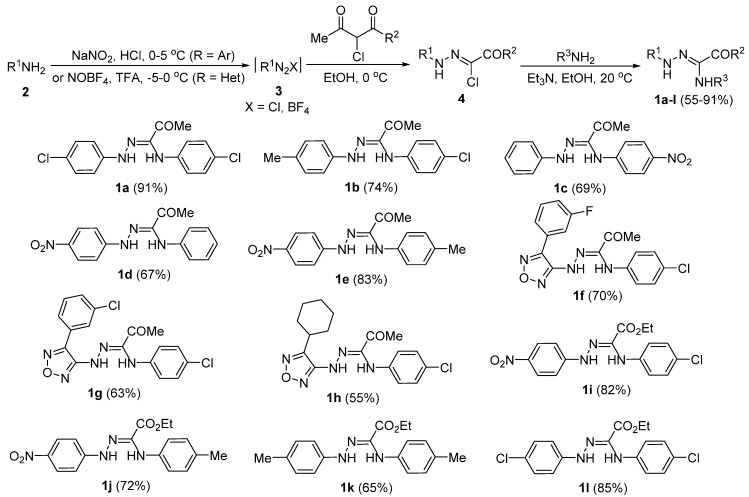
Synthesis of amidrazones **1a–l**.

Our investigations towards the desired approach for the formation of 1,4-dihydrobenzo[*e*][1,2,4]triazines began from the preparation of a wide set of amidrazones **1**. The synthesis of compound **1** is based on a simple three-step reaction sequence starting from readily available amine **2**. It includes diazotization of amine **2**, followed by the introduction of formed (het)arene diazonium salts **3** into the Japp–Klingemann reaction with chloroacetylacetone or chloroacetoacetic ester. This one-pot procedure enables an easy preparation of functionalized chlorohydrazone **4** bearing an acetyl or an ester moiety. Subsequent treatment of compound **4** with anilines afforded a series of amidrazones **1** (Scheme 2).

To optimize the reaction conditions for the desired synthesis of 1,4-dihydrobenzo[*e*][1,2,4]triazines, amidrazone **1a** was selected as a model substrate. Various bases, additives, solvents, temperatures and reaction times were varied (Table 1). It was found that conditions used in the fundamental work by Koutentis et al. [27] for the synthesis of Blatter radicals (a combination of catalytic amounts of DBU and 5% Pd/C) were inappropriate in the case of amidrazone **1a** (Table 1, entry 1), and neither benzotriazine **5a** nor Blatter radical **6a** were formed. The same result was observed upon using a stoichiometric amount of DBU in the absence of Pd/C (entry 2). An increase of the amount of Pd/C up to 15 mol.% catalyzed the oxidative cyclization of amidrazone **1a**, but only benzotriazine **5a** was formed as a sole product (entry 3). An increase of the reaction temperature decreased the reaction time, but also slightly decreased the yield of **5a** (entry 4). More promising results were obtained upon utilization of two equiv. of DBU: target product **5a** was obtained in a yield of 69% (entry 5). Further replacements of base, additive or solvent were less effective and provided benzotriazine **5a** in poor yields (entries 6–11). Therefore, the optimal conditions for the synthesis of benzotriazine **5a** were using two equiv. of DBU, 15 mol.% of 5% Pd/C in CH_2_Cl_2_ at 25 °C for 8 h (entry 5). Interestingly, in all optimization experiments, the formation of Blatter radical **6a** was not observed, despite in all cases chromatography being used to isolate reaction products. Arguably, aerial oxidation of benzotriazine **5a** does not proceed under these conditions, or acetyl-substituted Blatter radical **6a** is substantially destabilized by the electron-withdrawing effect of the acetyl moiety. Our attempts to oxidize compound **5a** to the Blatter radical **6a** using MnO_2_ or NaIO_4_ or upon prolonged refluxing in *o*-xylene were unsuccessful and returned the starting material without decomposition, confirming the resistance of the thus obtained 1,4-dihydrobenzo[*e*][1,2,4]triazine **5a** towards oxidation.

**Scheme 3:**
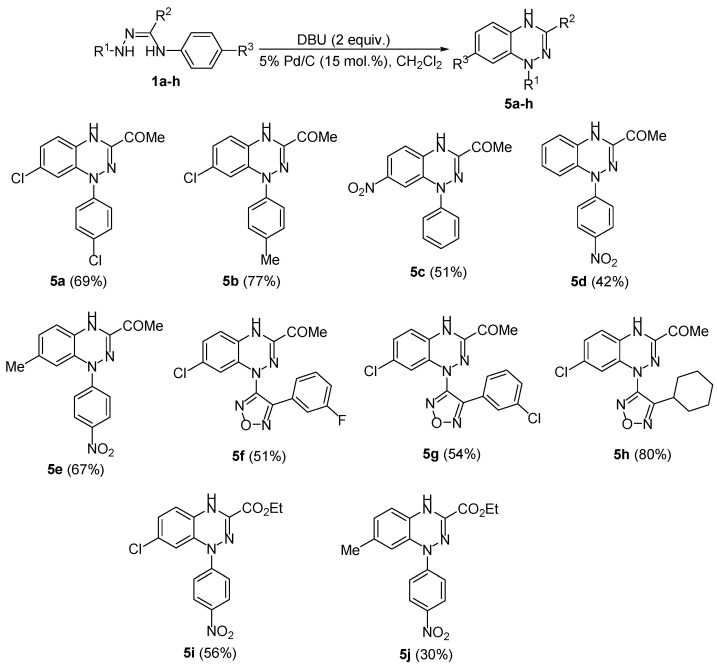
Synthesis of benzotriazines **5a–j** using the conditions indicated in the entry 5 of Table 1.

To further evaluate the scope of the observed transformation, other acetyl-substituted amidrazones **1b–h** were subjected to the optimized reaction conditions. Aside from dichloro derivative **5a**, benzotriazine **5b** bearing electron-donor *p*-tolyl substituent was formed in a good yield. Importantly, amidrazones **1c–e** incorporating electron-withdrawing *p*-nitrophenyl moiety either at the hydrazone or amine motifs also smoothly underwent cyclization to form corresponding heterocyclic products **5c–e** (Scheme 3). Similar results were obtained in the case of 1,2,5-oxadiazolyl substituted substrates **1f–h**, which confirmed the lack of influence of electronic effects of aromatic or heteroaromatic subunits on the cyclization outcome. In addition, 1,4-dihydrobenzo[*e*][1,2,4]triazines **5i,j**, bearing an ester functionality at the C(3) carbon atom of the heterocyclic moiety and electron-withdrawing *p*-nitrophenyl fragments at the N(1) nitrogen atom, were obtained in fair yields under the same conditions. All compounds were fully characterized by IR, ^1^H and ^13^C NMR spectroscopy (see Supplementary Materials), as well as high-resolution mass spectrometry and elemental analysis.

**Scheme 4:**
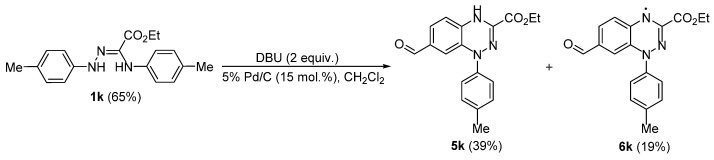
Oxidative cyclization of amidrazone **1k** using the conditions indicated in entry 5 of Table 1.

More intriguing results were obtained upon oxidative cyclization of amidrazones **1k** and **1l**. Introduction of substrate **1k** into the studied transformation provided two compounds **5k** and **6k** in a ratio 2:1, both of which possessed a formyl functionality as a result of oxidation of one of the methyl groups in the *p*-tolyl motif (Scheme 4). According to the ^1^H and ^13^C NMR spectroscopy data, the structure of the major product was assigned to as benzo[1,2,4]triazine **5k**. At the same time, the structure of compound **6k** was determined as a Blatter radical, which was confirmed by the presence of the multiplet signal in the EPR spectrum and HRMS data.

**Scheme 5:**
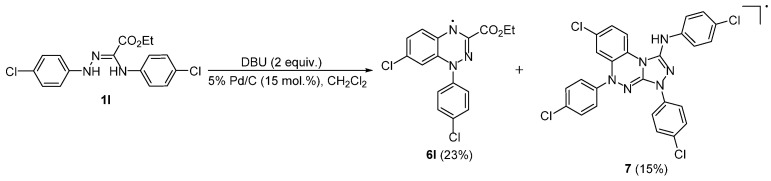
Oxidative cyclization of amidrazone **1l** using the conditions indicated in entry 5 of Table 1.

**Figure 1:**
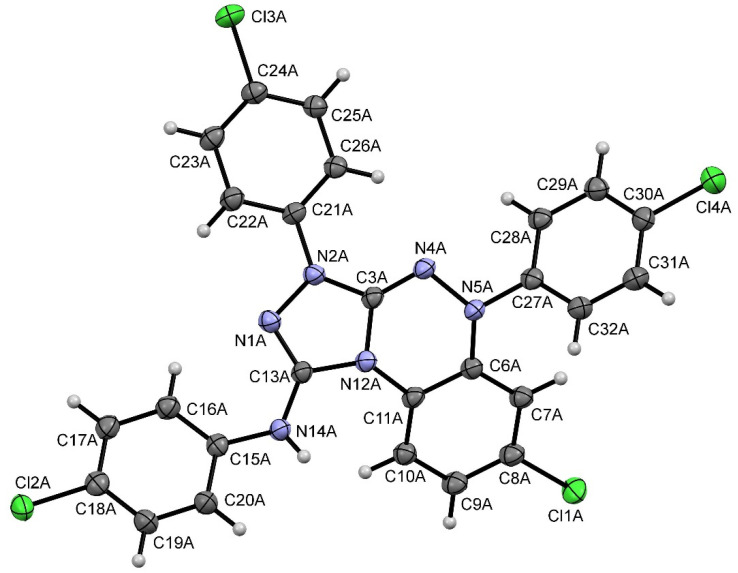
The crystal structure of compound **7**. All atoms are shown as probability ellipsoids of atomic displacements (*p* = 50%).

Oxidative cyclization of amidrazone **1l** also resulted in the formation of two compounds **6l** and **7** in moderate yields, although both of these derivatives were found to possess an unpaired electron (Scheme 5). Based on the EPR and HRMS data, the structure of **6l** was assigned as the corresponding Blatter radical. The formation of Blatter radicals from amidrazones **1k,l** is arguably attributed to the presence of electron-donating *p*-tolyl and *p*-chlorophenyl moieties at the N(1) atom of the benzo[1,2,4]triazine scaffold, while in the case of substrates **1i,j**, the electron-withdrawing effect of the *p*-nitrophenyl substituent suppresses the oxidation to Blatter radicals. The structure of compound **7** was unambiguously confirmed by X-ray diffraction study and was characterized as dihydrobenzo[*e*][1,2,4]triazolo [3,4-*c*][1,2,4]triazine derivative (Figure 1). According to the EPR spectrum, compound **7** also represents an organic radical, although the corresponding signal is broadened due to the delocalization of the unpaired electron in the tricyclic system.

**Scheme 6:**
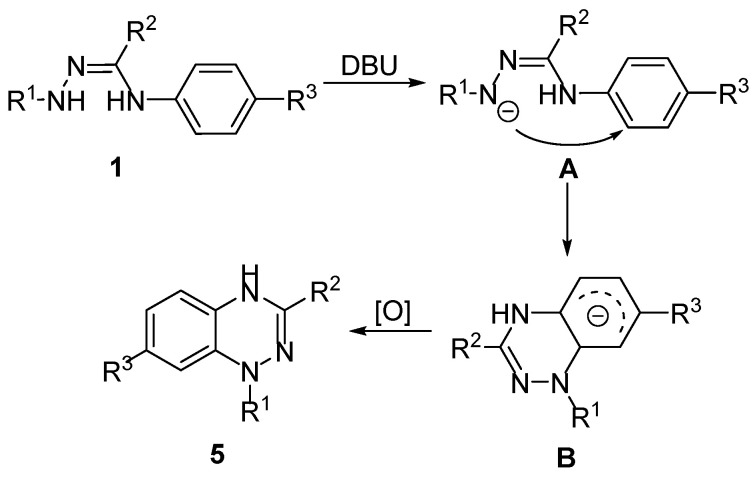
A plausible mechanism for the formation of benzo [1,2,4]triazines **5**.

A plausible mechanism for the formation of benzo[1,2,4]triazines **5** is depicted in Scheme 6. At the first step, DBU acts as a strong base to deprotonate the N-NH-fragment and the generated anion **A** undergoes intramolecular nucleophilic attack onto the benzene ring with the formation of anionic σ-complex **B**. Further oxidation of intermediate **A** furnishes the formation of benzo[1,2,4]triazine **5**.

**Scheme 7:**
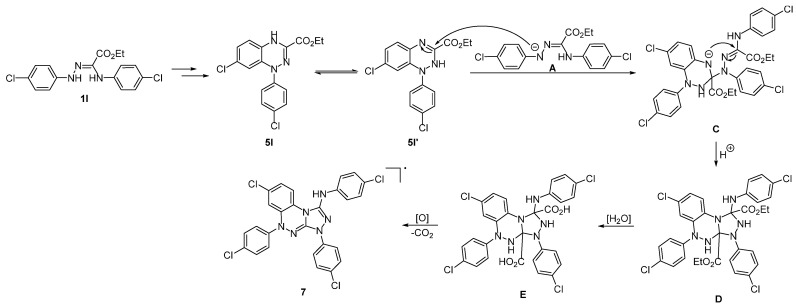
A plausible mechanism for the formation of compound **7**.

For the formation of the previously unknown compound **7**, the following mechanism was proposed (Scheme 7). Introduction of amidrazone **1l** into oxidative cyclization under standard reaction conditions affords benzo[1,2,4]triazine **5l**, which tautomerizes to the N(2)H form **5l’** with subsequent nucleophilic addition of the amidrazone anion **A**. Intermediate **C** undergoes cyclization to the perhydro[1,2,4]triazolo[3,4-*c*][1,2,4]triazine derivative **D**. Hydrolysis of both ester moieties in D under traces of moisture affords intermediate **E**, which undergoes oxidative decarboxylation to the final dihydrobenzo[*e*][1,2,4]triazolo[3,4-*c*][1,2,4]triazine derivative **7**.

**Scheme 8:**
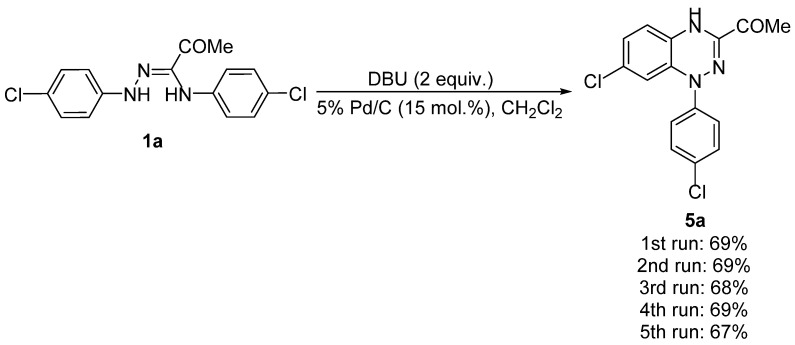
Recyclability of the Pd/C catalyst.

To further elucidate the synthetic utility of the developed protocol, we conducted a set of experiments on recycling of the Pd/C catalyst on the example of oxidative cyclization of amidrazone **1a**. After the reaction was completed, the catalyst was filtered off, washed with organic solvents, and dried at 100 °C for 24 h until the constant mass in each case. It was found that the catalyst could be reused at least 5 times without loss of activity, which was confirmed by nearly equal yields of benzo[1,2,4]triazine **5a** (Scheme 8).

Since benzo[1,2,4]triazine derivatives are of special importance in the preparation of functional organic materials, we also studied the thermal behavior of the synthesized compounds using differential scanning calorimetry (DSC). Thermal stability is a crucial parameter which strictly defines the applicability of materials in a construction of functional devices or molecular grafting. To our delight, all synthesized benzo[1,2,4]triazines, except formyl-derived Blatter radical **6k** and tricycle **7**, were thermally stable up to 240–250 °C (for DSC curves, see SI). It should also be pointed out that no phase transitions or mass loss were observed during DSC studies, which strongly support the application potential of the synthesized compounds in material science.

## 3. Conclusions

In summary, a divergent approach toward the construction of 1,4-dihydrobenzo[*e*][1,2,4]triazines bearing functional moieties at the triazine ring via DBU-catalyzed aerial oxidation of amidrazones in the presence of Pd/C was realized. It was found that the synthesized 1,4-dihydrobenzo[*e*][1,2,4]triazines are substantially resistible towards various oxidants, which is attributed to the strong electron-withdrawing effect of the functional groups incorporated in the heterocyclic system. In addition, the dihydrobenzo[*e*][1,2,4]triazolo[3,4-*c*][1,2,4]triazine open-shell compound was also prepared for the first time as a minor product. Recyclability of the Pd/C catalyst was also demonstrated by conducting the oxidative cyclization for at least five times on the same substrate. The majority of the synthesized fused heterocyclic systems exhibited high thermal stability, which further enables their application potential in material science and related fields.

## 4. Materials and Methods

**General**. All reactions were carried out in well-cleaned oven-dried glassware with magnetic stirring. ^1^H and ^13^C NMR spectra were recorded on a Bruker AM-300 (300.13 and 75.47 MHz, respectively) spectrometer and referenced to a residual solvent peak. The chemical shifts are reported in ppm (δ); multiplicities are indicated by s (singlet), d (doublet), t (triplet), q (quartet), m (multiplet) and br (broad). Coupling constants, *J*, are reported in Hertz. The IR spectra were recorded on a Bruker “Alpha” spectrometer in the range 400–4000 cm^−1^ (resolution 2 cm^−1^). Elemental analyses were performed by the CHN Analyzer Perkin-Elmer 2400. High resolution mass spectra were recorded on a Bruker microTOF spectrometer with electrospray ionization (ESI). All measurements were performed in a positive (+MS) ion mode (interface capillary voltage: 4500 V) with scan range *m/z*: 50–3000. External calibration of the mass spectrometer was performed with Electrospray Calibrant Solution (Fluka). A direct syringe injection was used for all analyzed solutions in MeCN (flow rate: 3 μL min^−1^). Nitrogen was used as nebulizer gas (0.4 bar) and dry gas (4.0 L min^−1^); interface temperature was set at 180 °C. All spectra were processed by using Bruker DataAnalysis 4.0 software package. Thermal behaviour of the synthesized compounds was studied using differential scanning calorimeter Netzsch DSC 204 HP. Analytical thin-layer chromatography (TLC) was carried out on Merck 25 TLC silica gel 60 F_254_ aluminum sheets. The visualization of the TLC plates was accomplished with a UV light. All solvents were purified and dried using standard methods prior to use. All standard reagents were purchased from Aldrich or Acros Organics and used without further purification. Initial chlorohydrazones **4** were prepared according to previously published procedures [30]. Preparation of amidrazones **1** was accomplished similarly to the procedure reported in [31].

**Synthesis of amidrazones 1a-l (general procedure)**. Et_3_N (1 mL, 7 mmol) was added to a magnetically stirred mixture of the corresponding chlorohydrazone **4** (5 mmol) and substituted aniline (5 mmol) in absolute EtOH (10 mL) at 20 °C. The reaction mixture was refluxed until the consumption of substrate **4** (TLC monitoring, eluent—CHCl_3_), and then cooled to 20 °C. If the crude product precipitated from the solution (in the case of compounds **1a,b,d,h,k**), it was filtered off, washed with water (2 × 5 mL) and 50% EtOH (1 × 4 mL), dried in air and recrystallized from 95% EtOH. If the precipitate did not form, the solvent was evaporated to dryness and the residue was triturated with water until the precipitation did not occur. The solid formed was filtered off, washed with water (2 × 5 mL) and 50% EtOH (1 × 4 mL), dried in air, and recrystallized from 95% EtOH.

*1-(4-Chlorophenylamino)-1-(4-chlorophenylhydrazono)-2-propanone* (**1a**). Yellow solid, yield 1.47 g (91%), mp 102–103 °C, R_f_ 0.52 (CHCl_3_). IR (KBr): 3354, 3281, 1660, 1581, 1489, 1361, 1091 cm^−1^. ^1^H NMR (300 MHz, [D_6_]DMSO): 2.50 (s, 3H), 6.58 (d, *J* 8.5 Hz, 2H), 7.20 (d, *J* 8.5 Hz, 2H), 7.33 (s, 4H), 8.02 (s, 1H), 9.91 (s, 1H). ^13^C NMR (75.5 MHz, [D_6_]DMSO): 24.9, 115.5, 117.3, 122.7, 124.5, 128.3, 128.9, 135.9, 141.3, 142.8, 192.9. Anal. calcd. for C_15_H_13_Cl_2_N_3_O (%): C, 55.92; H, 4.07; N, 13.04. Found (%): C, 56.06; H, 4.24; N, 12.81.

*1-(4-Chlorophenylamino)-1-(4-tolylhydrazono)-2-propanone* (**1b**). Yellowish solid, yield 1.06 g (74%), mp 133–134 °C, R_f_ 0.48 (CHCl_3_). IR (KBr): 3349, 3320, 1672, 1582, 1523, 1495, 1350, 1184 cm^−1^. ^1^H NMR (300 MHz, CDCl_3_): 2.34 (s, 3H), 2.62 (s, 3H), 6.57 (d, *J* 8.5 Hz, 2H), 7.04 (d, *J* 8.2 Hz, 2H), 7.14 (d, *J* 8.2 Hz, 2H), 7.22 (d, *J* 8.5 Hz, 2H), NH protons are significantly broadened. ^13^C NMR (75.5 MHz, CDCl_3_): 20.7, 23.9, 113.9, 118.7, 126.9, 129.2, 129.9, 131.9, 134.9, 138.0, 140.5, 193.8. Anal. calcd. for C_16_H_16_ClN_3_O (%): C, 63.68; H, 5.34; N, 13.92. Found (%): C, 63.52; H, 5.52; N, 13.69.

*1-(4-Nitrophenylamino)-1-phenylhydrazono-2-propanone* (**1c**). Yellow solid, yield 1.02 g (69%), mp 167–168 °C, R_f_ 0.54 (CHCl_3_). IR (KBr): 3357, 3277, 1674, 1601, 1555, 1508, 1463, 1339, 1111, cm^−1^. ^1^H NMR (300 MHz, CDCl_3_): 2.64 (s, 3H), 6.64 (d, *J* 8.8 Hz, 2H), 7.07 (t, *J* 7.3 Hz, 1H), 7.17–7.21 (m, 2H), 7.36 (t, *J* 7.3 Hz, 2H), 8.14 (d, *J* 8.8 Hz, 2H), NH protons are significantly broadened. ^13^C NMR (75.5 MHz, CDCl_3_): 24.1, 114.3, 115.9, 123.2, 125.7, 129.6, 133.6, 141.7, 142.3, 146.0, 193.6. Anal. calcd. for C_15_H_14_N_4_O_3_ (%): C, 60.40; H, 4.73; N, 18.78. Found (%): C, 60.23; H, 4.90; N, 18.52.

*1-(4-Nitrophenylhydrazono)-1-phenylamino-2-propanone* (**1d**). Yellowish solid, yield 1.00 g (67%), mp 162–163 °C, R_f_ 0.47 (CHCl_3_). IR (KBr): 3352, 3274, 1677, 1594, 1496, 1328, 1164, 1108 cm^−1^. ^1^H NMR (300 MHz, CDCl_3_): 2.66 (s, 3H), 6.74 (d, *J* 8.8 Hz, 2H), 7.06–7.11 (m, 4H), 7.29–7.35 (m, 2H), 7.51 (s, 1H), 8.18 (d, *J* 8.8 Hz, 2H), another NH proton is significantly broadened. ^13^C NMR (75.5 MHz, CDCl_3_): 24.0, 112.9, 118.9, 123.3, 126.0, 129.5, 137.2, 138.0, 141.7, 148.0, 194.2. Anal. calcd. for C_15_H_14_N_4_O_3_ (%): C, 60.40; H, 4.73; N, 18.78. Found (%): C, 60.62; H, 4.66; N, 18.55.

*1-(4-Nitrophenylhydrazono)-1-(4-tolylamino)-2-propanone* (**1e**). Yellowish solid, yield 1.29 g (83%), mp 154–155 ^o^C, R_f_ 0.56 (CHCl_3_). IR (KBr): 3384, 3258, 1672, 1592, 1513, 1479, 1327, 1158, 1108 cm^−1^. ^1^H NMR (300 MHz, CDCl_3_): 2.34 (s, 3H), 2.65 (s, 3H), 6.68 (d, *J* 8.7 Hz, 2H), 7.04 (d, *J* 7.7 Hz, 2H), 7.13 (d, *J* 7.7 Hz, 2H), 7.49 (br.s, 1H), 8.17 (d, *J* 8.7 Hz, 2H). ^13^C NMR (75.5 MHz, CDCl_3_): 20.8, 24.0, 112.7, 119.5, 126.0, 130.0, 133.3, 135.3, 137.4, 141.5, 148.1, 194.2. Anal. calcd. for C_16_H_16_N_4_O_3_ (%): C, 61.53; H, 5.16; N, 17.94. Found (%): C, 61.75; H, 5.01; N, 17.71.

*1-(4-Chlorophenylamino)-1-[4-(3-fluorophenyl)-1,2,5-oxadiazolyl-3-yl]hydrazono-2-propanone* (**1f**). Creme solid, yield 1.31 g (70%), mp 174–175 °C, R_f_ 0.17 (CHCl_3_). IR (KBr): 3322, 1691, 1594, 1550, 1483, 1089 cm^−1^. ^1^H NMR (300 MHz, [D_6_]DMSO): 2.28 (s, 3H), 6.75 (d, *J* 5.8 Hz, 2H), 7.28–7.41 (m, 6H), 8.57 (s, 1H), 9.56 (s, 1H). ^13^C NMR (75.5 MHz, [D_6_]DMSO): 24.6, 115.2 (d, *J* 23.8 Hz), 117.2 (d, *J* 20.9 Hz), 119.8, 124.4 (d, *J* 2.6 Hz), 124.7, 127.4 (d, *J* 9.0 Hz), 128.1, 130.7 (d, *J* 8.4 Hz), 138.6, 140.4, 146.5 (d, *J* 3.1 Hz), 153.9, 161.9 (d, *J* 245.2 Hz), 193.7. Anal. calcd. for C_17_H_13_ClFN_5_O_2_ (%): C, 54.63; H, 3.51; N, 18.74. Found (%): C, 54.39; H, 3.39; N, 18.63.

*1-[4-(3-Chlorophenyl)-1,2,5-oxadiazolyl-3-yl]hydrazono-1-(4-chlorophenylamino)-2-propanone* (**1g**). Creme solid, yield 1.23 g (63%), mp 162–163 °C, R_f_ 0.15 (CHCl_3_). IR (KBr): 3316, 1687, 1594, 1549, 1506, 1270, 1077 cm^−1^. ^1^H NMR (300 MHz, [D_6_]DMSO): 2.25 (s, 3H), 6.74 (d, *J* 8.0 Hz, 2H), 7.29 (d, *J* 8.0 Hz, 2H), 7.44–7.50 (m, 2H), 7.60–7.65 (m, 2H), 8.54 (s, 1H), 9.68 (s, 1H). ^13^C NMR (75.5 MHz, [D_6_]DMSO): 24.6, 119.6, 124.6, 126.9, 127.5, 128.0, 128.2, 130.1, 130.4, 133.4, 138.7, 140.4, 146.5, 154.0, 193.7. Anal. calcd. for C_17_H_13_Cl_2_N_5_O_2_ (%): C, 52.33; H, 3.36; N, 17.95. Found (%): C, 52.49; H, 3.23; N, 17.73.

*1-(4-Chlorophenylamino)-1-[(4-cyclohexyl-1,2,5-oxadiazol-3-yl)hydrazono]-2-propanone* (**1h**). Slightly orange solid, yield 0.99 g (55%), mp 97–98 °C, R_f_ 0.38 (CHCl_3_). IR (KBr): 3325, 2933, 2856, 1691, 1586, 1495, 1402, 1357, 1088 cm^−1^. ^1^H NMR (300 MHz, [D_6_]DMSO): 1.27–1.43 (m, 5H), 1.62–1.90 (m, 5H), 2.49 (s, 3H), 3.03 (t, *J* 7.5 Hz, 1H), 6.70 (d, *J* 6.8 Hz, 2H), 7.24 (d, *J* 6.8 Hz, 2H), 8.45 (s, 1H), 9.74 (s, 1H). ^13^C NMR (75.5 MHz, [D_6_]DMSO): 25.1, 25.3, 25.4, 30.7, 33.0, 119.4, 124.2, 128.0, 139.2, 139.8, 152.1, 153.8, 193.4. Anal. calcd. for C_17_H_20_ClN_5_O_2_ (%): C, 56.43; H, 5.57; N, 19.36. Found (%): C, 56.67; H, 5.45; N, 19.02.

*Ethyl 2-(4-chlorophenylamino)-2-(4-nitrohydrazono)acetate* (**1i**). Orange solid, yield 1.50 g (82%), mp 198–199 °C, R_f_ 0.25 (CHCl_3_). IR (KBr): 3403, 3278, 1708, 1591, 1532, 1489, 1322, 1110, 1009 cm^−1^. ^1^H NMR (300 MHz, CDCl_3_): 1.50 (t, *J* 7.1 Hz, 3H), 4.51 (q, *J* 7.1 Hz, 2H), 7.03 (d, *J* 8.8 Hz, 2H), 7.33 (d, *J* 8.7 Hz, 2H), 7.49 (d, *J* 8.7 Hz, 2H), 8.18 (d, *J* 8.8 Hz, 2H), 11.19 (s, 1H), another NH proton is significantly broadened. ^13^C NMR (75.5 MHz, CDCl_3_): 14.2, 63.4, 111.5, 113.2, 119.6, 126.0, 126.4, 129.1, 129.6, 137.9, 149.4, 159.4. Anal. calcd. for C_16_H_15_ClN_4_O_4_ (%): C, 52.97; H, 4.17; N, 15.44. Found (%): C, 53.20; H, 4.01; N, 15.22.

*Ethyl 2-(4-nitrophehylhydrazono)-2-(4-tolylamino)acetate* (**1j**). Orange solid, yield 1.23 g (72%), mp 162–163 °C, R_f_ 0.27 (CHCl_3_). IR (KBr): 3410, 3282, 1709, 1605, 1532, 1491, 1321, 1113 cm^−1^. ^1^H NMR (300 MHz, CDCl_3_): 1.45 (t, *J* 6.7 Hz, 3H), 2.37 (s, 3H), 4.45 (q, *J* 6.7 Hz, 2H), 6.71 (d, *J* 7.7 Hz, 1H), 7.13 (d, *J* 7.7 Hz, 1H), 7.20 (d, *J* 8.0 Hz, 1H), 7.42 (d, *J* 8.0 Hz, 1H), 7.65 (br s, 1H), 11.04 (br s, 1H). ^13^C NMR (75.5 MHz, CDCl_3_): 14.2, 20.9, 63.3, 111.4, 112.9, 118.9, 119.0, 125.9, 126.3, 129.7, 130.1, 131.8, 132.5, 133.4, 135.4, 136.5, 140.0, 141.5, 148.4, 149.7, 159.42, 162.7. Anal. calcd. for C_17_H_18_N_4_O_4_ (%): C, 59.64; H, 5.30; N, 16.37. Found (%): C, 59.38; H, 5.54; N, 16.13.

*Ethyl 2-(4-tolylamino)-2-(4-tolylhydrazono)acetate* (**1k**). Brown solid, yield 1.01 g (65%), mp 69–70 °C, R_f_ 0.49 (CHCl_3_). IR (KBr): 3359, 3291, 1702, 1567, 1512, 1472, 1370, 1105, 1021 cm^−1^. ^1^H NMR (300 MHz, [D_6_]DMSO): 1.19 (t, *J* 7.0 Hz, 3H), 2.21 (s, 3H), 2.23 (s, 3H), 4.15 (q, *J* 7.0 Hz, 2H), 6.54 (d, *J* 8.1 Hz, 2H), 7.01 (d, *J* 8.1 Hz, 2H), 7.04–7.12 (m, 4H), 7.72 (s, 1H), 9.39 (s, 1H). ^13^C NMR (75.5 MHz, [D_6_]DMSO): 14.1, 14.4, 20.6, 62.1, 111.4, 119.0, 126.3, 129.7, 132.5, 135.4, 140.0, 148.4, 159.4, 162.7. Anal. calcd. for C_18_H_21_N_3_O_2_ (%): C, 69.43; H, 6.80; N, 13.49. Found (%): C, 69.26; H, 7.02; N, 13.22.

*Ethyl 2-(4-chlorophenylamino)-2-(4-chlorophenylhydrazono)acetate* (**1l**). Pale yellow solid, yield 1.49 g (85%), mp 94–95 °C, R_f_ 0.52 (CHCl_3_). IR (KBr): 3351, 3299, 1704, 1595, 1567, 1490, 1278, 1091 cm^−1^. ^1^H NMR (300 MHz, CDCl_3_): 1.42 (t, 3H, *J* 7.0 Hz, CH_3_), 4.39 (q, 2H, *J* 7.0 Hz, CH_2_), 6.63 (d, *J* 8.4 Hz, 2H), 6.99–7.06 (m, 2H), 7.23–7.42 (m, 5H), 10.23 (br s, 1H, exchangeable proton). ^13^C NMR (75.5 MHz, CDCl_3_): 14.3, 62.2, 112.9, 114.0, 118.1, 119.0, 126.8, 129.0, 129.3, 129.8, 138.5, 163.3. Anal. calcd. for C_16_H_15_Cl_2_N_3_O_2_ (%): C, 54.56; H, 4.29; N, 11.93. Found (%): C, 54.35; H, 4.47; N, 11.65.

**Synthesis of benzo[1,2,4[triazine derivatives 5–7 (general procedure)**. DBU (0.3 mL, 2 mmol) and 5% Pd/C (15 mol.%, 640 mg, 0.3 mmol) were added to a magnetically stirred mixture of amidrazone **1** (2 mmol) in CH_2_Cl_2_ (4 mL) at 25 °C. The reaction mixture was stirred at 25 °C until the consumption of starting amidrazone **1** (TLC monitoring, eluent—CHCl_3_), then Pd/C was filtered off and thoroughly washed with CH_2_Cl_2_ until the filtrate became completely colorless. The solvent was evaporated, and the residue was purified by preparative TLC (eluent—CHCl_3_).

*3-Acetyl-7-chloro-1-(4-chlorophenyl)-1,4-dihydrobenzo[e][1,2,4]triazine* (**5a**). Red solid, yield 0.442 g (69%), mp 198 °C, T_d_ 254 °C, R_f_ 0.67 (CHCl_3_). IR (KBr): 3336, 1678, 1631, 1485, 1363, 1061 cm^−1^. ^1^H NMR (300 MHz, CDCl_3_): 2.41 (s, 3H), 6.27–6.31 (m, 2H), 6.40 (br s, 1H), 6.71 (dd, *J* 6.3, 1.9 Hz, 1H), 7.37–7.44 (m, 4H). ^13^C NMR (75.5 MHz, CDCl_3_): 23.7, 112.7, 114.3, 124.0, 124.3, 129.1, 129.6, 130.9, 131.3, 133.7, 141.0, 143.8, 191.1. HRMS (ESI) [M + H]^+^ *m*/*z* calcd. for C_15_H_11_^35^Cl_2_N_3_O**^.^**: 319.0266, found: 319.0274; calcd. for C_15_H_11_^35^Cl^37^ClN_3_O**^.^**: 321.0243, found: 321.0245.

*3-Acetyl-7-chloro-1-(4-p-tolyl)-1,4-dihydrobenzo[e][1,2,4]triazine* (**5b**). Purple solid, yield 0.461 g (77%), mp 170 °C, T_d_ 255 °C, R_f_ 0.50 (CHCl_3_). IR (KBr): 3342, 1675, 1493, 1362, 1291, 1067 cm^−1^. ^1^H NMR (300 MHz, [D_6_]DMSO): 2.29 (s, 3H), 2.34 (s, 3H), 5.94 (s, 1H), 6.63–6.71 (m, 2H), 7.29 (br s, 4H), 8.53 (s, 1H). ^13^C NMR (75.5 MHz, [D_6_]DMSO): 20.5, 23.8, 110.8, 114.6, 123.1, 123.3, 126.7, 129.9, 132.4, 134.8, 135.1, 139.9, 144.6, 190.6. Anal. calcd. for C_16_H_14_ClN_3_O (%): C, 64.11; H, 4.71; N, 14.02. Found (%): C, 63.89; H, 4.88; N 13.78.

*3-Acetyl-7-nitro-1-phenyl-1,4-dihydrobenzo[e][1,2,4]triazine* (**5c**). Pale brown solid, yield 0.300 g (51%), T_d_ 272 °C (decomposed without melting), R_f_ 0.27 (CHCl_3_). IR (KBr): 3334, 1679, 1573, 1506, 1371, 1315, 1087 cm^−1^. ^1^H NMR (300 MHz, [D_6_]DMSO): 2.29 (s, 3H), 6.68–6.71 (m, 3H), 7.34 (t, *J* 6.7 Hz, 1H), 7.45–7.60 (m, 4H), 9.24 (s, 1H). ^13^C NMR (75.5 MHz, [D_6_]DMSO): 23.7, 104.6, 112.3, 112.5, 122.0, 123.3, 126.3, 129.7, 133.7, 141.6, 141.7, 142.9, 143.3, 190.1. HRMS (ESI) Calcd. for: C_15_H_12_N_4_O_3_**^.^** [M**^.^**+ H]^+^: 296.0904, found: 296.0897.

*3-Acetyl-1-(4-nitrophenyl)-1,4-dihydrobenzo[e][1,2,4]triazine* (**5d**). Brown solid, yield 0.249 g (42%), mp 219 °C, T_d_ 256 °C, R_f_ 0.47 (CHCl_3_). IR (KBr): 3349, 1682, 1585 1490, 1448, 1371, 1339, 1283, 1059 cm^−1^. ^1^H NMR (300 MHz, [D_6_]DMSO): 2.46 (s, 3H), 6.86–6.98 (m, 4H), 7.63 (d, *J* 9.1 Hz, 2H), 8.22 (d, *J* 9.1 Hz, 2H), 9.16 (s, 1H). ^13^C NMR (75.5 MHz, [D_6_]DMSO): 24.3, 114.7, 115.5, 116.4, 123.6, 125.4, 126.1, 127.4, 134.8, 140.3, 147.4, 147.7, 191.3. Anal. calcd. for C_15_H_12_N_4_O_3_ (%): C, 60.81; H, 4.08; N, 18.91. Found (%): C, 61.03; H, 3.94; N, 18.68.

*3-Acetyl-1-(4-nitrophenyl)-7-methyl-1,4-dihydrobenzo[e][1,2,4]triazine* (**5e**). Dark-brown solid, yield 0.415 g (67%), mp 252 °C, T_d_ 252 °C, R_f_ 0.44 (CHCl_3_). IR (KBr): 3342, 1682, 1586, 1505, 1331, 1284, 1066 cm^−1^. ^1^H NMR (300 MHz, [D_6_]DMSO): 2.14 (s, 3H), 2.46 (s, 3H), 6.76–6.87 (m, 3H), 7.63 (d, *J* 9.2 Hz, 2H), 8.22 (d, *J* 9.2 Hz, 2H), 9.10 (s, 1H). ^13^C NMR (75.5 MHz, [D_6_]DMSO): 20.4, 24.3, 115.2, 115.3, 116.4, 125.4, 126.3, 127.3, 132.0, 132.9, 140.2, 147.4, 147.7, 191.4. Anal. calcd. for C_16_H_14_N_4_O_3_ (%): C, 61.93; H, 4.55; N, 18.06. Found (%): C, 61.65; H, 4.74; N, 17.82.

*3-Acetyl-1-[4-(3-fluorophenyl)-1,2,5-oxadiazol-3-yl]-7-chloro-1,4-dihydrobenzo[e][1,2,4]triazine* (**5f**). Orange solid, yield 0.379 g (51%), mp 159 °C, T_d_ 254 °C, R_f_ 0.42 (CHCl_3_). IR (KBr): 3306, 1708, 1597, 1530, 1505, 1476, 1410, 1266 cm^−1^. ^1^H NMR (300 MHz, [D_6_]DMSO): 1.55 (s, 3H), 6.71 (d, *J* 8.3 Hz, 1H), 6.88 (dd, *J* 6.2, 2.1 Hz, 1H), 7.28 (br s, 1H), 7.36–7.42 (m, 1H), 7.57–7.65 (m, 3H), 9.05 (s, 1H). ^13^C NMR (75.5 MHz, [D_6_]DMSO): 22.7, 114.8, 115.2, 115.8 (d, *J* 23.6 Hz), 116.7 (d, *J* 20.9 Hz), 125.0, 125.1 (d, *J* 3.0 Hz), 126.8, 128.4, 128.6, 128.7, 130.4 (d, *J* 8.5 Hz), 131.3, 145.2, 148.7 (d, *J* 2.7 Hz), 152.9, 161.7 (d, *J* 244.5 Hz), 190.4. Anal. calcd. for C_17_H_11_ClFN_5_O_2_ (%): C, 54.92; H, 2.98; N, 18.84. Found (%): C, 54.70; H, 3.12; N, 18.57.

*3-Acetyl-1-[4-(3-chlorophenyl)-1,2,5-oxadiazol-3-yl]-7-chloro-1,4-dihydrobenzo[e][1,2,4]triazine* (**5g**). Red solid, yield 0.419 g (54%), mp 179 °C, T_d_ 256 °C, R_f_ 0.50 (CHCl_3_). IR (KBr): 3363, 1697, 1651, 1495, 1411, 1362, 1080 cm^−1^. ^1^H NMR (300 MHz, [D_6_]DMSO): 1.54 (s, 3H), 6.71 (d, *J* 8.3 Hz, 1H), 6.87 (dd, *J* 6.3, 2.0 Hz, 1H), 7.32 (s, 1H), 7.54–7.63 (m, 2H), 7.70–7.73 (m, 1H), 7.86 (s, 1H), 9.06 (s, 1H). ^13^C NMR (75.5 MHz, [D_6_]DMSO): 22.7, 114.9, 115.3, 125.0, 126.8, 127.6, 128.6, 128.7 (2 C), 129.7, 130.2, 131.4, 133.0, 145.3, 148.6, 152.8, 190.4. Anal. calcd. for C_17_H_11_Cl_2_N_5_O_2_ (%): C, 52.60; H, 2.86; N, 18.04. Found (%): C, 52.34; H, 3.02; N, 17.78.

*3-Acetyl-1-[4-cyclohexyl-1,2,5-oxadiazol-3-yl]-7-chloro-1,4-dihydrobenzo[e][1,2,4]triazine* (**5h**). Carmine solid, yield 0.575 g (80%), mp 218 °C, R_f_ 0.47 (CHCl_3_). IR (KBr): 3315, 2928, 2863, 1699, 1594, 1499, 1306, 1073 cm^−1^. ^1^H NMR (300 MHz, [D_6_]DMSO): 1.32–1.81 (m, 8H), 2.10 (d, *J* 12.1 Hz, 2H), 2.32 (s, 3H), 3.13–3.23 (m, 1H), 6.70–6.74 (m, 1H), 6.85 (dd, *J* 6.2, 2.1 Hz, 1H), 7.21 (d, *J* 1.9 Hz, 1H), 9.06 (s, 1H). ^13^C NMR (75.5 MHz, [D_6_]DMSO): 24.2, 25.3, 25.5, 31.2, 34.1, 114.6, 115.2, 124.9, 126.8, 129.2, 131.6, 145.9, 152.8, 154.4, 190.4. Anal. calcd. for C_17_H_18_ClN_5_O_2_ (%): C, 56.75; H, 5.04; N, 19.46. Found (%): C, 56.97; H, 4.83; N, 19.19.

*Ethyl 7-chloro-1-(4-nitrophenyl)-1,4-dihydrobenzo[e][1,2,4]triazine-3-carboxylate* (**5i**). Brown solid, yield 0.404 g (56%) mp 242 °C, T_d_ 242 °C, R_f_ 0.18 (CHCl_3_). IR (KBr): 3363, 3353, 1702, 1589, 1502, 1376, 1343, 1289, 1056 cm^−1^. ^1^H NMR (300 MHz, [D_6_]DMSO): 1.32 (t, *J* 7.1 Hz, 3H), 4.35 (q, *J* 7.1 Hz, 2H), 6.85 (s, 1H), 6.91 (d, *J* 8.3 Hz, 1H), 6.99 (d, *J* 8.3 Hz, 1H), 7.58 (d, *J* 9.1 Hz, 2H), 8.25 (d, *J* 9.1 Hz, 2H), 9.46 (s, 1H). ^13^C NMR (75.5 MHz, [D_6_]DMSO): 14.4, 62.9, 114.4, 116.7, 117.7, 125.8, 125.9, 128.0, 130.0, 134.2, 141.5, 143.5, 147.6, 159.6. Anal. calcd. for C_16_H_13_ClN_4_O_4_ (%): C, 53.27; H, 3.63; N, 15.53. Found (%): C, 52.98; H, 3.79; N, 15.29.

*Ethyl 7-methyl-1-(4-nitrophenyl)-1,4-dihydrobenzo[e][1,2,4]triazine-3-carboxylate* (**5j**). Dark red solid, yield 0.204 g (30%), mp 200 °C, T_d_ 252 °C, R_f_ 0.35 (CHCl_3_). IR (KBr): 3368, 3353, 1715, 1589, 1517, 1375, 1328, 1111 cm^−1^. ^1^H NMR (300 MHz, [D_6_]DMSO): 1.31 (t, *J* 7.1 Hz, 3H), 2.14 (s, 3H), 4.33 (q, *J* 7.1 Hz, 2H), 6.76–6.86 (m, 3H), 7.52 (d, *J* 9.3 Hz, 2H), 8.19 (d, *J* 9.3 Hz, 2H), 9.33 (s, 1H). ^13^C NMR (75.5 MHz, [D_6_]DMSO): 14.4, 21.0, 62.8, 115.8, 115.9, 116.5, 125.9, 126.8, 127.8, 132.6, 133.9, 140.5, 143.9, 148.0, 159.9. Anal. calcd. for C_17_H_16_N_4_O_4_ (%): C, 60.00; H, 4.74; N, 16.46. Found (%): C, 59.82; H, 4.87; N, 16.23.

*Ethyl 7-formyl-1-(4-p-tolyl)-1,4-dihydrobenzo[e][1,2,4]triazine-3-carboxylate* (**5k**). Dark-red solid, yield 0.252 g (39%), mp 224 °C, T_d_ 259 °C, R_f_ 0.21 (CHCl_3_). IR (KBr): 3217, 1718, 1659, 1569, 1504, 1433, 1376, 1323, 1109 cm^−1^. ^1^H NMR (300 MHz, [D_6_]DMSO): 1.26 (t, *J* 7.1 Hz, 3H), 2.36 (s, 3H), 4.26 (q, *J* 7.1 Hz, 2H), 6.25 (s, 1H), 6.72 (d, *J* 7.7 Hz, 1H), 7.22–7.32 (m, 5H), 9.09 (s, 1H), 9.48 (s, 1H). ^13^C NMR (75.5 MHz, [D_6_]DMSO): 13.9, 20.6, 61.9, 107.7, 112.8, 124.0, 130.1, 130.7, 132.5, 134.7, 135.6, 137.6, 140.1, 140.2, 159.2, 190.2. HRMS (ESI) Calcd. for C_18_H_17_N_3_NaO_3_ [M + Na]^+^: 346.1162, found: 346.1153.

*3-Ethoxycarbonyl-7-formyl-1-(4-p-tolyl)-1,4-dihydrobenzo[e][1,2,4]triazin-4-yl* (**6k**). Red-brown solid, yield 0.122 g (19%), mp 176 °C, T_d_ 191 °C, R_f_ 0.20 (CHCl_3_). IR (KBr): 3220, 1734, 1719, 1676, 1660, 1571, 1507, 1434, 1278, 1102 cm^−1^. EPR (toluene) *g* = 2.0034. HRMS (ESI) Calcd. for C_18_H_16_N_3_NaO_3_ [M + Na]^+^: 345.1099, found: 345.1097. Anal. calcd. for C_18_H_16_N_3_O_3_ (%): C, 67.07; H, 5.00; N, 13.04. Found (%): C, 66.83; H, 5.26; N, 12.78.

*3-Ethoxycarbonyl-7-chloro-1-(4-chlorophenyl)-1,4-dihydrobenzo[e][1,2,4]triazin-4-yl* (**6l**). Red-brown solid, yield 0.419 g (23%), mp 167 °C, T_d_ 254 °C, R_f_ 0.54 (CHCl_3_). IR (KBr): 3368, 1703, 1486, 1373, 1059 cm^−1^. EPR (toluene) *g* = 2.0035. HRMS (ESI) Calcd. for C_16_H_12_^35^Cl_2_N_3_NaO_2_ [M + Na]^+^: 371.0199, found: 371.0197; calcd. for C_16_H_12_^35^Cl^37^ClN_3_NaO_2_ [M + Na]^+^: 373.0170, found: 373.0175. Anal. calcd. for C_16_H_12_Cl_2_N_3_O_2_ (%): C, 55.03; H, 3.46; N, 12.03. Found (%): C, 54.79; H, 3.59; N, 11.79.

*7-Chloro-3,5-di(4-chlorophenyl)-1-(4-chlorophenylamino)-3,5-dihydro-benzo[e][1,2,4]triazolo [3,4-c][1,2,4]-triazinyl* (**7**). Gray solid, yield 0.083 g (15%), mp 194 °C, T_d_ 194 °C, R_f_ 0.60 (CHCl_3_). IR (KBr): 3397, 1681, 1621, 1594, 1490, 1403, 1093 cm^−1^. EPR (toluene) *g* = 2.0024. HRMS (ESI) Calcd. for C_26_H_16_^35^Cl_4_N_6_**^.^** [M**^.^**+ H]^+^: 552.0186, found: 552.0185; calcd. for C_26_H_16_^35^Cl_3_^37^ClN_6_**^.^**: 554.0159, found: 554.0159; calcd. for C_26_H_16_^35^Cl_2_^37^Cl_2_N_6_**^.^**: 556.0133, found: 556.0127. Anal. calcd. for C_26_H_16_Cl_4_N_6_ (%): C, 56.34; H, 2.91; N, 15.16. Found (%): C, 56.57; H, 2.79; N, 14.89.

**X-ray crystallographic data and refinement details**. Crystals of **7** suitable for X-ray diffraction were grown from DMSO-CH_2_Cl_2_ mixture (2:1). X-ray diffraction data were collected at 100K on a four-circle Rigaku Synergy S diffractometer equipped with a HyPix600HE area-detector (kappa geometry, shutterless ω-scan technique), using graphite monochromatized Cu Kα-radiation. The intensity data were integrated and corrected for absorption and decay by the CrysAlisPro program [32]. The structure was solved by direct methods using SHELXT [33] and refined on *F^2^* using SHELXL-2018 [34] in the OLEX2 program [35]. All non-hydrogen atoms were refined with individual anisotropic displacement parameters. The location of hydrogen atoms H14A and H14B were found from the electron density-difference map; these hydrogen atoms were refined with an individual isotropic displacement parameter. All other hydrogen atoms were placed in ideal calculated positions and refined as riding atoms with relative isotropic displacement parameters. The Mercury program suite [36] was used for molecular graphics. Three molecules of the solvent (DMSO) are disordered onto two positions. Deposition number 2160590 contain the supplementary crystallographic data. These data are provided free of charge by the joint Cambridge Crystallographic Data Centre.

## Figures and Tables

**Table 1:**
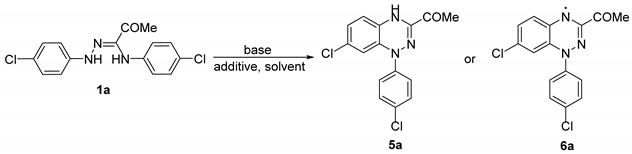
Optimization of oxidative cyclization of amidrazone **1a** ^a^.

Entry	Base (Equiv.)	Additive (mol.%)	Solvent	T, °C	Time, h	Product (Yield, %) ^b^
1	DBU (0.1)	5% Pd/C (1.6)	CH_2_Cl_2_	25	72	- ^c^
2	DBU (1)	-	CH_2_Cl_2_	25	72	- ^c^
3	DBU (1)	5% Pd/C (15)	CH_2_Cl_2_	25	10	**5a** (43)
4	DBU (1)	5% Pd/C (15)	CH_2_Cl_2_	40	7	**5a** (36)
5	DBU (2)	5% Pd/C (15)	CH_2_Cl_2_	25	8	**5a** (69)
6	Et_3_N (2)	5% Pd/C (15)	CH_2_Cl_2_	25	48	**5a** (29)
7	DBU (2)	C_act_ (200)	CH_2_Cl_2_	40	24	**5a** (35)
8	DBU (2)	TiO_2_ (50)	CH_2_Cl_2_	40	24	**5a** (8)
9	Cs_2_CO_3_ (1)	-	CH_2_Cl_2_	25	48	**5a** (10)
10	-	MnO_2_ (100)	CH_2_Cl_2_	25	72	- ^c^
11	DBU (2)	5% Pd/C (15)	MeCN	50	8	**5a** (23)

^a^ Reaction conditions: amidrazone **1a** (2 mmol), base, additive, solvent (4 mL), stirring at indicated temperature for indicated time. ^b^ Isolated yield of analytically pure product **5a** is given. ^c^ No reaction.

## Data Availability

Data obtained in this project are contained within this article and available upon request from the authors.

## Supplementary Materials

The following supporting information can be downloaded at: https://www.mdpi.com/article/10.3390/molecules27082575/s1, S1. Thermal behavior; S2. Crystallographic data (Tables S1–S7); S3. EPR spectra; S4. NMR and HRMS spectra.

## Sample Availability

Samples of the compounds are not available from the authors.
