# Supplementary material for: Revisiting the Synthesis of Functionally Substituted 1,4-Dihydrobenzo[e][1,2,4]triazines
Source: Molecules. 2022 Apr 15;27(8):2575. doi: 10.3390/molecules27082575 (PMC9031768; doi:10.3390/molecules27082575)

Supporting Information for

Revisiting the synthesis of functionally substituted 1,4-dihydrobenzo[*e*][1,2,4]triazines

Margarita A. Epishina, Alexander S. Kulikov, Leonid L. Fershtat*

Laboratory of Nitrogen Compounds, N.D. Zelinsky Institute of Organic Chemistry, Russian Academy of Sciences, Leninsky prosp., 47, 119991 Moscow, Russian Federation; fershtat@ioc.ac.ru (L.L.F.)

Table of Contents

S1. Thermal behavior 2

S2. Crystallographic data 9

S3. EPR spectra 38

S4. NMR and HRMS spectra 38

S1. Thermal behavior

Thermal behavior of the synthesized compounds was studied using differential scanning calorimeter Netzsch DSC 204 HP. The measurements were performed under normal pressure (0.1 MPa) in nitrogen flow 150 ml min^{-1}. The samples with mass near 1.0 mg were placed in closed aluminum crucibles with pierced lids and heated up to 450 °C at the linearly heating rate 5 $K{\cdot}min^{-1}$.

DSC curve for compound 5a

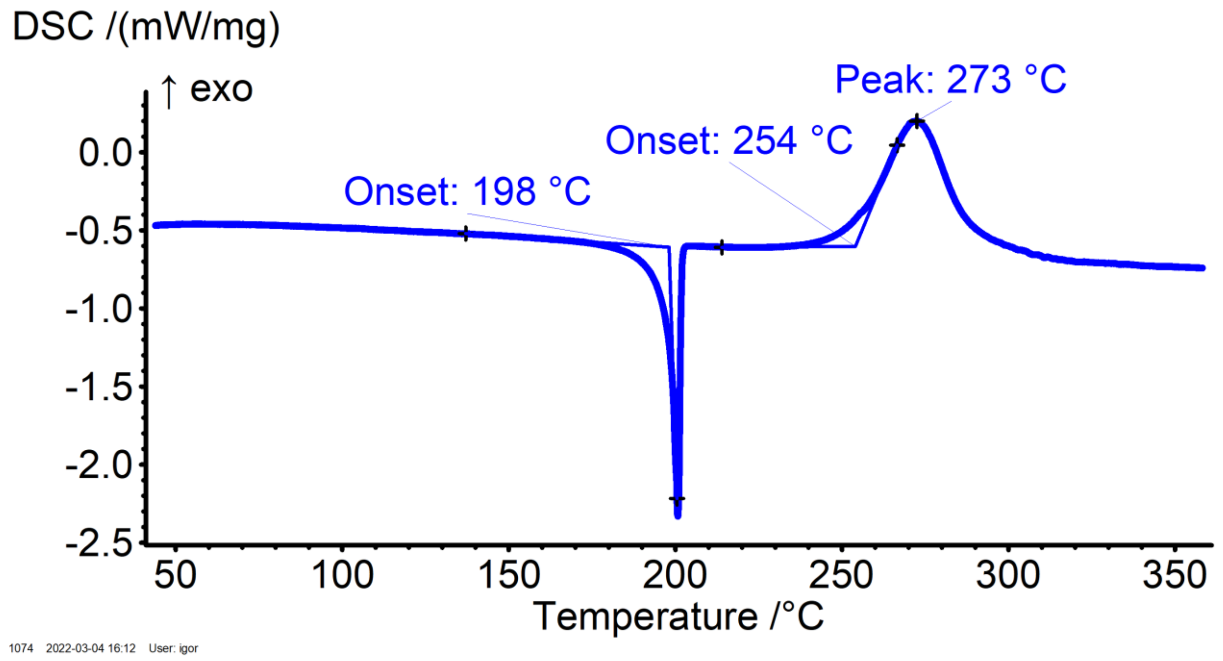

DSC curve for compound 5b

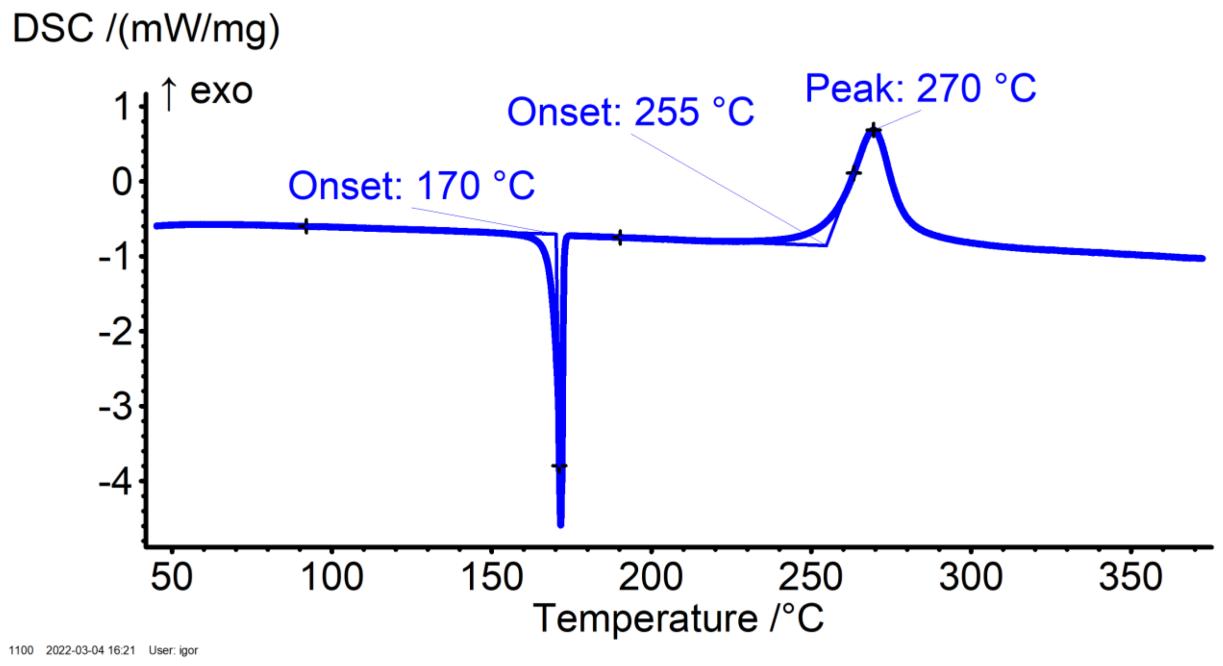

DSC curve for compound 5c

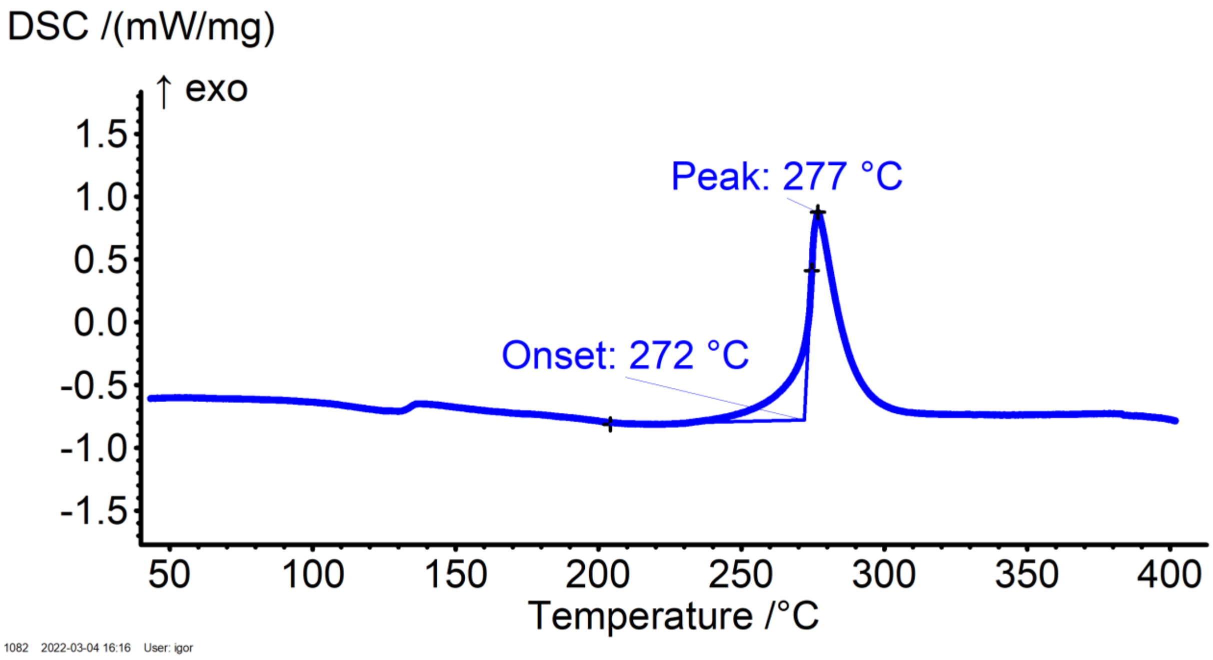

DSC curve for compound 5d

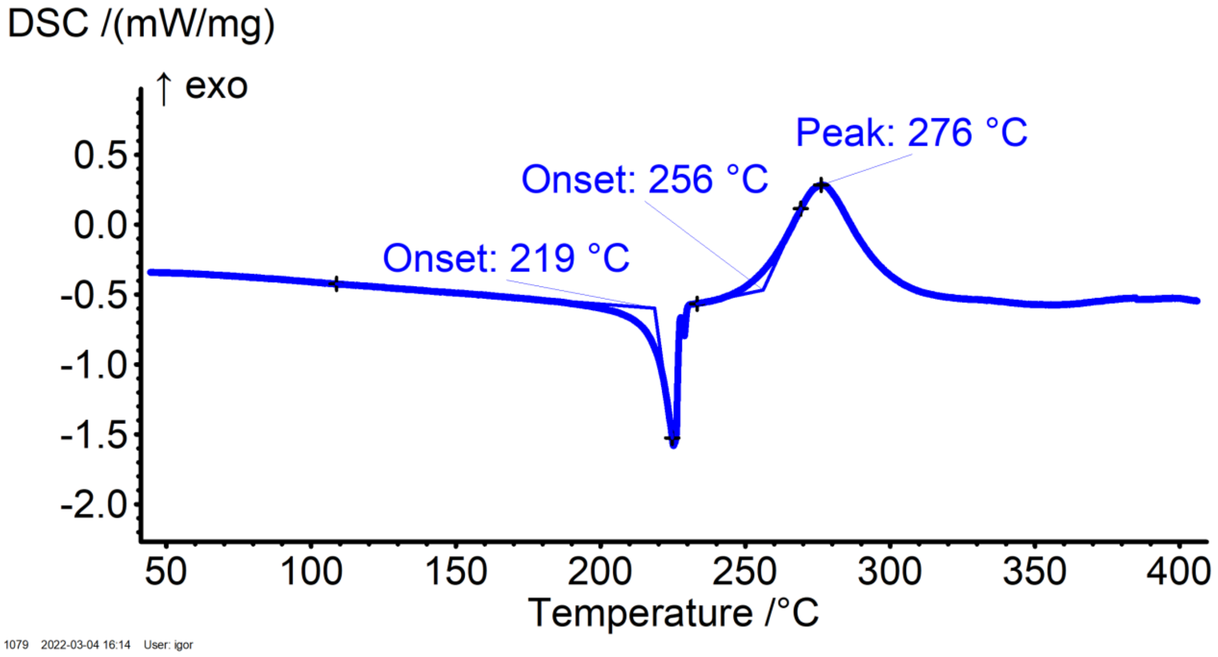

DSC curve for compound 5e

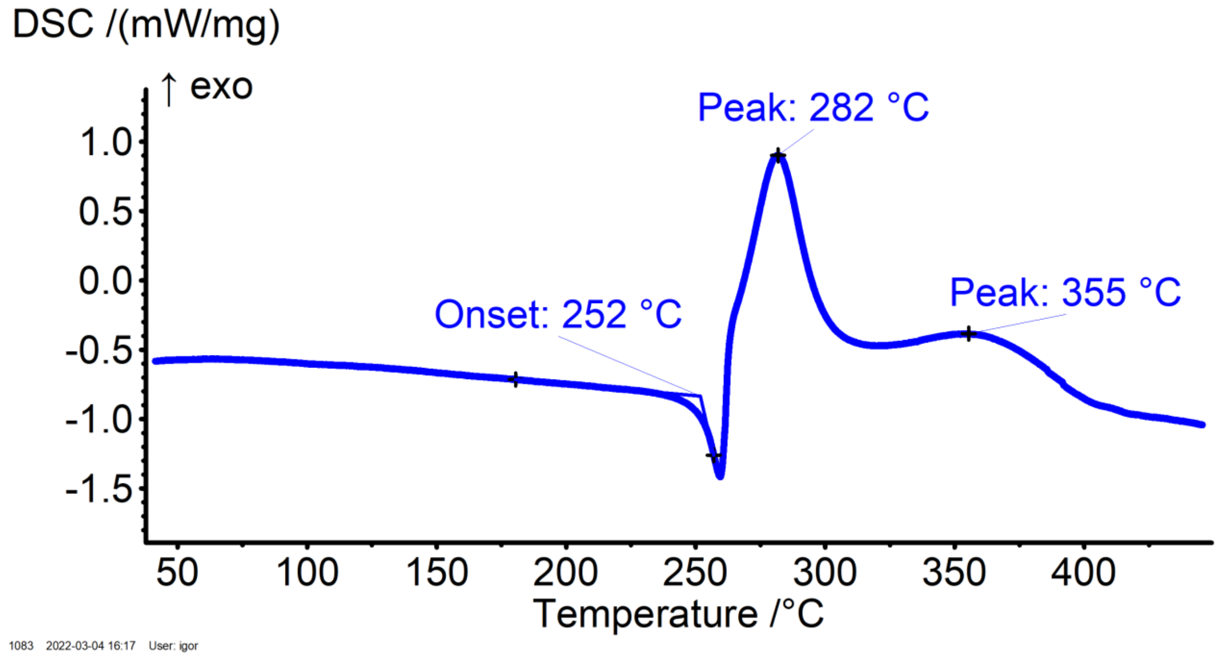

DSC curve for compound 5f

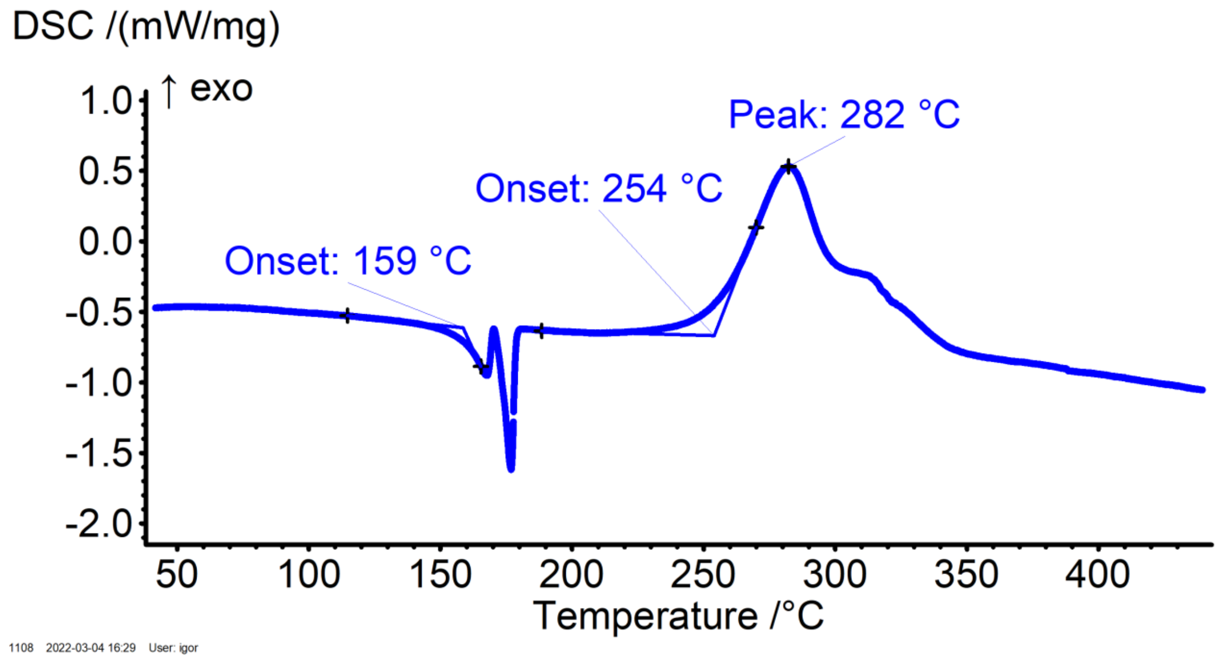

DSC curve for compound 5g

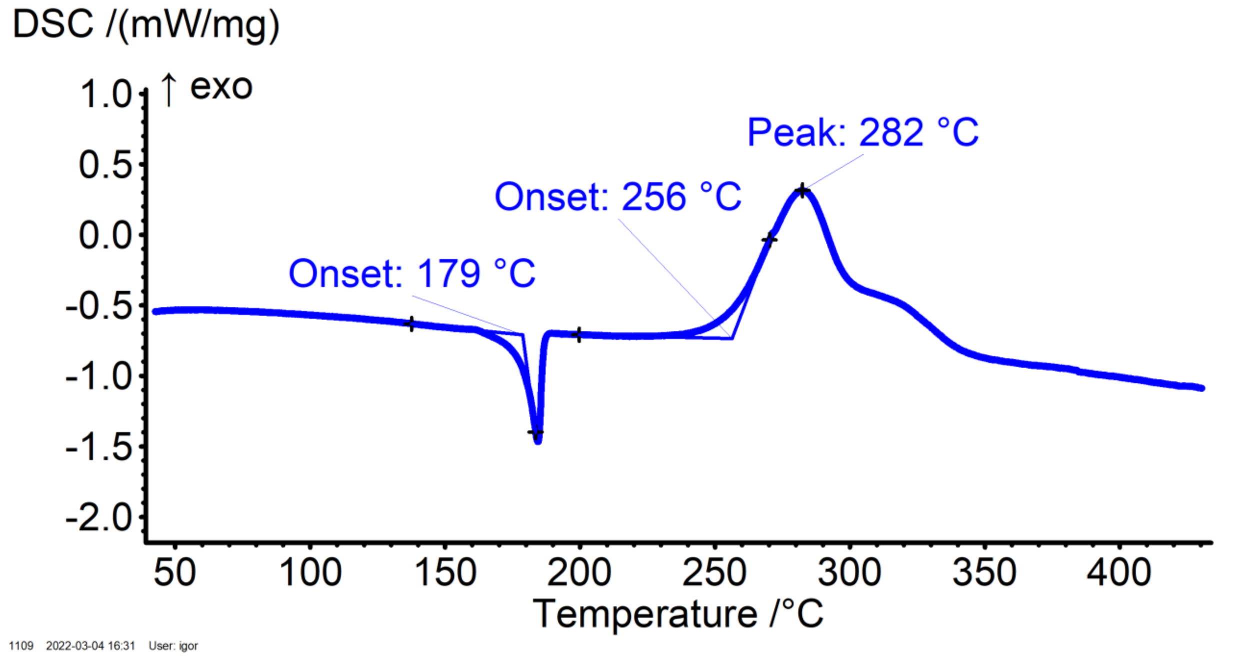

DSC curve for compound 5i

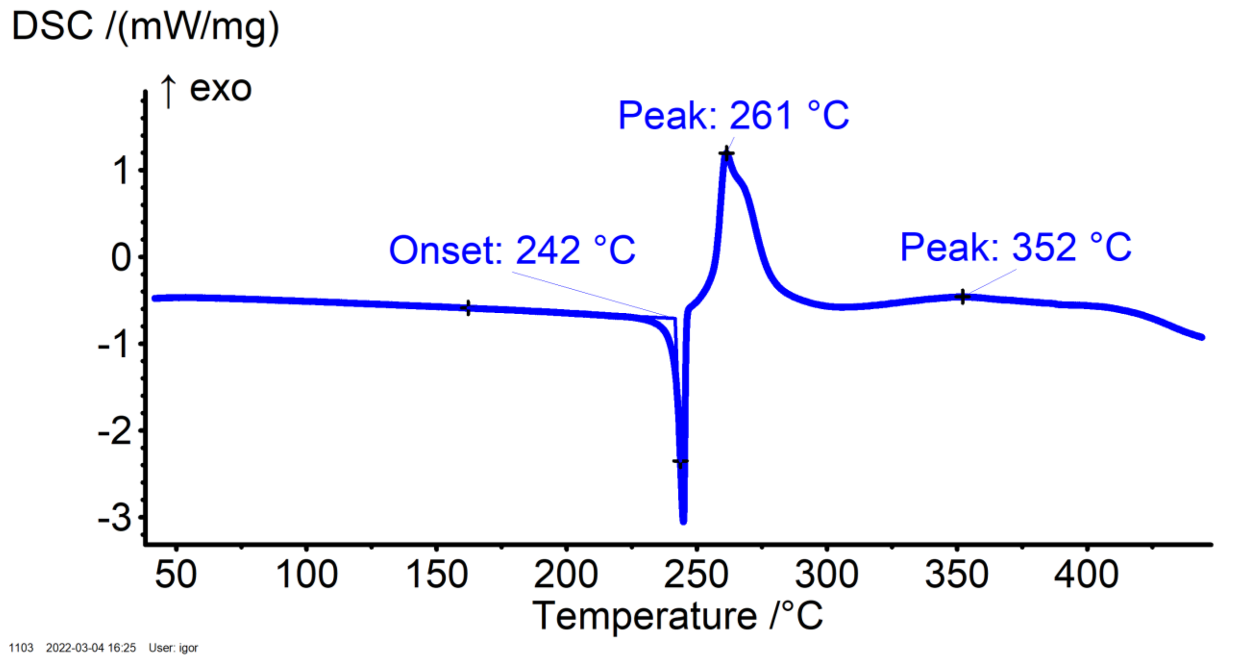

DSC curve for compound 5j

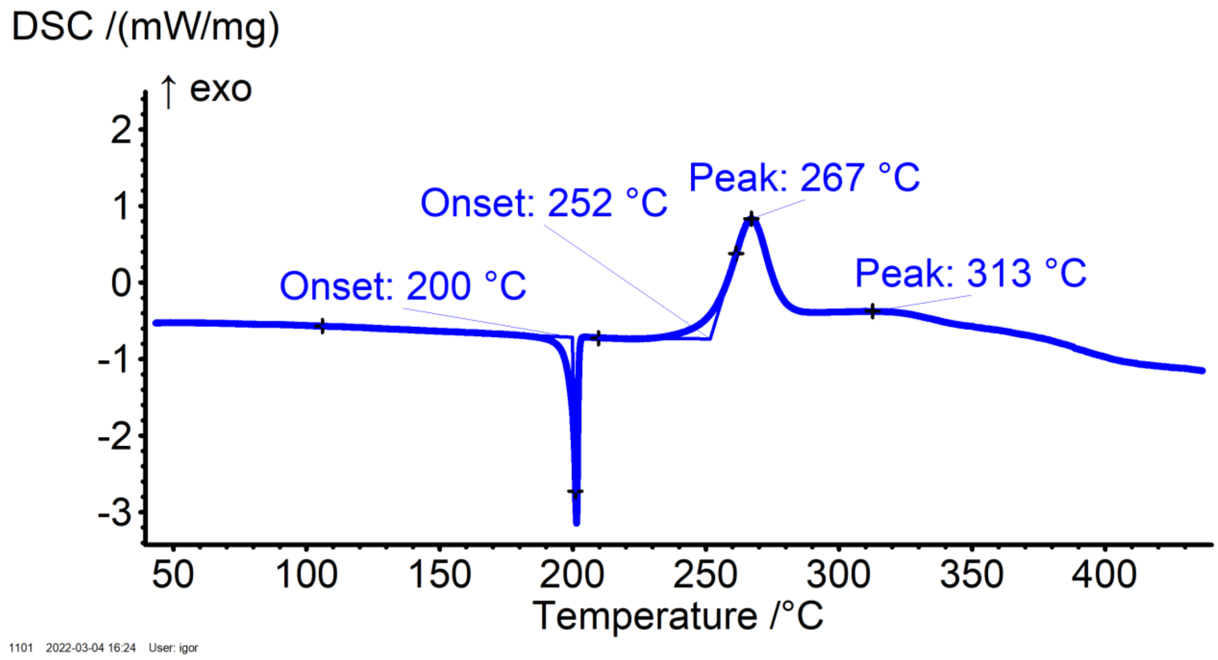

DSC curve for compound 5k

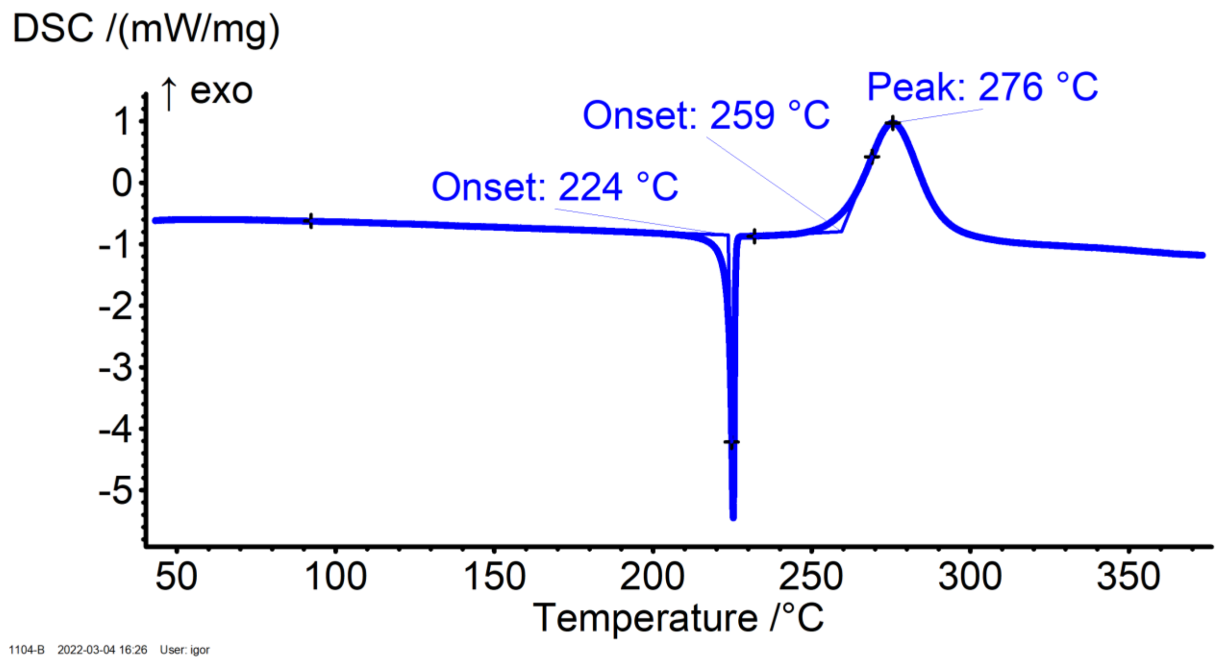

DSC curve for compound 6k

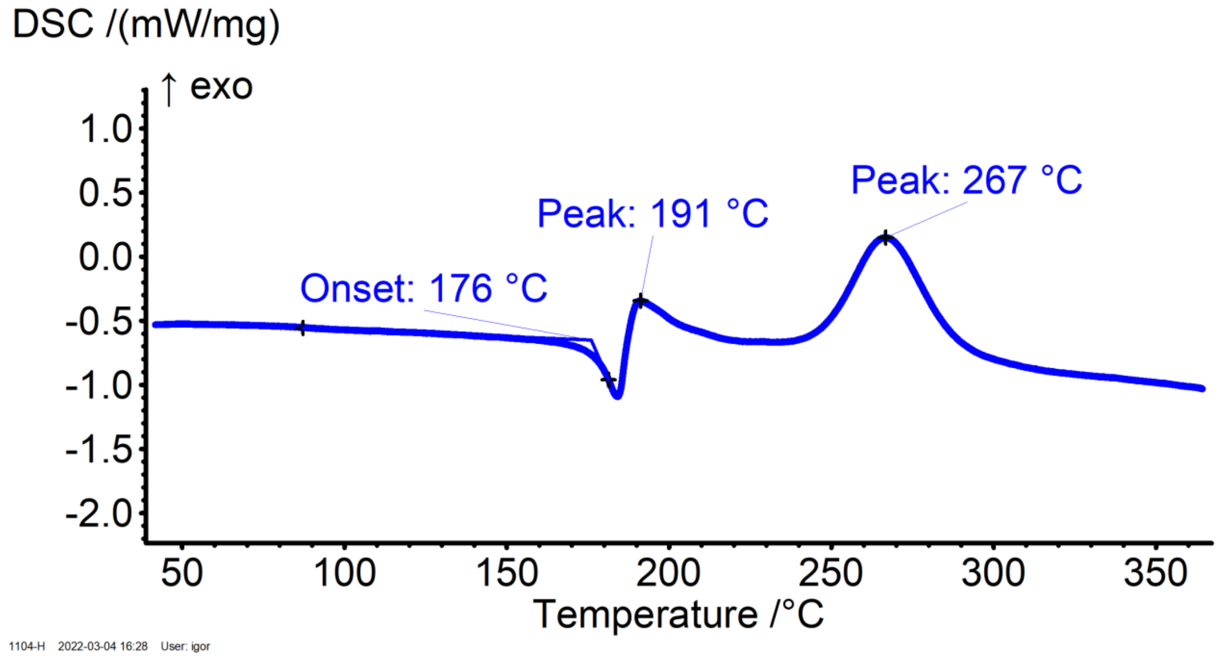

DSC curve for compound 6l

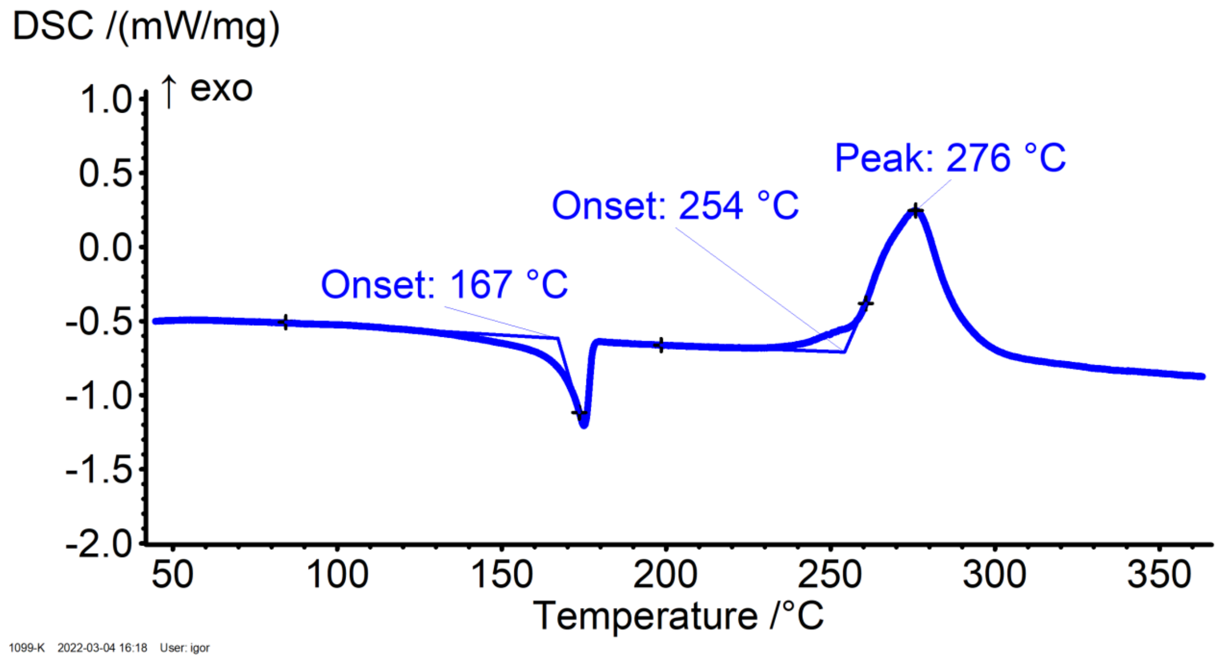

DSC curve for compound 7

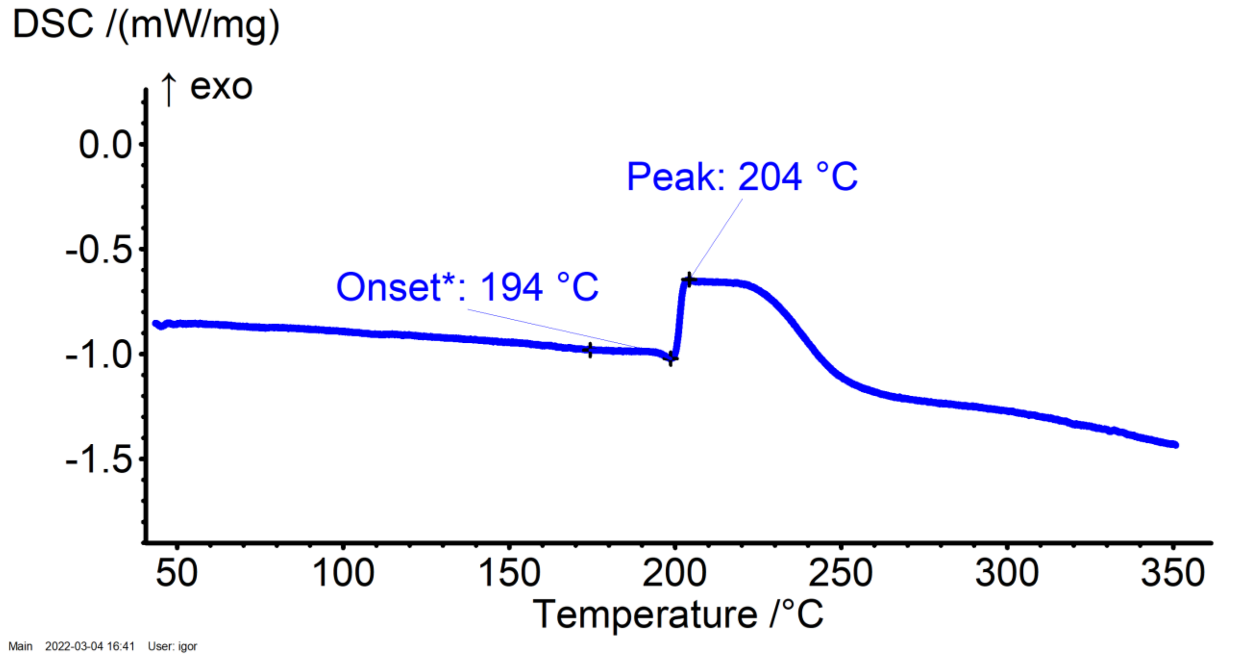

S2. Crystallographic data

Table S1. Crystal data and structure refinement for **7**.

Identification code	**7**	
Empirical formula	C32 H34 Cl4 N6 O3 S3	
Formula weight	788.63	
Temperature	100.00(10) K	
Wavelength	1.54184 Å	
Crystal system	Orthorhombic	
Space group	$P2_12_12_1$	
Unit cell dimensions	a = 9.19173(6) Å	α= 90°.
	b = 18.42596(16) Å	β= 90°.
	c = 41.9864(3) Å	γ = 90°.
Volume	7111.09(9) $Å^3$	
Z	8	
Density (calculated)	1.473 g/cm^3	
Absorption coefficient	5.031 mm^{-1}	
F(000)	3264	
Crystal size	0.132 x 0.111 x 0.032 mm^3	
Theta range for data collection	2.105 to 80.017°.	
Index ranges	-9<=h<=11, -23<=k<=23, -53<=l<=53	
Reflections collected	95880	
Independent reflections	15320 [R(int) = 0.0460]	
Observed reflections	14743	
Completeness to theta = 67.684°	100.0 %	
Absorption correction	Semi-empirical from equivalents	
Max. and min. transmission	1.00000 and 0.81599	
Refinement method	Full-matrix least-squares on F^2	
Data / restraints / parameters	15320 / 18 / 912	
Goodness-of-fit on F^2	1.056	
Final R indices [I>2sigma(I)]	R1 = 0.0373, wR2 = 0.0983	
R indices (all data)	R1 = 0.0386, wR2 = 0.0992	
Absolute structure parameter	0.007(3)	
Largest diff. peak and hole	0.327 and -0.426 e.$Å^{-3}$	

Table S2. Atomic coordinates (x 10^4) and equivalent isotropic displacement parameters ($Å^2$x 10^3) for **7**. U(eq) is defined as one third of the trace of the orthogonalized U^{ij} tensor.

	x	y	z	U(eq)
Cl(1B)	3423(1)	3960(1)	5209(1)	31(1)
Cl(3B)	12643(1)	6769(1)	7190(1)	35(1)
Cl(2A)	10669(1)	7033(1)	7972(1)	36(1)
S(50)	8908(1)	3559(1)	4788(1)	29(1)
Cl(1A)	-1246(1)	3923(1)	7371(1)	38(1)
Cl(3A)	7261(1)	6741(1)	5279(1)	40(1)
Cl(4B)	5052(1)	1904(1)	6898(1)	42(1)
Cl(4A)	182(1)	1895(1)	5679(1)	43(1)
Cl(2B)	15175(1)	6808(1)	4453(1)	45(1)
S(55)	4658(1)	8456(1)	7258(1)	35(1)
S(70)	8366(1)	4467(1)	4052(1)	45(1)
S(75)	4453(1)	7932(1)	5912(1)	49(1)
S(65)	10076(1)	8105(1)	6801(1)	41(1)
S(60)	8310(2)	10060(1)	6362(1)	44(1)
O(50)	10183(3)	3795(2)	4990(1)	35(1)
N(12A)	4523(3)	4880(2)	6912(1)	24(1)
N(12B)	9312(3)	4816(2)	5630(1)	25(1)
N(2A)	5429(3)	5523(2)	6518(1)	26(1)
N(2B)	10350(3)	5478(2)	6002(1)	25(1)
O(55)	4822(4)	9273(2)	7231(1)	36(1)
N(1A)	6156(3)	5753(2)	6797(1)	26(1)
N(14B)	10665(3)	5224(2)	5182(1)	28(1)
N(14A)	5934(3)	5383(2)	7339(1)	26(1)
N(4B)	8515(3)	4624(2)	6166(1)	25(1)
N(4A)	3681(3)	4607(2)	6387(1)	26(1)
O(75)	3315(3)	8292(2)	6114(1)	46(1)
N(5A)	2966(3)	4022(2)	6553(1)	25(1)
N(5B)	7677(3)	4060(2)	6019(1)	28(1)
N(1B)	11039(3)	5654(2)	5712(1)	26(1)
C(13A)	5570(3)	5371(2)	7027(1)	24(1)
O(70)	9789(4)	4699(2)	4196(1)	58(1)
C(21A)	5834(3)	5826(2)	6225(1)	24(1)
C(7A)	969(4)	3981(2)	6950(1)	27(1)
C(28A)	1943(4)	3720(2)	6030(1)	28(1)
C(27A)	2254(4)	3531(2)	6342(1)	25(1)

C(3A)	4480(3)	4972(2)	6582(1)	23(1)
C(3B)	9328(4)	4943(2)	5959(1)	24(1)
C(6A)	2342(4)	4202(2)	6850(1)	24(1)
C(9B)	6020(4)	4614(2)	5116(1)	28(1)
C(25B)	10755(4)	5940(2)	6854(1)	28(1)
O(65)	8741(4)	8458(2)	6931(1)	53(1)
C(26A)	5034(4)	5676(2)	5950(1)	25(1)
C(15A)	7058(3)	5806(2)	7475(1)	24(1)
C(11B)	7929(4)	4613(2)	5508(1)	25(1)
C(25A)	5472(4)	5968(2)	5661(1)	28(1)
C(23B)	12528(4)	6604(2)	6546(1)	29(1)
C(29B)	6467(4)	3172(2)	6767(1)	31(1)
C(26B)	10210(4)	5636(2)	6578(1)	26(1)
C(11A)	3181(3)	4655(2)	7050(1)	24(1)
C(31A)	1281(4)	2330(2)	6250(1)	33(1)
C(16A)	7702(4)	6401(2)	7328(1)	29(1)
C(23A)	7503(4)	6556(2)	5918(1)	29(1)
C(21B)	10841(3)	5805(2)	6284(1)	24(1)
C(24B)	11929(4)	6411(2)	6837(1)	27(1)
C(28B)	7064(4)	3684(2)	6562(1)	28(1)
C(27B)	7055(4)	3567(2)	6234(1)	26(1)
C(10A)	2647(4)	4884(2)	7342(1)	27(1)
C(13B)	10379(4)	5263(2)	5499(1)	25(1)
O(60)	9892(4)	10200(3)	6419(1)	60(1)
C(15B)	11751(4)	5618(2)	5023(1)	26(1)
C(9A)	1288(4)	4651(2)	7444(1)	30(1)
C(22A)	7059(4)	6277(2)	6209(1)	29(1)
C(30A)	967(4)	2531(2)	5938(1)	31(1)
C(6B)	7054(4)	4222(2)	5721(1)	26(1)
C(32A)	1930(4)	2830(2)	6450(1)	30(1)
C(19A)	8629(4)	5978(2)	7932(1)	32(1)
C(16B)	12368(4)	6254(2)	5142(1)	30(1)
C(10B)	7408(4)	4823(2)	5210(1)	27(1)
C(8B)	5156(4)	4221(2)	5327(1)	28(1)
C(17A)	8811(4)	6780(2)	7483(1)	31(1)
C(22B)	11983(4)	6308(2)	6269(1)	28(1)
C(24A)	6704(4)	6393(2)	5647(1)	28(1)
C(29A)	1285(4)	3217(2)	5826(1)	30(1)
C(32B)	6452(5)	2918(2)	6119(1)	36(1)
C(17B)	13425(4)	6612(2)	4967(1)	33(1)

C(20A)	7521(4)	5603(2)	7780(1)	30(1)
C(18A)	9267(4)	6569(2)	7781(1)	30(1)
C(30B)	5832(4)	2553(2)	6644(1)	32(1)
C(7B)	5643(4)	4043(2)	5630(1)	27(1)
C(19B)	13237(4)	5726(2)	4550(1)	34(1)
C(31B)	5809(5)	2426(2)	6320(1)	39(1)
C(20B)	12183(4)	5365(2)	4722(1)	31(1)
C(18B)	13856(4)	6349(2)	4675(1)	33(1)
C(8A)	475(4)	4203(2)	7248(1)	30(1)
C(51)	9664(5)	2943(2)	4502(1)	37(1)
C(52)	7939(4)	2909(2)	5024(1)	34(1)
C(56)	6132(6)	8133(3)	7496(1)	41(1)
C(57)	5286(6)	8089(3)	6888(1)	41(1)
C(71)	8388(6)	4782(3)	3650(1)	52(1)
C(67)	9769(6)	7979(3)	6382(1)	50(1)
C(72)	7006(6)	5077(3)	4194(1)	53(1)
C(61)	7448(8)	10082(4)	6748(2)	62(1)
C(76)	6138(6)	8345(3)	6015(1)	57(1)
C(66)	11420(6)	8772(3)	6760(1)	50(1)
C(77)	4343(6)	8345(4)	5523(1)	64(2)
C(62)	7608(7)	10886(4)	6204(2)	62(1)
C(61A)	7700(40)	10610(20)	6038(7)	62(1)
S(60A)	8351(12)	10918(8)	6408(3)	80(4)
C(62A)	7390(40)	10300(30)	6675(10)	62(1)
O(60A)	9870(20)	10640(20)	6421(7)	60(1)
O(55A)	4270(30)	9099(19)	7327(7)	36(1)
C(56A)	5080(60)	8290(30)	6816(9)	41(1)
S(55A)	5606(11)	8823(6)	7147(3)	35(1)
C(57A)	6290(60)	8100(20)	7400(10)	41(1)
O(65A)	8060(40)	8850(30)	6651(11)	53(1)
S(65A)	9387(17)	8713(9)	6438(4)	41(1)
C(66A)	9490(70)	7780(16)	6512(16)	50(1)
C(67A)	10850(50)	9020(40)	6690(15)	50(1)

Table S3. Bond lengths [Å] and angles [°] for **7**.

Cl(1B)-C(8B)	1.736(4)
Cl(3B)-C(24B)	1.750(4)
Cl(2A)-C(18A)	1.742(4)
S(50)-O(50)	1.511(3)
S(50)-C(51)	1.792(4)
S(50)-C(52)	1.793(4)
Cl(1A)-C(8A)	1.742(4)
Cl(3A)-C(24A)	1.749(4)
Cl(4B)-C(30B)	1.755(4)
Cl(4A)-C(30A)	1.755(4)
Cl(2B)-C(18B)	1.746(4)
S(55)-O(55)	1.518(3)
S(55)-C(56)	1.784(5)
S(55)-C(57)	1.793(5)
S(70)-O(70)	1.502(4)
S(70)-C(71)	1.787(5)
S(70)-C(72)	1.783(5)
S(75)-O(75)	1.502(3)
S(75)-C(76)	1.779(6)
S(75)-C(77)	1.802(6)
S(65)-O(65)	1.493(4)
S(65)-C(67)	1.799(5)
S(65)-C(66)	1.752(6)
S(60)-O(60)	1.496(4)
S(60)-C(61)	1.803(8)
S(60)-C(62)	1.781(7)
N(12A)-C(13A)	1.406(4)
N(12A)-C(3A)	1.397(4)
N(12A)-C(11A)	1.424(4)
N(12B)-C(3B)	1.399(4)
N(12B)-C(11B)	1.422(4)
N(12B)-C(13B)	1.395(4)
N(2A)-N(1A)	1.412(4)
N(2A)-C(21A)	1.404(4)
N(2A)-C(3A)	1.364(4)
N(2B)-N(1B)	1.411(4)
N(2B)-C(3B)	1.374(4)
N(2B)-C(21B)	1.403(4)

N(1A)-C(13A)	1.310(4)
N(14B)-C(13B)	1.355(4)
N(14B)-C(15B)	1.404(4)
N(14B)-H(14B)	0.84(5)
N(14A)-C(13A)	1.354(4)
N(14A)-C(15A)	1.415(4)
N(14A)-H(14A)	0.85(4)
N(4B)-N(5B)	1.432(4)
N(4B)-C(3B)	1.289(4)
N(4A)-N(5A)	1.441(4)
N(4A)-C(3A)	1.290(5)
N(5A)-C(27A)	1.424(4)
N(5A)-C(6A)	1.413(4)
N(5B)-C(27B)	1.401(5)
N(5B)-C(6B)	1.409(4)
N(1B)-C(13B)	1.300(5)
C(21A)-C(26A)	1.395(5)
C(21A)-C(22A)	1.400(5)
C(7A)-H(7A)	0.9500
C(7A)-C(6A)	1.391(5)
C(7A)-C(8A)	1.391(5)
C(28A)-H(28A)	0.9500
C(28A)-C(27A)	1.384(5)
C(28A)-C(29A)	1.399(5)
C(27A)-C(32A)	1.401(5)
C(6A)-C(11A)	1.413(5)
C(9B)-H(9B)	0.9500
C(9B)-C(10B)	1.391(5)
C(9B)-C(8B)	1.395(5)
C(25B)-H(25B)	0.9500
C(25B)-C(26B)	1.378(5)
C(25B)-C(24B)	1.388(5)
C(26A)-H(26A)	0.9500
C(26A)-C(25A)	1.386(5)
C(15A)-C(16A)	1.390(5)
C(15A)-C(20A)	1.398(5)
C(11B)-C(6B)	1.402(5)
C(11B)-C(10B)	1.391(5)
C(25A)-H(25A)	0.9500
C(25A)-C(24A)	1.379(5)

C(23B)-H(23B)	0.9500
C(23B)-C(24B)	1.385(5)
C(23B)-C(22B)	1.377(5)
C(29B)-H(29B)	0.9500
C(29B)-C(28B)	1.389(5)
C(29B)-C(30B)	1.381(5)
C(26B)-H(26B)	0.9500
C(26B)-C(21B)	1.399(5)
C(11A)-C(10A)	1.385(5)
C(31A)-H(31A)	0.9500
C(31A)-C(30A)	1.388(5)
C(31A)-C(32A)	1.382(5)
C(16A)-H(16A)	0.9500
C(16A)-C(17A)	1.396(5)
C(23A)-H(23A)	0.9500
C(23A)-C(22A)	1.388(5)
C(23A)-C(24A)	1.387(5)
C(21B)-C(22B)	1.401(5)
C(28B)-H(28B)	0.9500
C(28B)-C(27B)	1.394(5)
C(27B)-C(32B)	1.402(5)
C(10A)-H(10A)	0.9500
C(10A)-C(9A)	1.389(5)
C(15B)-C(16B)	1.393(5)
C(15B)-C(20B)	1.404(5)
C(9A)-H(9A)	0.9500
C(9A)-C(8A)	1.387(5)
C(22A)-H(22A)	0.9500
C(30A)-C(29A)	1.381(5)
C(6B)-C(7B)	1.392(5)
C(32A)-H(32A)	0.9500
C(19A)-H(19A)	0.9500
C(19A)-C(20A)	1.387(5)
C(19A)-C(18A)	1.390(5)
C(16B)-H(16B)	0.9500
C(16B)-C(17B)	1.386(5)
C(10B)-H(10B)	0.9500
C(8B)-C(7B)	1.389(5)
C(17A)-H(17A)	0.9500
C(17A)-C(18A)	1.374(5)

C(22B)-H(22B)	0.9500
C(29A)-H(29A)	0.9500
C(32B)-H(32B)	0.9500
C(32B)-C(31B)	1.372(6)
C(17B)-H(17B)	0.9500
C(17B)-C(18B)	1.379(6)
C(20A)-H(20A)	0.9500
C(30B)-C(31B)	1.380(6)
C(7B)-H(7B)	0.9500
C(19B)-H(19B)	0.9500
C(19B)-C(20B)	1.381(5)
C(19B)-C(18B)	1.385(6)
C(31B)-H(31B)	0.9500
C(20B)-H(20B)	0.9500
C(51)-H(51A)	0.9800
C(51)-H(51B)	0.9800
C(51)-H(51C)	0.9800
C(52)-H(52A)	0.9800
C(52)-H(52B)	0.9800
C(52)-H(52C)	0.9800
C(56)-H(56A)	0.9800
C(56)-H(56B)	0.9800
C(56)-H(56C)	0.9800
C(57)-H(57A)	0.9800
C(57)-H(57B)	0.9800
C(57)-H(57C)	0.9800
C(71)-H(71A)	0.9800
C(71)-H(71B)	0.9800
C(71)-H(71C)	0.9800
C(67)-H(67A)	0.9800
C(67)-H(67B)	0.9800
C(67)-H(67C)	0.9800
C(72)-H(72A)	0.9800
C(72)-H(72B)	0.9800
C(72)-H(72C)	0.9800
C(61)-H(61A)	0.9800
C(61)-H(61B)	0.9800
C(61)-H(61C)	0.9800
C(76)-H(76A)	0.9800
C(76)-H(76B)	0.9800

C(76)-H(76C)	0.9800
C(66)-H(66A)	0.9800
C(66)-H(66B)	0.9800
C(66)-H(66C)	0.9800
C(77)-H(77A)	0.9800
C(77)-H(77B)	0.9800
C(77)-H(77C)	0.9800
C(62)-H(62A)	0.9800
C(62)-H(62B)	0.9800
C(62)-H(62C)	0.9800
C(61A)-H(61D)	0.9800
C(61A)-H(61E)	0.9800
C(61A)-H(61F)	0.9800
C(61A)-S(60A)	1.76(2)
S(60A)-C(62A)	1.83(2)
S(60A)-O(60A)	1.48(2)
C(62A)-H(62D)	0.9800
C(62A)-H(62E)	0.9800
C(62A)-H(62F)	0.9800
O(55A)-S(55A)	1.53(2)
C(56A)-H(56D)	0.9800
C(56A)-H(56E)	0.9800
C(56A)-H(56F)	0.9800
C(56A)-S(55A)	1.77(2)
S(55A)-C(57A)	1.81(3)
C(57A)-H(57D)	0.9800
C(57A)-H(57E)	0.9800
C(57A)-H(57F)	0.9800
O(65A)-S(65A)	1.53(2)
S(65A)-C(66A)	1.75(3)
S(65A)-C(67A)	1.80(3)
C(66A)-H(66D)	0.9800
C(66A)-H(66E)	0.9800
C(66A)-H(66F)	0.9800
C(67A)-H(67D)	0.9800
C(67A)-H(67E)	0.9800
C(67A)-H(67F)	0.9800

O(50)-S(50)-C(51)	104.92(18)
O(50)-S(50)-C(52)	105.50(17)

C(52)-S(50)-C(51)	98.05(19)
O(55)-S(55)-C(56)	107.3(2)
O(55)-S(55)-C(57)	106.0(2)
C(56)-S(55)-C(57)	96.7(2)
O(70)-S(70)-C(71)	106.1(2)
O(70)-S(70)-C(72)	107.3(2)
C(72)-S(70)-C(71)	96.8(2)
O(75)-S(75)-C(76)	106.3(2)
O(75)-S(75)-C(77)	106.7(2)
C(76)-S(75)-C(77)	95.1(3)
O(65)-S(65)-C(67)	106.6(2)
O(65)-S(65)-C(66)	108.1(2)
C(66)-S(65)-C(67)	96.0(3)
O(60)-S(60)-C(61)	106.3(3)
O(60)-S(60)-C(62)	105.3(3)
C(62)-S(60)-C(61)	99.0(3)
C(13A)-N(12A)-C(11A)	130.0(3)
C(3A)-N(12A)-C(13A)	106.2(3)
C(3A)-N(12A)-C(11A)	114.4(3)
C(3B)-N(12B)-C(11B)	114.3(3)
C(13B)-N(12B)-C(3B)	106.5(3)
C(13B)-N(12B)-C(11B)	129.8(3)
C(21A)-N(2A)-N(1A)	118.8(3)
C(3A)-N(2A)-N(1A)	111.3(3)
C(3A)-N(2A)-C(21A)	129.7(3)
C(3B)-N(2B)-N(1B)	110.9(3)
C(3B)-N(2B)-C(21B)	129.8(3)
C(21B)-N(2B)-N(1B)	119.1(3)
C(13A)-N(1A)-N(2A)	104.7(3)
C(13B)-N(14B)-C(15B)	125.2(3)
C(13B)-N(14B)-H(14B)	112(3)
C(15B)-N(14B)-H(14B)	114(3)
C(13A)-N(14A)-C(15A)	125.5(3)
C(13A)-N(14A)-H(14A)	119(3)
C(15A)-N(14A)-H(14A)	112(3)
C(3B)-N(4B)-N(5B)	110.6(3)
C(3A)-N(4A)-N(5A)	110.2(3)
C(27A)-N(5A)-N(4A)	112.7(3)
C(6A)-N(5A)-N(4A)	115.7(3)
C(6A)-N(5A)-C(27A)	120.8(3)

C(27B)-N(5B)-N(4B)	114.4(3)
C(27B)-N(5B)-C(6B)	122.9(3)
C(6B)-N(5B)-N(4B)	116.6(3)
C(13B)-N(1B)-N(2B)	105.0(3)
N(1A)-C(13A)-N(12A)	112.1(3)
N(1A)-C(13A)-N(14A)	127.1(3)
N(14A)-C(13A)-N(12A)	120.6(3)
C(26A)-C(21A)-N(2A)	120.5(3)
C(26A)-C(21A)-C(22A)	120.2(3)
C(22A)-C(21A)-N(2A)	119.3(3)
C(6A)-C(7A)-H(7A)	120.6
C(6A)-C(7A)-C(8A)	118.7(3)
C(8A)-C(7A)-H(7A)	120.6
C(27A)-C(28A)-H(28A)	119.9
C(27A)-C(28A)-C(29A)	120.2(3)
C(29A)-C(28A)-H(28A)	119.9
C(28A)-C(27A)-N(5A)	121.6(3)
C(28A)-C(27A)-C(32A)	119.5(3)
C(32A)-C(27A)-N(5A)	118.8(3)
N(2A)-C(3A)-N(12A)	105.6(3)
N(4A)-C(3A)-N(12A)	125.6(3)
N(4A)-C(3A)-N(2A)	128.8(3)
N(2B)-C(3B)-N(12B)	105.0(3)
N(4B)-C(3B)-N(12B)	125.6(3)
N(4B)-C(3B)-N(2B)	129.4(3)
C(7A)-C(6A)-N(5A)	124.4(3)
C(7A)-C(6A)-C(11A)	119.3(3)
C(11A)-C(6A)-N(5A)	116.3(3)
C(10B)-C(9B)-H(9B)	120.5
C(10B)-C(9B)-C(8B)	118.9(3)
C(8B)-C(9B)-H(9B)	120.5
C(26B)-C(25B)-H(25B)	120.2
C(26B)-C(25B)-C(24B)	119.6(3)
C(24B)-C(25B)-H(25B)	120.2
C(21A)-C(26A)-H(26A)	120.2
C(25A)-C(26A)-C(21A)	119.6(3)
C(25A)-C(26A)-H(26A)	120.2
C(16A)-C(15A)-N(14A)	124.5(3)
C(16A)-C(15A)-C(20A)	119.2(3)
C(20A)-C(15A)-N(14A)	116.4(3)

C(6B)-C(11B)-N(12B)	114.6(3)
C(10B)-C(11B)-N(12B)	124.1(3)
C(10B)-C(11B)-C(6B)	121.2(3)
C(26A)-C(25A)-H(25A)	120.1
C(24A)-C(25A)-C(26A)	119.8(3)
C(24A)-C(25A)-H(25A)	120.1
C(24B)-C(23B)-H(23B)	120.1
C(22B)-C(23B)-H(23B)	120.1
C(22B)-C(23B)-C(24B)	119.8(3)
C(28B)-C(29B)-H(29B)	120.1
C(30B)-C(29B)-H(29B)	120.1
C(30B)-C(29B)-C(28B)	119.8(3)
C(25B)-C(26B)-H(26B)	120.0
C(25B)-C(26B)-C(21B)	119.9(3)
C(21B)-C(26B)-H(26B)	120.0
C(6A)-C(11A)-N(12A)	113.8(3)
C(10A)-C(11A)-N(12A)	125.2(3)
C(10A)-C(11A)-C(6A)	120.8(3)
C(30A)-C(31A)-H(31A)	120.5
C(32A)-C(31A)-H(31A)	120.5
C(32A)-C(31A)-C(30A)	119.0(4)
C(15A)-C(16A)-H(16A)	120.1
C(15A)-C(16A)-C(17A)	119.9(3)
C(17A)-C(16A)-H(16A)	120.1
C(22A)-C(23A)-H(23A)	120.4
C(24A)-C(23A)-H(23A)	120.4
C(24A)-C(23A)-C(22A)	119.1(3)
C(26B)-C(21B)-N(2B)	121.0(3)
C(26B)-C(21B)-C(22B)	119.8(3)
C(22B)-C(21B)-N(2B)	119.1(3)
C(25B)-C(24B)-Cl(3B)	119.0(3)
C(23B)-C(24B)-Cl(3B)	120.1(3)
C(23B)-C(24B)-C(25B)	120.9(3)
C(29B)-C(28B)-H(28B)	119.9
C(29B)-C(28B)-C(27B)	120.3(3)
C(27B)-C(28B)-H(28B)	119.9
N(5B)-C(27B)-C(32B)	119.6(3)
C(28B)-C(27B)-N(5B)	122.1(3)
C(28B)-C(27B)-C(32B)	118.2(3)
C(11A)-C(10A)-H(10A)	120.0

C(11A)-C(10A)-C(9A)	120.0(3)
C(9A)-C(10A)-H(10A)	120.0
N(14B)-C(13B)-N(12B)	119.6(3)
N(1B)-C(13B)-N(12B)	112.5(3)
N(1B)-C(13B)-N(14B)	127.8(3)
N(14B)-C(15B)-C(20B)	117.2(3)
C(16B)-C(15B)-N(14B)	123.6(3)
C(16B)-C(15B)-C(20B)	119.2(3)
C(10A)-C(9A)-H(9A)	120.6
C(8A)-C(9A)-C(10A)	118.9(3)
C(8A)-C(9A)-H(9A)	120.6
C(21A)-C(22A)-H(22A)	120.1
C(23A)-C(22A)-C(21A)	119.8(3)
C(23A)-C(22A)-H(22A)	120.1
C(31A)-C(30A)-Cl(4A)	119.5(3)
C(29A)-C(30A)-Cl(4A)	119.0(3)
C(29A)-C(30A)-C(31A)	121.5(4)
C(11B)-C(6B)-N(5B)	116.3(3)
C(7B)-C(6B)-N(5B)	124.8(3)
C(7B)-C(6B)-C(11B)	118.8(3)
C(27A)-C(32A)-H(32A)	119.7
C(31A)-C(32A)-C(27A)	120.6(3)
C(31A)-C(32A)-H(32A)	119.7
C(20A)-C(19A)-H(19A)	120.4
C(20A)-C(19A)-C(18A)	119.2(3)
C(18A)-C(19A)-H(19A)	120.4
C(15B)-C(16B)-H(16B)	120.1
C(17B)-C(16B)-C(15B)	119.8(3)
C(17B)-C(16B)-H(16B)	120.1
C(9B)-C(10B)-C(11B)	119.7(3)
C(9B)-C(10B)-H(10B)	120.1
C(11B)-C(10B)-H(10B)	120.1
C(9B)-C(8B)-Cl(1B)	119.0(3)
C(7B)-C(8B)-Cl(1B)	119.5(3)
C(7B)-C(8B)-C(9B)	121.5(3)
C(16A)-C(17A)-H(17A)	119.9
C(18A)-C(17A)-C(16A)	120.3(3)
C(18A)-C(17A)-H(17A)	119.9
C(23B)-C(22B)-C(21B)	119.7(3)
C(23B)-C(22B)-H(22B)	120.1

C(21B)-C(22B)-H(22B)	120.1
C(25A)-C(24A)-Cl(3A)	119.2(3)
C(25A)-C(24A)-C(23A)	121.5(3)
C(23A)-C(24A)-Cl(3A)	119.3(3)
C(28A)-C(29A)-H(29A)	120.4
C(30A)-C(29A)-C(28A)	119.2(3)
C(30A)-C(29A)-H(29A)	120.4
C(27B)-C(32B)-H(32B)	119.2
C(31B)-C(32B)-C(27B)	121.5(4)
C(31B)-C(32B)-H(32B)	119.2
C(16B)-C(17B)-H(17B)	119.8
C(18B)-C(17B)-C(16B)	120.4(4)
C(18B)-C(17B)-H(17B)	119.8
C(15A)-C(20A)-H(20A)	119.6
C(19A)-C(20A)-C(15A)	120.8(3)
C(19A)-C(20A)-H(20A)	119.6
C(19A)-C(18A)-Cl(2A)	119.1(3)
C(17A)-C(18A)-Cl(2A)	120.3(3)
C(17A)-C(18A)-C(19A)	120.6(3)
C(29B)-C(30B)-Cl(4B)	120.5(3)
C(31B)-C(30B)-Cl(4B)	118.5(3)
C(31B)-C(30B)-C(29B)	120.9(4)
C(6B)-C(7B)-H(7B)	120.2
C(8B)-C(7B)-C(6B)	119.7(3)
C(8B)-C(7B)-H(7B)	120.2
C(20B)-C(19B)-H(19B)	120.4
C(20B)-C(19B)-C(18B)	119.2(4)
C(18B)-C(19B)-H(19B)	120.4
C(32B)-C(31B)-C(30B)	119.1(4)
C(32B)-C(31B)-H(31B)	120.4
C(30B)-C(31B)-H(31B)	120.4
C(15B)-C(20B)-H(20B)	119.7
C(19B)-C(20B)-C(15B)	120.7(4)
C(19B)-C(20B)-H(20B)	119.7
C(17B)-C(18B)-Cl(2B)	120.2(3)
C(17B)-C(18B)-C(19B)	120.8(4)
C(19B)-C(18B)-Cl(2B)	119.0(3)
C(7A)-C(8A)-Cl(1A)	118.4(3)
C(9A)-C(8A)-Cl(1A)	119.2(3)
C(9A)-C(8A)-C(7A)	122.3(3)

S(50)-C(51)-H(51A)	109.5
S(50)-C(51)-H(51B)	109.5
S(50)-C(51)-H(51C)	109.5
H(51A)-C(51)-H(51B)	109.5
H(51A)-C(51)-H(51C)	109.5
H(51B)-C(51)-H(51C)	109.5
S(50)-C(52)-H(52A)	109.5
S(50)-C(52)-H(52B)	109.5
S(50)-C(52)-H(52C)	109.5
H(52A)-C(52)-H(52B)	109.5
H(52A)-C(52)-H(52C)	109.5
H(52B)-C(52)-H(52C)	109.5
S(55)-C(56)-H(56A)	109.5
S(55)-C(56)-H(56B)	109.5
S(55)-C(56)-H(56C)	109.5
H(56A)-C(56)-H(56B)	109.5
H(56A)-C(56)-H(56C)	109.5
H(56B)-C(56)-H(56C)	109.5
S(55)-C(57)-H(57A)	109.5
S(55)-C(57)-H(57B)	109.5
S(55)-C(57)-H(57C)	109.5
H(57A)-C(57)-H(57B)	109.5
H(57A)-C(57)-H(57C)	109.5
H(57B)-C(57)-H(57C)	109.5
S(70)-C(71)-H(71A)	109.5
S(70)-C(71)-H(71B)	109.5
S(70)-C(71)-H(71C)	109.5
H(71A)-C(71)-H(71B)	109.5
H(71A)-C(71)-H(71C)	109.5
H(71B)-C(71)-H(71C)	109.5
S(65)-C(67)-H(67A)	109.5
S(65)-C(67)-H(67B)	109.5
S(65)-C(67)-H(67C)	109.5
H(67A)-C(67)-H(67B)	109.5
H(67A)-C(67)-H(67C)	109.5
H(67B)-C(67)-H(67C)	109.5
S(70)-C(72)-H(72A)	109.5
S(70)-C(72)-H(72B)	109.5
S(70)-C(72)-H(72C)	109.5
H(72A)-C(72)-H(72B)	109.5

H(72A)-C(72)-H(72C)	109.5
H(72B)-C(72)-H(72C)	109.5
S(60)-C(61)-H(61A)	109.5
S(60)-C(61)-H(61B)	109.5
S(60)-C(61)-H(61C)	109.5
H(61A)-C(61)-H(61B)	109.5
H(61A)-C(61)-H(61C)	109.5
H(61B)-C(61)-H(61C)	109.5
S(75)-C(76)-H(76A)	109.5
S(75)-C(76)-H(76B)	109.5
S(75)-C(76)-H(76C)	109.5
H(76A)-C(76)-H(76B)	109.5
H(76A)-C(76)-H(76C)	109.5
H(76B)-C(76)-H(76C)	109.5
S(65)-C(66)-H(66A)	109.5
S(65)-C(66)-H(66B)	109.5
S(65)-C(66)-H(66C)	109.5
H(66A)-C(66)-H(66B)	109.5
H(66A)-C(66)-H(66C)	109.5
H(66B)-C(66)-H(66C)	109.5
S(75)-C(77)-H(77A)	109.5
S(75)-C(77)-H(77B)	109.5
S(75)-C(77)-H(77C)	109.5
H(77A)-C(77)-H(77B)	109.5
H(77A)-C(77)-H(77C)	109.5
H(77B)-C(77)-H(77C)	109.5
S(60)-C(62)-H(62A)	109.5
S(60)-C(62)-H(62B)	109.5
S(60)-C(62)-H(62C)	109.5
H(62A)-C(62)-H(62B)	109.5
H(62A)-C(62)-H(62C)	109.5
H(62B)-C(62)-H(62C)	109.5
H(61D)-C(61A)-H(61E)	109.5
H(61D)-C(61A)-H(61F)	109.5
H(61E)-C(61A)-H(61F)	109.5
S(60A)-C(61A)-H(61D)	109.5
S(60A)-C(61A)-H(61E)	109.5
S(60A)-C(61A)-H(61F)	109.5
C(61A)-S(60A)-C(62A)	100.1(19)
O(60A)-S(60A)-C(61A)	104.0(18)

O(60A)-S(60A)-C(62A)	102.6(19)
S(60A)-C(62A)-H(62D)	109.5
S(60A)-C(62A)-H(62E)	109.5
S(60A)-C(62A)-H(62F)	109.5
H(62D)-C(62A)-H(62E)	109.5
H(62D)-C(62A)-H(62F)	109.5
H(62E)-C(62A)-H(62F)	109.5
H(56D)-C(56A)-H(56E)	109.5
H(56D)-C(56A)-H(56F)	109.5
H(56E)-C(56A)-H(56F)	109.5
S(55A)-C(56A)-H(56D)	109.5
S(55A)-C(56A)-H(56E)	109.5
S(55A)-C(56A)-H(56F)	109.5
O(55A)-S(55A)-C(56A)	111(2)
O(55A)-S(55A)-C(57A)	103(2)
C(56A)-S(55A)-C(57A)	99(2)
S(55A)-C(57A)-H(57D)	109.5
S(55A)-C(57A)-H(57E)	109.5
S(55A)-C(57A)-H(57F)	109.5
H(57D)-C(57A)-H(57E)	109.5
H(57D)-C(57A)-H(57F)	109.5
H(57E)-C(57A)-H(57F)	109.5
O(65A)-S(65A)-C(66A)	96(2)
O(65A)-S(65A)-C(67A)	102(2)
C(66A)-S(65A)-C(67A)	100(2)
S(65A)-C(66A)-H(66D)	109.5
S(65A)-C(66A)-H(66E)	109.5
S(65A)-C(66A)-H(66F)	109.5
H(66D)-C(66A)-H(66E)	109.5
H(66D)-C(66A)-H(66F)	109.5
H(66E)-C(66A)-H(66F)	109.5
S(65A)-C(67A)-H(67D)	109.5
S(65A)-C(67A)-H(67E)	109.5
S(65A)-C(67A)-H(67F)	109.5
H(67D)-C(67A)-H(67E)	109.5
H(67D)-C(67A)-H(67F)	109.5
H(67E)-C(67A)-H(67F)	109.5

Table S4. Anisotropic displacement parameters ($Å^2$x 10^3) for **7**. The anisotropic displacement factor exponent takes the form: $-2\pi^2[h^2 a^{*2}U^{11} + ... + 2 h k a^* b^* U^{12}]$

	U^{11}	U^{22}	U^{33}	U^{23}	U^{13}	U^{12}
Cl(1B)	23(1)	36(1)	36(1)	1(1)	-4(1)	-3(1)
Cl(3B)	36(1)	39(1)	30(1)	-7(1)	-3(1)	-5(1)
Cl(2A)	31(1)	43(1)	34(1)	-7(1)	0(1)	-10(1)
S(50)	28(1)	32(1)	29(1)	-5(1)	0(1)	2(1)
Cl(1A)	24(1)	55(1)	36(1)	-9(1)	9(1)	-9(1)
Cl(3A)	48(1)	44(1)	28(1)	9(1)	5(1)	-8(1)
Cl(4B)	46(1)	41(1)	40(1)	8(1)	2(1)	-10(1)
Cl(4A)	52(1)	42(1)	34(1)	-2(1)	-5(1)	-14(1)
Cl(2B)	36(1)	59(1)	40(1)	14(1)	6(1)	-7(1)
S(55)	27(1)	39(1)	40(1)	-12(1)	5(1)	-3(1)
S(70)	48(1)	45(1)	42(1)	1(1)	-6(1)	1(1)
S(75)	45(1)	39(1)	62(1)	-4(1)	22(1)	-3(1)
S(65)	44(1)	42(1)	37(1)	3(1)	3(1)	6(1)
S(60)	44(1)	46(1)	43(1)	-6(1)	-8(1)	6(1)
O(50)	29(1)	41(1)	36(1)	-7(1)	-5(1)	-1(1)
N(12A)	18(1)	33(2)	22(1)	3(1)	-1(1)	-2(1)
N(12B)	19(1)	30(1)	25(1)	-3(1)	2(1)	-2(1)
N(2A)	21(1)	36(2)	21(1)	2(1)	-1(1)	-3(1)
N(2B)	22(1)	30(1)	23(1)	-4(1)	3(1)	-3(1)
O(55)	36(2)	40(2)	33(2)	-10(1)	-1(1)	5(1)
N(1A)	21(1)	35(2)	24(1)	1(1)	-3(1)	-1(1)
N(14B)	22(1)	34(2)	27(1)	-6(1)	3(1)	-4(1)
N(14A)	21(1)	33(2)	24(1)	4(1)	-3(1)	-4(1)
N(4B)	21(1)	28(1)	25(1)	-5(1)	1(1)	-5(1)
N(4A)	23(1)	32(2)	23(1)	3(1)	3(1)	-4(1)
O(75)	41(2)	55(2)	42(2)	2(1)	13(1)	4(1)
N(5A)	23(1)	30(1)	22(1)	1(1)	3(1)	-5(1)
N(5B)	30(1)	28(1)	25(1)	-2(1)	-3(1)	-4(1)
N(1B)	21(1)	33(2)	25(1)	-2(1)	4(1)	-1(1)
C(13A)	18(1)	29(2)	24(2)	4(1)	-1(1)	-1(1)
O(70)	51(2)	74(2)	50(2)	2(2)	-14(2)	1(2)
C(21A)	20(1)	27(2)	25(2)	6(1)	2(1)	3(1)
C(7A)	23(2)	33(2)	24(2)	1(1)	1(1)	1(1)
C(28A)	23(2)	34(2)	26(2)	2(1)	2(1)	1(1)
C(27A)	19(1)	32(2)	25(2)	-1(1)	2(1)	2(1)

C(3A)	17(1)	29(2)	25(2)	2(1)	1(1)	0(1)
C(3B)	21(2)	28(2)	24(2)	-5(1)	0(1)	0(1)
C(6A)	21(1)	28(2)	24(2)	3(1)	0(1)	1(1)
C(9B)	23(2)	35(2)	26(2)	-1(1)	-3(1)	0(1)
C(25B)	26(2)	30(2)	28(2)	1(1)	-1(1)	2(1)
O(65)	40(2)	65(2)	54(2)	-4(2)	13(2)	-5(2)
C(26A)	21(2)	30(2)	25(2)	0(1)	2(1)	2(1)
C(15A)	19(1)	27(2)	27(2)	-2(1)	1(1)	2(1)
C(11B)	21(2)	29(2)	24(2)	-6(1)	-1(1)	1(1)
C(25A)	28(2)	30(2)	24(2)	0(1)	1(1)	1(1)
C(23B)	23(2)	31(2)	34(2)	-7(1)	2(1)	-4(1)
C(29B)	25(2)	39(2)	30(2)	0(2)	1(1)	-4(2)
C(26B)	21(2)	29(2)	28(2)	-1(1)	1(1)	2(1)
C(11A)	18(1)	31(2)	24(2)	4(1)	2(1)	-1(1)
C(31A)	35(2)	34(2)	28(2)	3(1)	3(2)	-4(2)
C(16A)	27(2)	32(2)	29(2)	4(1)	-1(1)	0(1)
C(23A)	23(2)	32(2)	33(2)	6(1)	3(1)	2(1)
C(21B)	19(1)	26(2)	27(2)	-5(1)	-1(1)	3(1)
C(24B)	22(2)	30(2)	30(2)	-3(1)	-2(1)	1(1)
C(28B)	22(2)	33(2)	29(2)	-4(1)	-2(1)	-2(1)
C(27B)	22(2)	28(2)	28(2)	1(1)	0(1)	0(1)
C(10A)	24(2)	32(2)	26(2)	1(1)	1(1)	0(1)
C(13B)	21(2)	30(2)	24(2)	-2(1)	4(1)	0(1)
O(60)	38(2)	84(3)	57(2)	-6(3)	0(2)	12(2)
C(15B)	20(1)	34(2)	24(2)	1(1)	1(1)	2(1)
C(9A)	24(2)	38(2)	28(2)	0(1)	4(1)	2(1)
C(22A)	23(2)	35(2)	29(2)	5(1)	-1(1)	-1(1)
C(30A)	27(2)	38(2)	29(2)	-1(2)	2(1)	-6(2)
C(6B)	28(2)	28(2)	23(2)	-5(1)	0(1)	-2(1)
C(32A)	31(2)	32(2)	26(2)	2(1)	1(1)	-1(1)
C(19A)	32(2)	37(2)	28(2)	-1(1)	-4(1)	-2(2)
C(16B)	29(2)	34(2)	26(2)	1(1)	-2(1)	1(1)
C(10B)	23(2)	30(2)	27(2)	1(1)	0(1)	-1(1)
C(8B)	24(2)	31(2)	29(2)	-2(1)	2(1)	2(1)
C(17A)	28(2)	33(2)	33(2)	0(2)	4(1)	-4(1)
C(22B)	22(2)	30(2)	30(2)	-4(1)	4(1)	0(1)
C(24A)	27(2)	31(2)	28(2)	4(1)	1(1)	4(1)
C(29A)	25(2)	37(2)	28(2)	1(1)	-1(1)	0(1)
C(32B)	47(2)	32(2)	30(2)	-3(2)	-1(2)	-6(2)
C(17B)	32(2)	38(2)	29(2)	7(2)	-5(2)	-4(2)

C(20A)	30(2)	34(2)	28(2)	3(1)	-3(1)	-4(1)
C(18A)	24(2)	35(2)	31(2)	-3(1)	3(1)	2(1)
C(30B)	26(2)	35(2)	34(2)	5(2)	-1(1)	-1(2)
C(7B)	26(2)	28(2)	27(2)	-2(1)	1(1)	-2(1)
C(19B)	31(2)	43(2)	28(2)	2(2)	2(1)	5(2)
C(31B)	49(2)	27(2)	40(2)	-1(2)	-3(2)	-7(2)
C(20B)	29(2)	35(2)	29(2)	-1(1)	3(1)	2(2)
C(18B)	28(2)	41(2)	30(2)	10(2)	-1(1)	3(2)
C(8A)	26(2)	38(2)	27(2)	2(1)	4(1)	0(1)
C(51)	38(2)	40(2)	34(2)	-8(2)	2(2)	2(2)
C(52)	31(2)	36(2)	34(2)	-5(2)	1(2)	2(2)
C(56)	40(2)	42(2)	41(2)	-9(2)	4(2)	-1(1)
C(57)	40(2)	42(2)	41(2)	-9(2)	4(2)	-1(1)
C(71)	47(2)	70(3)	40(2)	-8(2)	-2(2)	10(2)
C(67)	42(2)	66(2)	43(2)	-13(2)	-3(2)	3(2)
C(72)	52(3)	65(3)	40(2)	-3(2)	5(2)	1(2)
C(61)	47(2)	73(3)	65(3)	17(2)	-4(2)	4(2)
C(76)	45(3)	78(4)	48(3)	9(2)	7(2)	8(3)
C(66)	42(2)	66(2)	43(2)	-13(2)	-3(2)	3(2)
C(77)	47(3)	108(5)	37(2)	-21(3)	8(2)	-9(3)
C(62)	47(2)	73(3)	65(3)	17(2)	-4(2)	4(2)
C(61A)	47(2)	73(3)	65(3)	17(2)	-4(2)	4(2)
S(60A)	45(5)	102(10)	92(9)	-12(7)	-1(6)	22(6)
C(62A)	47(2)	73(3)	65(3)	17(2)	-4(2)	4(2)
O(60A)	38(2)	84(3)	57(2)	-6(3)	0(2)	12(2)
O(55A)	36(2)	40(2)	33(2)	-10(1)	-1(1)	5(1)
C(56A)	40(2)	42(2)	41(2)	-9(2)	4(2)	-1(1)
S(55A)	27(1)	39(1)	40(1)	-12(1)	5(1)	-3(1)
C(57A)	40(2)	42(2)	41(2)	-9(2)	4(2)	-1(1)
O(65A)	40(2)	65(2)	54(2)	-4(2)	13(2)	-5(2)
S(65A)	44(1)	42(1)	37(1)	3(1)	3(1)	6(1)
C(66A)	42(2)	66(2)	43(2)	-13(2)	-3(2)	3(2)
C(67A)	42(2)	66(2)	43(2)	-13(2)	-3(2)	3(2)

Table S5. Hydrogen coordinates (x 10^4) and isotropic displacement parameters ($Å^2$x 10^3) for **7**.

	x	y	z	U(eq)
H(7A)	379	3685	6817	32
H(28A)	2177	4191	5954	33
H(9B)	5667	4737	4910	34
H(25B)	10329	5827	7054	34
H(26A)	4196	5376	5961	30
H(25A)	4924	5875	5474	33
H(23B)	13312	6939	6537	35
H(29B)	6494	3248	6990	38
H(26B)	9406	5313	6588	31
H(31A)	1054	1857	6324	39
H(16A)	7389	6549	7123	35
H(23A)	8342	6855	5905	35
H(28B)	7481	4115	6645	34
H(10A)	3209	5200	7471	33
H(9A)	922	4795	7646	36
H(22A)	7585	6390	6398	35
H(32A)	2158	2697	6663	36
H(19A)	8949	5832	8138	39
H(16B)	12065	6440	5342	36
H(10B)	7998	5108	5073	32
H(17A)	9251	7186	7383	37
H(22B)	12379	6444	6069	33
H(29A)	1060	3346	5612	36
H(32B)	6489	2817	5897	44
H(17B)	13856	7042	5049	40
H(20A)	7071	5204	7883	36
H(7B)	5016	3800	5775	32
H(19B)	13535	5549	4347	41
H(31B)	5353	2004	6237	46
H(20B)	11746	4940	4637	37
H(51A)	10333	3206	4362	56
H(51B)	8878	2730	4375	56
H(51C)	10192	2557	4613	56
H(52A)	8617	2539	5102	51

H(52B)	7185	2678	4894	51
H(52C)	7484	3154	5206	51
H(56A)	7055	8292	7402	61
H(56B)	6106	7602	7504	61
H(56C)	6044	8329	7712	61
H(57A)	4672	8269	6714	61
H(57B)	5234	7558	6895	61
H(57C)	6295	8240	6851	61
H(71A)	8649	5298	3646	78
H(71B)	7422	4717	3555	78
H(71C)	9105	4504	3527	78
H(67A)	8994	7621	6350	76
H(67B)	10665	7806	6281	76
H(67C)	9479	8442	6286	76
H(72A)	6900	5021	4425	79
H(72B)	6078	4968	4089	79
H(72C)	7294	5576	4145	79
H(61A)	7784	9668	6875	92
H(61B)	6390	10053	6721	92
H(61C)	7700	10535	6857	92
H(76A)	6032	8874	6010	86
H(76B)	6887	8197	5862	86
H(76C)	6425	8190	6229	86
H(66A)	10981	9218	6677	76
H(66B)	12170	8601	6612	76
H(66C)	11861	8871	6968	76
H(77A)	3386	8246	5430	95
H(77B)	5103	8143	5386	95
H(77C)	4479	8871	5543	95
H(62A)	7596	11258	6371	92
H(62B)	6616	10807	6126	92
H(62C)	8226	11049	6028	92
H(14B)	10530(50)	4810(30)	5108(11)	35(12)
H(61D)	6692	10771	6010	92
H(61E)	8305	10811	5867	92
H(61F)	7742	10080	6032	92
H(62D)	6358	10433	6684	92
H(62E)	7479	9800	6595	92
H(62F)	7812	10327	6889	92
H(14A)	5730(40)	5020(20)	7454(10)	19(9)

H(56D)	5950	8121	6704	61
H(56E)	4512	7874	6889	61
H(56F)	4487	8586	6671	61
H(57D)	7159	7890	7303	61
H(57E)	6540	8305	7609	61
H(57F)	5541	7731	7425	61
H(66D)	10302	7572	6393	76
H(66E)	9631	7698	6741	76
H(66F)	8578	7548	6444	76
H(67D)	11781	8962	6578	76
H(67E)	10707	9535	6742	76
H(67F)	10866	8735	6887	76

Table S6. Torsion angles [°] for **7**.

Cl(1B)-C(8B)-C(7B)-C(6B)	177.1(3)
Cl(4B)-C(30B)-C(31B)-C(32B)	-178.1(3)
Cl(4A)-C(30A)-C(29A)-C(28A)	-178.5(3)
N(12A)-C(11A)-C(10A)-C(9A)	177.1(3)
N(12B)-C(11B)-C(6B)-N(5B)	-2.5(4)
N(12B)-C(11B)-C(6B)-C(7B)	175.6(3)
N(12B)-C(11B)-C(10B)-C(9B)	-178.0(3)
N(2A)-N(1A)-C(13A)-N(12A)	2.0(4)
N(2A)-N(1A)-C(13A)-N(14A)	177.9(3)
N(2A)-C(21A)-C(26A)-C(25A)	-178.5(3)
N(2A)-C(21A)-C(22A)-C(23A)	177.4(3)
N(2B)-N(1B)-C(13B)-N(12B)	-1.8(4)
N(2B)-N(1B)-C(13B)-N(14B)	-178.3(3)
N(2B)-C(21B)-C(22B)-C(23B)	-176.6(3)
N(1A)-N(2A)-C(21A)-C(26A)	-172.6(3)
N(1A)-N(2A)-C(21A)-C(22A)	8.1(5)
N(1A)-N(2A)-C(3A)-N(12A)	4.0(4)
N(1A)-N(2A)-C(3A)-N(4A)	-176.8(3)
N(14B)-C(15B)-C(16B)-C(17B)	-179.4(3)
N(14B)-C(15B)-C(20B)-C(19B)	179.5(3)
N(14A)-C(15A)-C(16A)-C(17A)	-179.7(3)
N(14A)-C(15A)-C(20A)-C(19A)	179.2(3)
N(4B)-N(5B)-C(27B)-C(28B)	9.6(5)
N(4B)-N(5B)-C(27B)-C(32B)	-167.8(3)
N(4B)-N(5B)-C(6B)-C(11B)	41.1(4)
N(4B)-N(5B)-C(6B)-C(7B)	-136.9(3)
N(4A)-N(5A)-C(27A)-C(28A)	-17.4(4)
N(4A)-N(5A)-C(27A)-C(32A)	159.5(3)
N(4A)-N(5A)-C(6A)-C(7A)	135.8(3)
N(4A)-N(5A)-C(6A)-C(11A)	-42.4(4)
N(5A)-N(4A)-C(3A)-N(12A)	-7.4(5)
N(5A)-N(4A)-C(3A)-N(2A)	173.6(3)
N(5A)-C(27A)-C(32A)-C(31A)	-178.0(3)
N(5A)-C(6A)-C(11A)-N(12A)	2.0(4)
N(5A)-C(6A)-C(11A)-C(10A)	177.8(3)
N(5B)-N(4B)-C(3B)-N(12B)	5.8(5)
N(5B)-N(4B)-C(3B)-N(2B)	-175.9(3)
N(5B)-C(27B)-C(32B)-C(31B)	-178.8(4)

N(5B)-C(6B)-C(7B)-C(8B)	-178.9(3)
N(1B)-N(2B)-C(3B)-N(12B)	-3.9(4)
N(1B)-N(2B)-C(3B)-N(4B)	177.5(3)
N(1B)-N(2B)-C(21B)-C(26B)	-178.8(3)
N(1B)-N(2B)-C(21B)-C(22B)	1.3(5)
C(13A)-N(12A)-C(3A)-N(2A)	-2.6(4)
C(13A)-N(12A)-C(3A)-N(4A)	178.1(3)
C(13A)-N(12A)-C(11A)-C(6A)	174.1(3)
C(13A)-N(12A)-C(11A)-C(10A)	-1.4(6)
C(13A)-N(14A)-C(15A)-C(16A)	17.3(5)
C(13A)-N(14A)-C(15A)-C(20A)	-163.4(3)
C(21A)-N(2A)-N(1A)-C(13A)	-178.9(3)
C(21A)-N(2A)-C(3A)-N(12A)	178.4(3)
C(21A)-N(2A)-C(3A)-N(4A)	-2.4(6)
C(21A)-C(26A)-C(25A)-C(24A)	1.1(5)
C(7A)-C(6A)-C(11A)-N(12A)	-176.3(3)
C(7A)-C(6A)-C(11A)-C(10A)	-0.5(5)
C(28A)-C(27A)-C(32A)-C(31A)	-1.1(5)
C(27A)-N(5A)-C(6A)-C(7A)	-5.9(5)
C(27A)-N(5A)-C(6A)-C(11A)	175.9(3)
C(27A)-C(28A)-C(29A)-C(30A)	-0.7(5)
C(3A)-N(12A)-C(13A)-N(1A)	0.3(4)
C(3A)-N(12A)-C(13A)-N(14A)	-175.9(3)
C(3A)-N(12A)-C(11A)-C(6A)	32.8(4)
C(3A)-N(12A)-C(11A)-C(10A)	-142.7(4)
C(3A)-N(2A)-N(1A)-C(13A)	-3.8(4)
C(3A)-N(2A)-C(21A)-C(26A)	13.4(5)
C(3A)-N(2A)-C(21A)-C(22A)	-166.0(3)
C(3A)-N(4A)-N(5A)-C(27A)	-170.6(3)
C(3A)-N(4A)-N(5A)-C(6A)	44.6(4)
C(3B)-N(12B)-C(11B)-C(6B)	-31.3(4)
C(3B)-N(12B)-C(11B)-C(10B)	143.9(3)
C(3B)-N(12B)-C(13B)-N(14B)	176.3(3)
C(3B)-N(12B)-C(13B)-N(1B)	-0.5(4)
C(3B)-N(2B)-N(1B)-C(13B)	3.6(4)
C(3B)-N(2B)-C(21B)-C(26B)	-4.7(5)
C(3B)-N(2B)-C(21B)-C(22B)	175.4(3)
C(3B)-N(4B)-N(5B)-C(27B)	164.3(3)
C(3B)-N(4B)-N(5B)-C(6B)	-42.3(4)
C(6A)-N(5A)-C(27A)-C(28A)	125.4(4)

C(6A)-N(5A)-C(27A)-C(32A)	-57.7(4)
C(6A)-C(7A)-C(8A)-Cl(1A)	180.0(3)
C(6A)-C(7A)-C(8A)-C(9A)	1.7(6)
C(6A)-C(11A)-C(10A)-C(9A)	1.9(5)
C(9B)-C(8B)-C(7B)-C(6B)	-3.7(5)
C(25B)-C(26B)-C(21B)-N(2B)	177.1(3)
C(25B)-C(26B)-C(21B)-C(22B)	-3.0(5)
C(26A)-C(21A)-C(22A)-C(23A)	-1.9(5)
C(26A)-C(25A)-C(24A)-Cl(3A)	178.7(3)
C(26A)-C(25A)-C(24A)-C(23A)	-2.0(5)
C(15A)-N(14A)-C(13A)-N(12A)	174.0(3)
C(15A)-N(14A)-C(13A)-N(1A)	-1.6(6)
C(15A)-C(16A)-C(17A)-C(18A)	-0.1(6)
C(11B)-N(12B)-C(3B)-N(2B)	-147.4(3)
C(11B)-N(12B)-C(3B)-N(4B)	31.3(5)
C(11B)-N(12B)-C(13B)-N(14B)	-40.0(5)
C(11B)-N(12B)-C(13B)-N(1B)	143.2(3)
C(11B)-C(6B)-C(7B)-C(8B)	3.1(5)
C(29B)-C(28B)-C(27B)-N(5B)	-178.5(3)
C(29B)-C(28B)-C(27B)-C(32B)	-1.0(5)
C(29B)-C(30B)-C(31B)-C(32B)	1.3(6)
C(26B)-C(25B)-C(24B)-Cl(3B)	-178.0(3)
C(26B)-C(25B)-C(24B)-C(23B)	2.5(5)
C(26B)-C(21B)-C(22B)-C(23B)	3.5(5)
C(11A)-N(12A)-C(13A)-N(1A)	-143.3(3)
C(11A)-N(12A)-C(13A)-N(14A)	40.5(5)
C(11A)-N(12A)-C(3A)-N(2A)	147.4(3)
C(11A)-N(12A)-C(3A)-N(4A)	-31.8(5)
C(11A)-C(10A)-C(9A)-C(8A)	-1.5(5)
C(31A)-C(30A)-C(29A)-C(28A)	0.2(6)
C(16A)-C(15A)-C(20A)-C(19A)	-1.4(5)
C(16A)-C(17A)-C(18A)-Cl(2A)	179.0(3)
C(16A)-C(17A)-C(18A)-C(19A)	-0.2(6)
C(21B)-N(2B)-N(1B)-C(13B)	178.7(3)
C(21B)-N(2B)-C(3B)-N(12B)	-178.3(3)
C(21B)-N(2B)-C(3B)-N(4B)	3.1(6)
C(24B)-C(25B)-C(26B)-C(21B)	0.0(5)
C(24B)-C(23B)-C(22B)-C(21B)	-1.0(5)
C(28B)-C(29B)-C(30B)-Cl(4B)	-179.3(3)
C(28B)-C(29B)-C(30B)-C(31B)	1.3(6)

C(28B)-C(27B)-C(32B)-C(31B)	3.7(6)
C(27B)-N(5B)-C(6B)-C(11B)	-168.0(3)
C(27B)-N(5B)-C(6B)-C(7B)	14.0(5)
C(27B)-C(32B)-C(31B)-C(30B)	-3.8(7)
C(10A)-C(9A)-C(8A)-Cl(1A)	-178.6(3)
C(10A)-C(9A)-C(8A)-C(7A)	-0.3(6)
C(13B)-N(12B)-C(3B)-N(2B)	2.6(4)
C(13B)-N(12B)-C(3B)-N(4B)	-178.7(3)
C(13B)-N(12B)-C(11B)-C(6B)	-172.7(3)
C(13B)-N(12B)-C(11B)-C(10B)	2.5(5)
C(13B)-N(14B)-C(15B)-C(16B)	-20.8(6)
C(13B)-N(14B)-C(15B)-C(20B)	161.8(3)
C(15B)-N(14B)-C(13B)-N(12B)	-177.0(3)
C(15B)-N(14B)-C(13B)-N(1B)	-0.8(6)
C(15B)-C(16B)-C(17B)-C(18B)	0.8(6)
C(22A)-C(21A)-C(26A)-C(25A)	0.8(5)
C(22A)-C(23A)-C(24A)-Cl(3A)	-179.8(3)
C(22A)-C(23A)-C(24A)-C(25A)	0.9(5)
C(30A)-C(31A)-C(32A)-C(27A)	0.6(6)
C(6B)-N(5B)-C(27B)-C(28B)	-141.8(4)
C(6B)-N(5B)-C(27B)-C(32B)	40.8(5)
C(6B)-C(11B)-C(10B)-C(9B)	-3.1(5)
C(32A)-C(31A)-C(30A)-Cl(4A)	178.6(3)
C(32A)-C(31A)-C(30A)-C(29A)	-0.2(6)
C(16B)-C(15B)-C(20B)-C(19B)	1.9(5)
C(16B)-C(17B)-C(18B)-Cl(2B)	179.3(3)
C(16B)-C(17B)-C(18B)-C(19B)	0.3(6)
C(10B)-C(9B)-C(8B)-Cl(1B)	180.0(3)
C(10B)-C(9B)-C(8B)-C(7B)	0.8(5)
C(10B)-C(11B)-C(6B)-N(5B)	-177.9(3)
C(10B)-C(11B)-C(6B)-C(7B)	0.2(5)
C(8B)-C(9B)-C(10B)-C(11B)	2.6(5)
C(22B)-C(23B)-C(24B)-Cl(3B)	178.6(3)
C(22B)-C(23B)-C(24B)-C(25B)	-2.0(5)
C(24A)-C(23A)-C(22A)-C(21A)	1.1(5)
C(29A)-C(28A)-C(27A)-N(5A)	178.0(3)
C(29A)-C(28A)-C(27A)-C(32A)	1.1(5)
C(20A)-C(15A)-C(16A)-C(17A)	0.9(5)
C(20A)-C(19A)-C(18A)-Cl(2A)	-179.5(3)
C(20A)-C(19A)-C(18A)-C(17A)	-0.2(6)

C(18A)-C(19A)-C(20A)-C(15A)	1.1(6)
C(30B)-C(29B)-C(28B)-C(27B)	-1.4(6)
C(20B)-C(15B)-C(16B)-C(17B)	-1.9(5)
C(20B)-C(19B)-C(18B)-Cl(2B)	-179.4(3)
C(20B)-C(19B)-C(18B)-C(17B)	-0.4(6)
C(18B)-C(19B)-C(20B)-C(15B)	-0.8(6)
C(8A)-C(7A)-C(6A)-N(5A)	-179.4(3)
C(8A)-C(7A)-C(6A)-C(11A)	-1.2(5)

Table S7. Hydrogen bonds for **7** [Å and °].

D-H...A	d(D-H)	d(H...A)	d(D...A)	<(DHA)
N(14B)-H(14B)...O(50)	0.84(5)	1.95(5)	2.789(4)	173(4)
N(14A)-H(14A)...O(55)#1	0.85(4)	1.97(4)	2.814(4)	172(4)
N(14A)-H(14A)...O(55A)#1	0.85(4)	1.93(5)	2.76(3)	165(4)

Symmetry transformations used to generate equivalent atoms:

#1 -x+1,y-1/2,-z+3/2

S3. EPR spectra

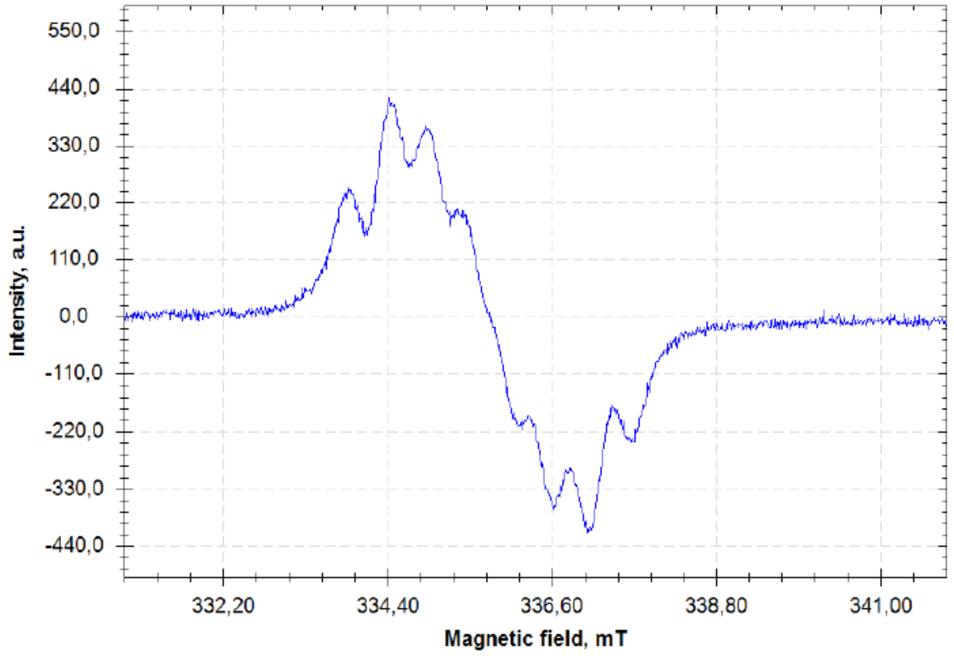

Attenuation: 15 db
Center magnetic field: 336,38 mT
Sweep width: 11,00 mT
Modulation amplitude: 100 uT
Modulation frequency: 100000 Hz
Points: 1000
Sweep time: 165,0 s
MW frequency, GHz: 9,414947

Power MW, mW: 2,8
Smoothig points: 0
Time constant: 0,127

Intensity, a.u.: 838
Line width, mT: 2,65
g-factor: 2,0035

EPR spectrum of compound 6l

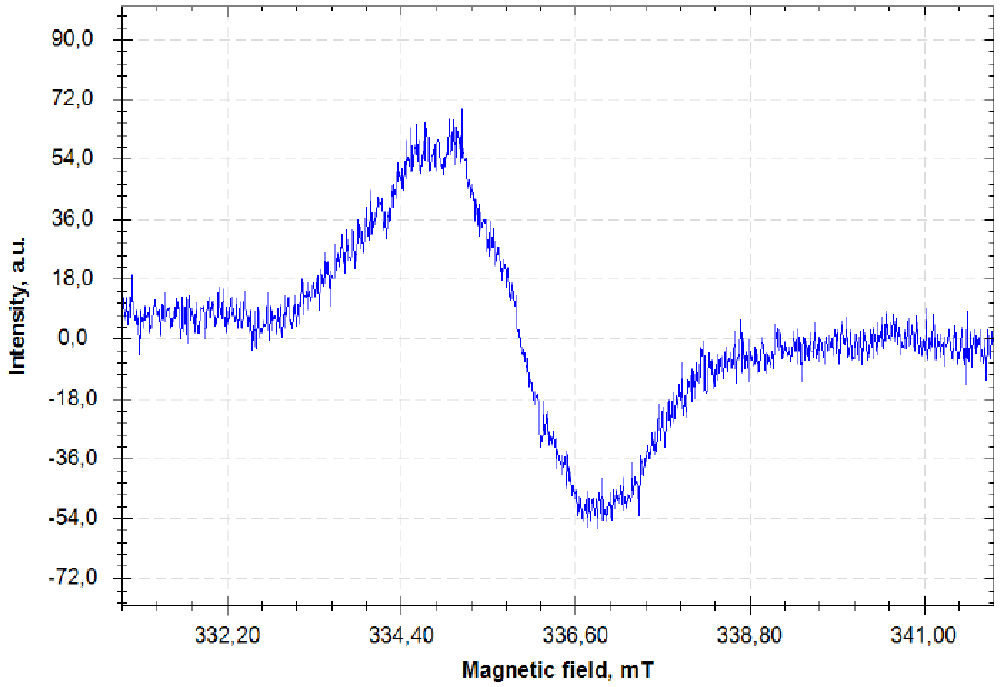

Attenuation: 15 db
Center magnetic field: 336,38 mT
Sweep width: 11,00 mT
Modulation amplitude: 100 uT
Modulation frequency: 100000 Hz
Points: 1000
Sweep time: 165,0 s
MW frequency, GHz: 9,417775

Power MW, mW: 2,8
Smoothig points: 0
Time constant: 0,127

Intensity, a.u.: 74
Line width, mT: 0,01
g-factor: 2,0024

EPR spectrum of compound 7

S4. NMR and HRMS spectra

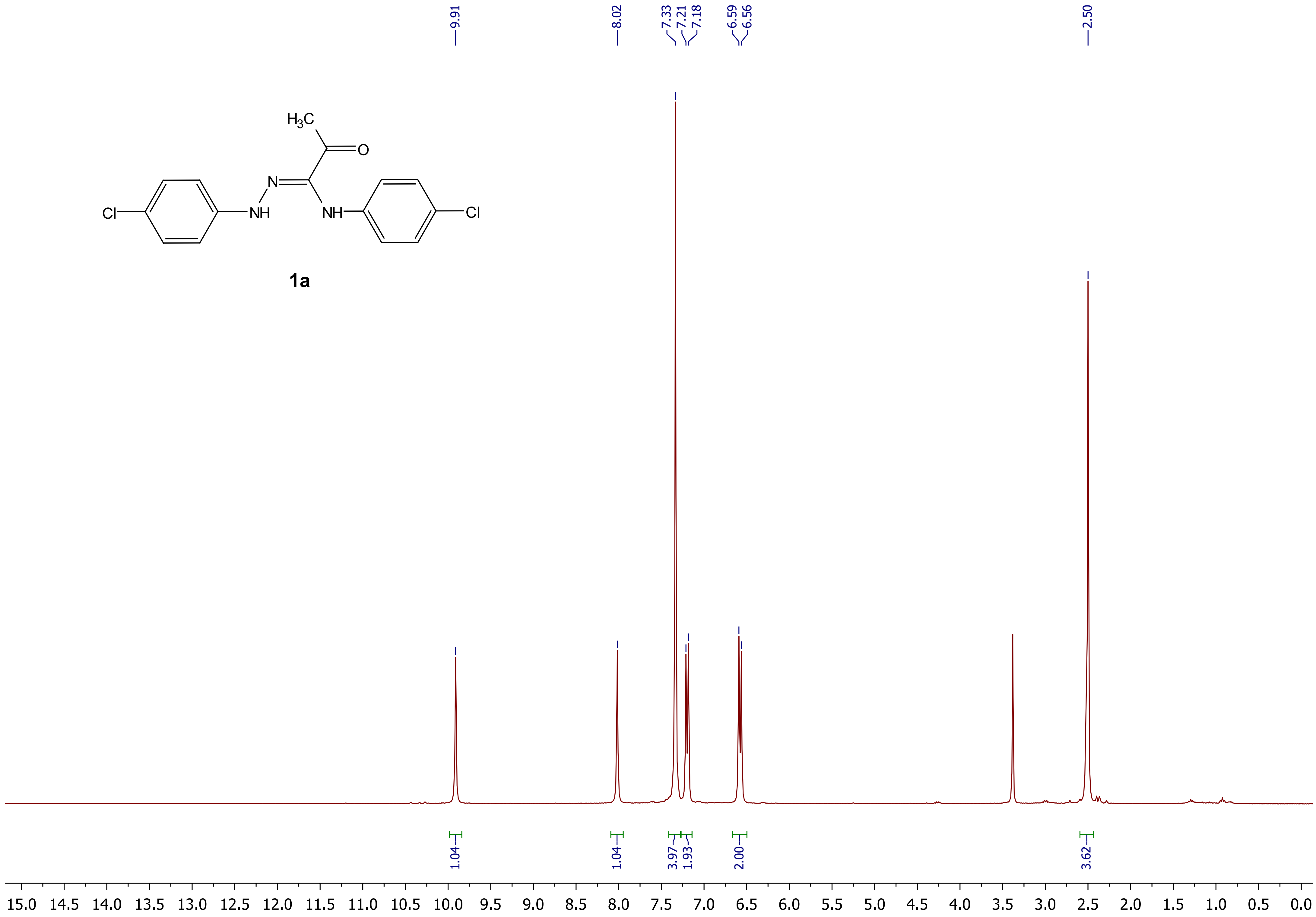
9.91
8.02
7.33
7.21
7.18
6.59
6.56
2.50
H3C
O
N
NH
NH
Cl
Cl
1a
1.04
1.04
3.97
1.93
2.00
3.62
15.0 14.5 14.0 13.5 13.0 12.5 12.0 11.5 11.0 10.5 10.0 9.5 9.0 8.5 8.0 7.5 7.0 6.5 6.0 5.5 5.0 4.5 4.0 3.5 3.0 2.5 2.0 1.5 1.0 0.5 0.0

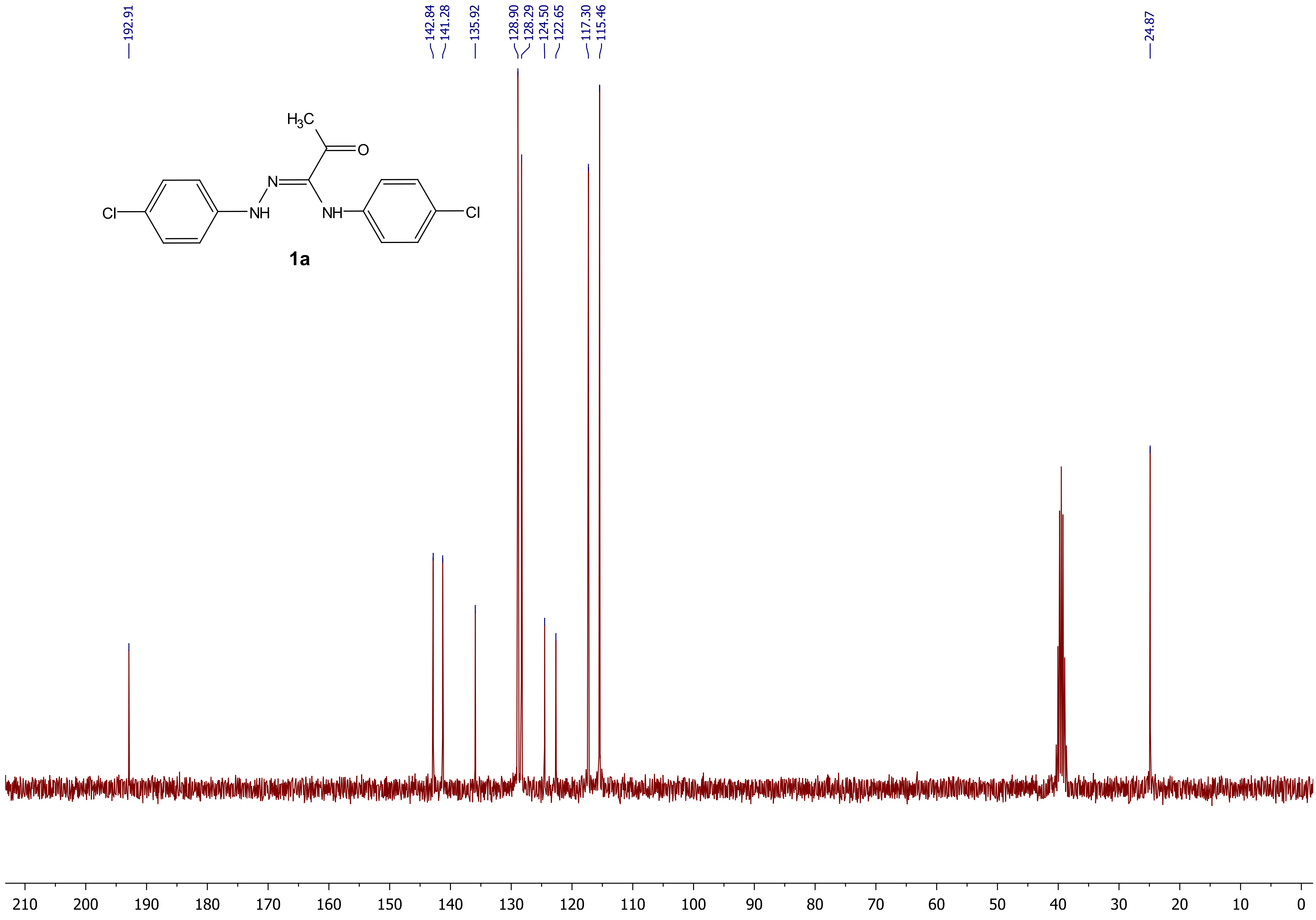
192.91
142.84
141.28
135.92
128.90
128.29
124.50
122.65
117.30
115.46
24.87
H3C
O
N
NH
NH
Cl
Cl
1a
210 200 190 180 170 160 150 140 130 120 110 100 90 80 70 60 50 40 30 20 10 0

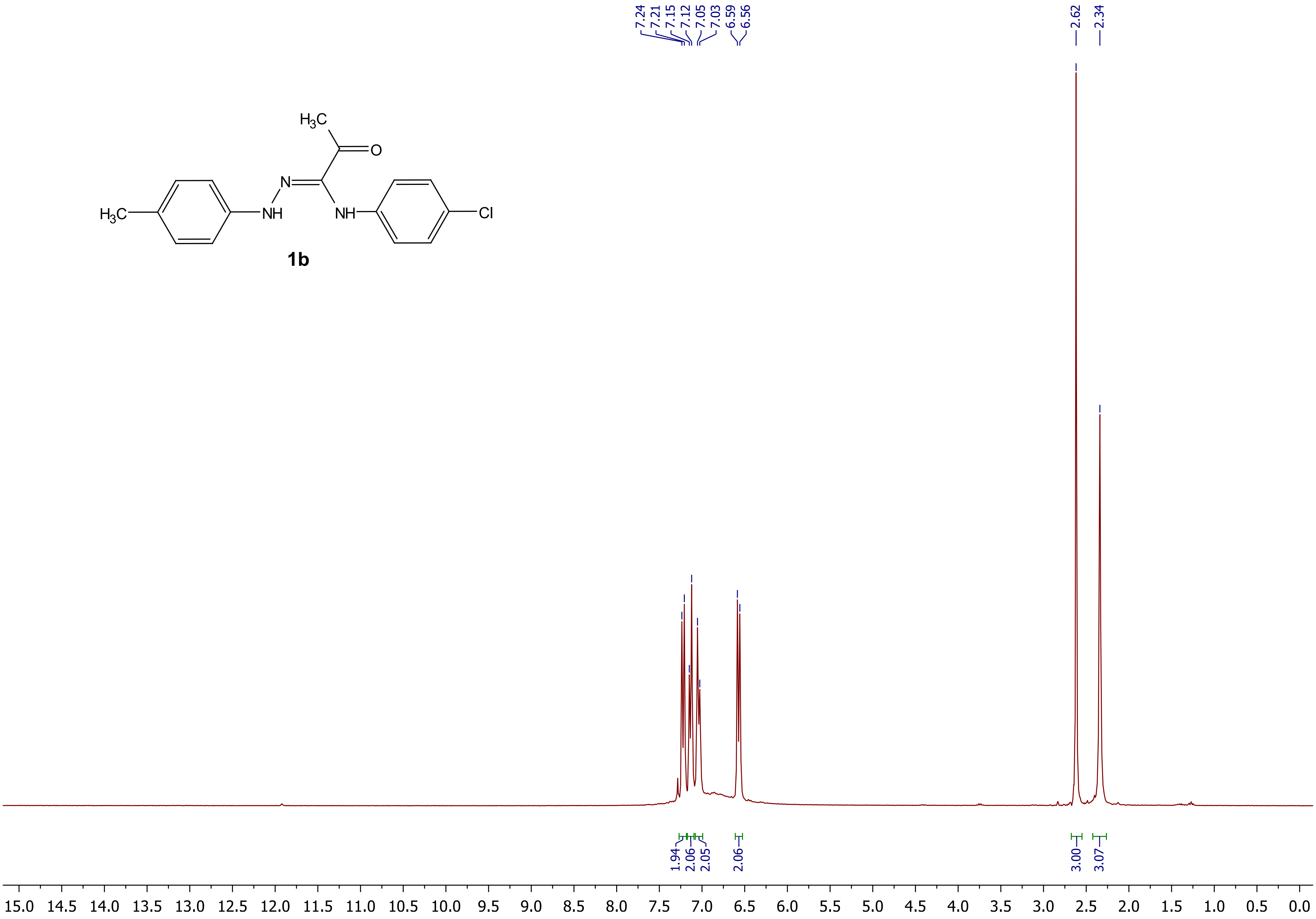

H3C
O
N
NH
NH
H3C
Cl
1b
7.24
7.21
7.15
7.12
7.05
7.03
6.59
6.56
2.62
2.34
1.94
2.06
2.05
2.06
3.00
3.07
15.0 14.5 14.0 13.5 13.0 12.5 12.0 11.5 11.0 10.5 10.0 9.5 9.0 8.5 8.0 7.5 7.0 6.5 6.0 5.5 5.0 4.5 4.0 3.5 3.0 2.5 2.0 1.5 1.0 0.5 0.0

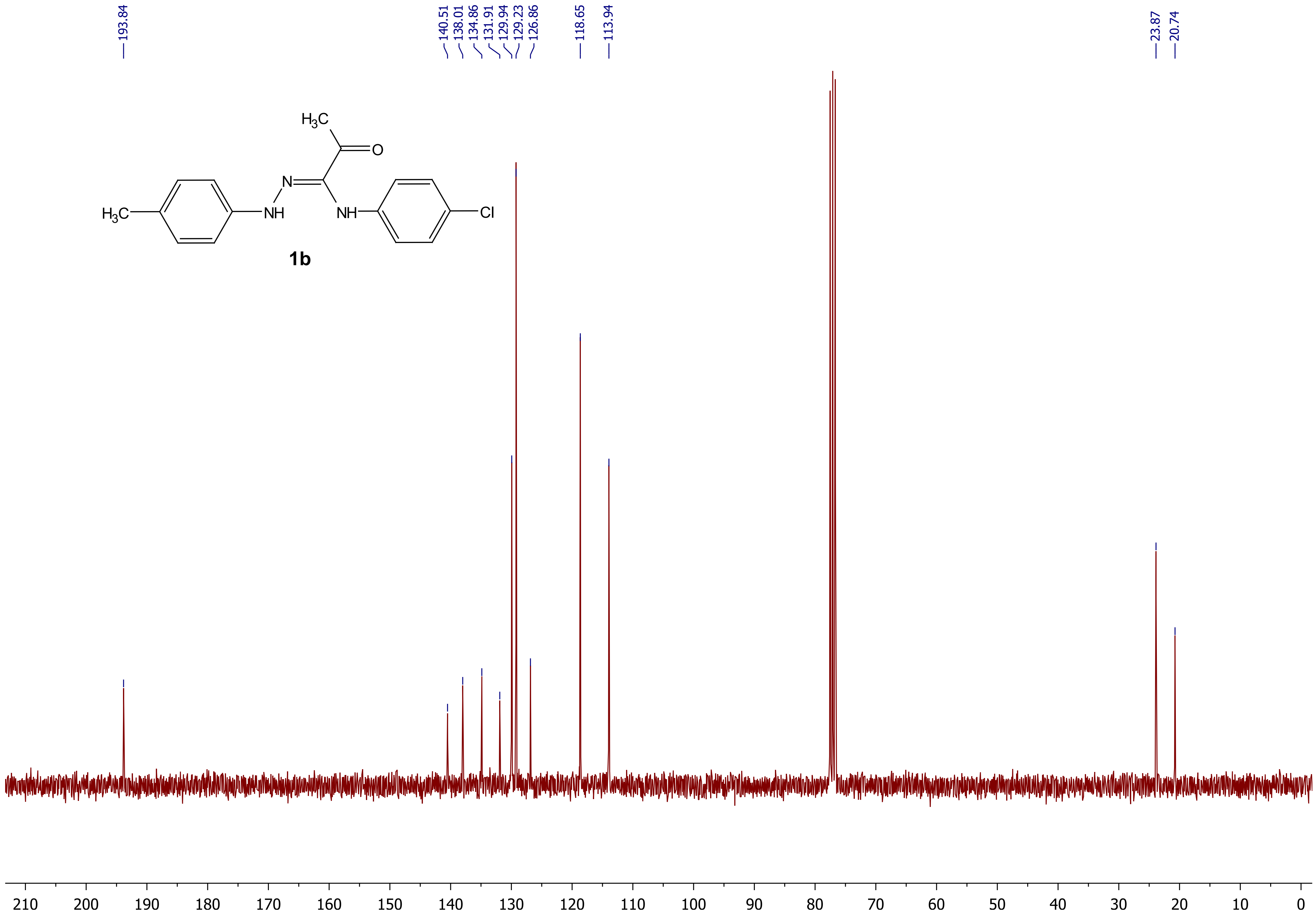
193.84
140.51
138.01
134.86
131.91
129.94
129.23
126.86
118.65
113.94
23.87
20.74
H3C
O
N
NH
NH
Cl
1b
210 200 190 180 170 160 150 140 130 120 110 100 90 80 70 60 50 40 30 20 10 0

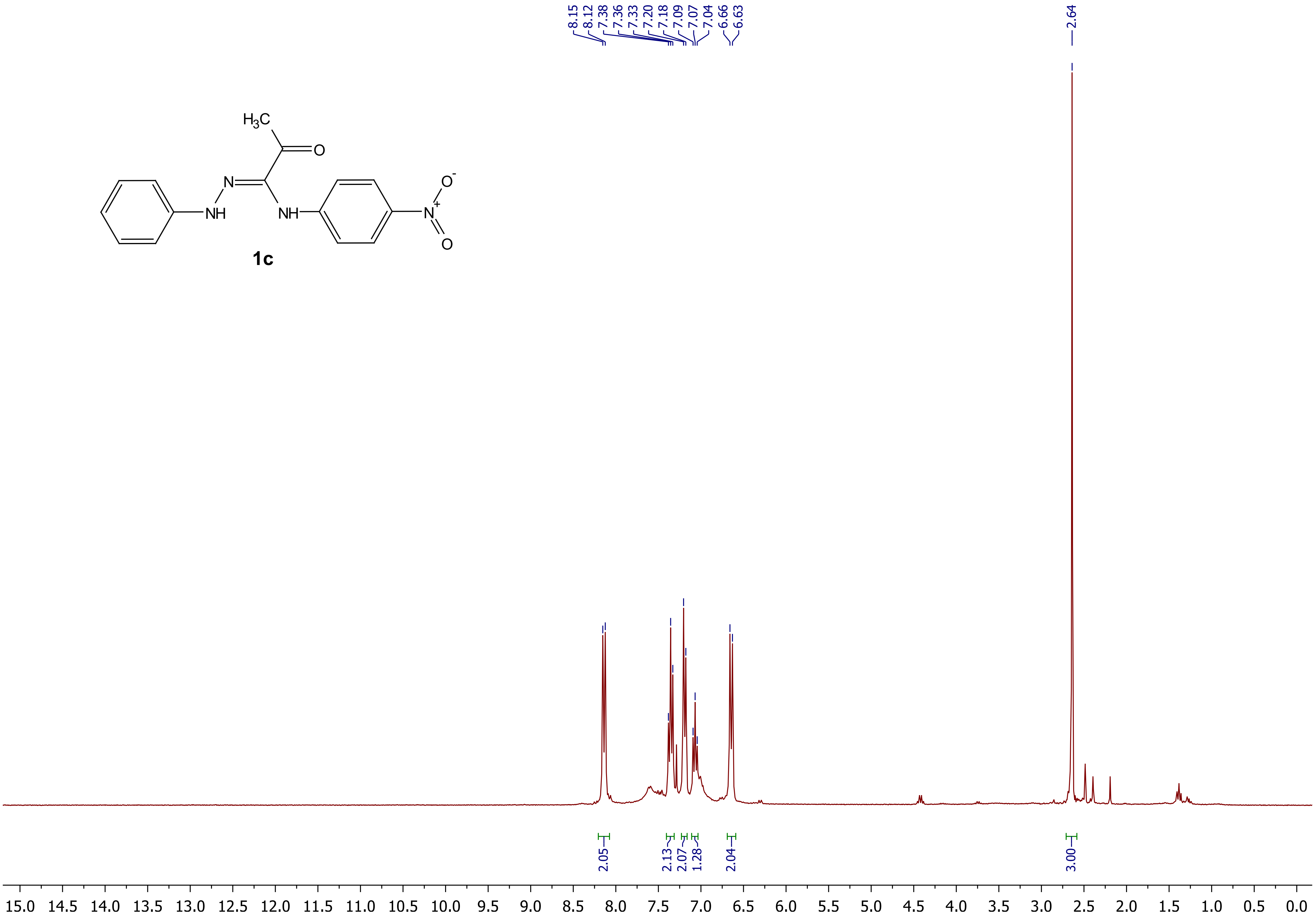
8.15
8.12
7.38
7.36
7.33
7.20
7.18
7.09
7.07
7.04
6.66
6.63
2.64
H3C
O
N
NH
NH
N+
O-
O
1c
2.05
2.13
2.07
1.28
2.04
3.00
15.0 14.5 14.0 13.5 13.0 12.5 12.0 11.5 11.0 10.5 10.0 9.5 9.0 8.5 8.0 7.5 7.0 6.5 6.0 5.5 5.0 4.5 4.0 3.5 3.0 2.5 2.0 1.5 1.0 0.5 0.0

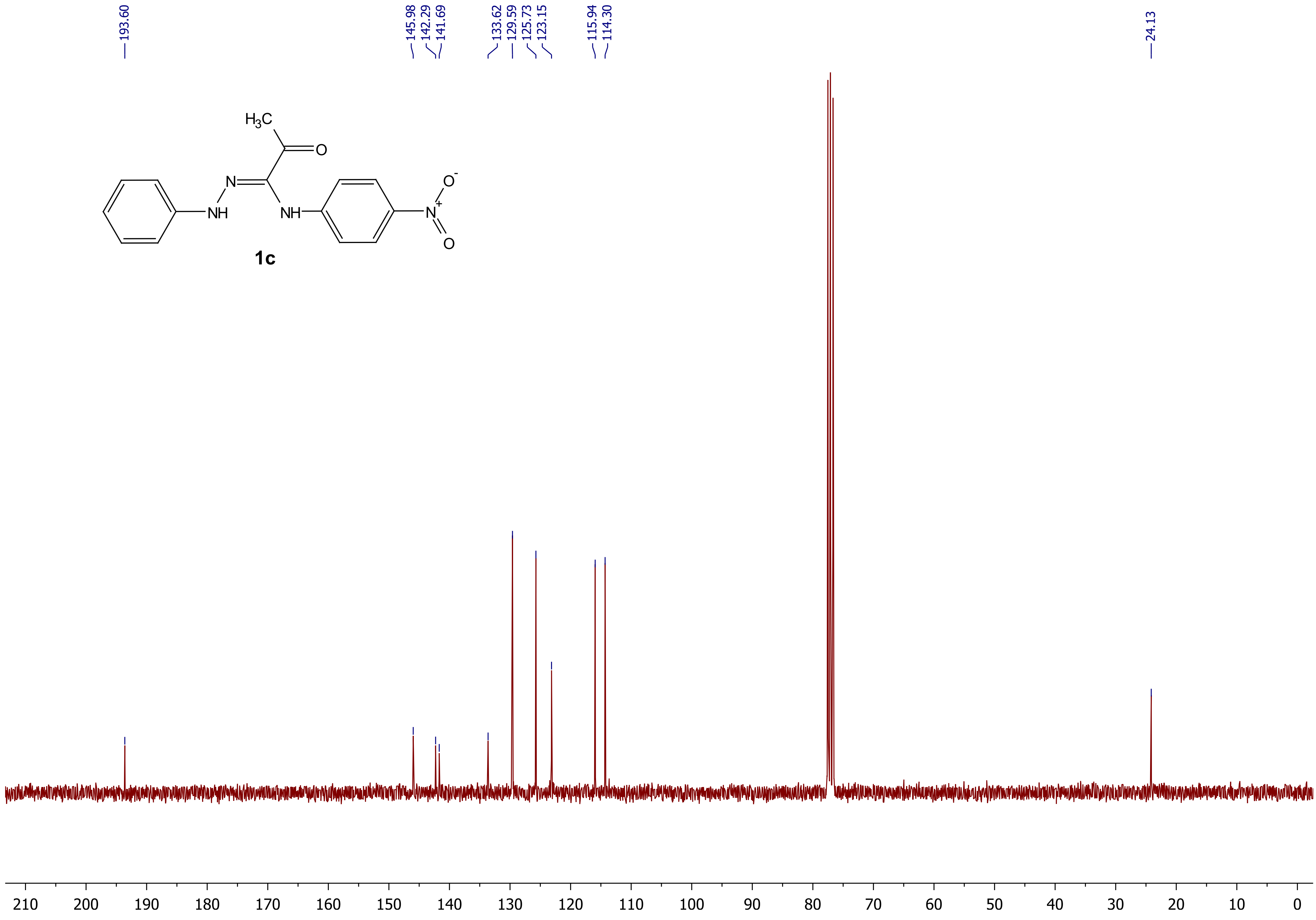
193.60
145.98
142.29
141.69
133.62
129.59
125.73
123.15
115.94
114.30
24.13
H3C
O
N
NH
NH
N+
O-
O
1c
210 200 190 180 170 160 150 140 130 120 110 100 90 80 70 60 50 40 30 20 10 0

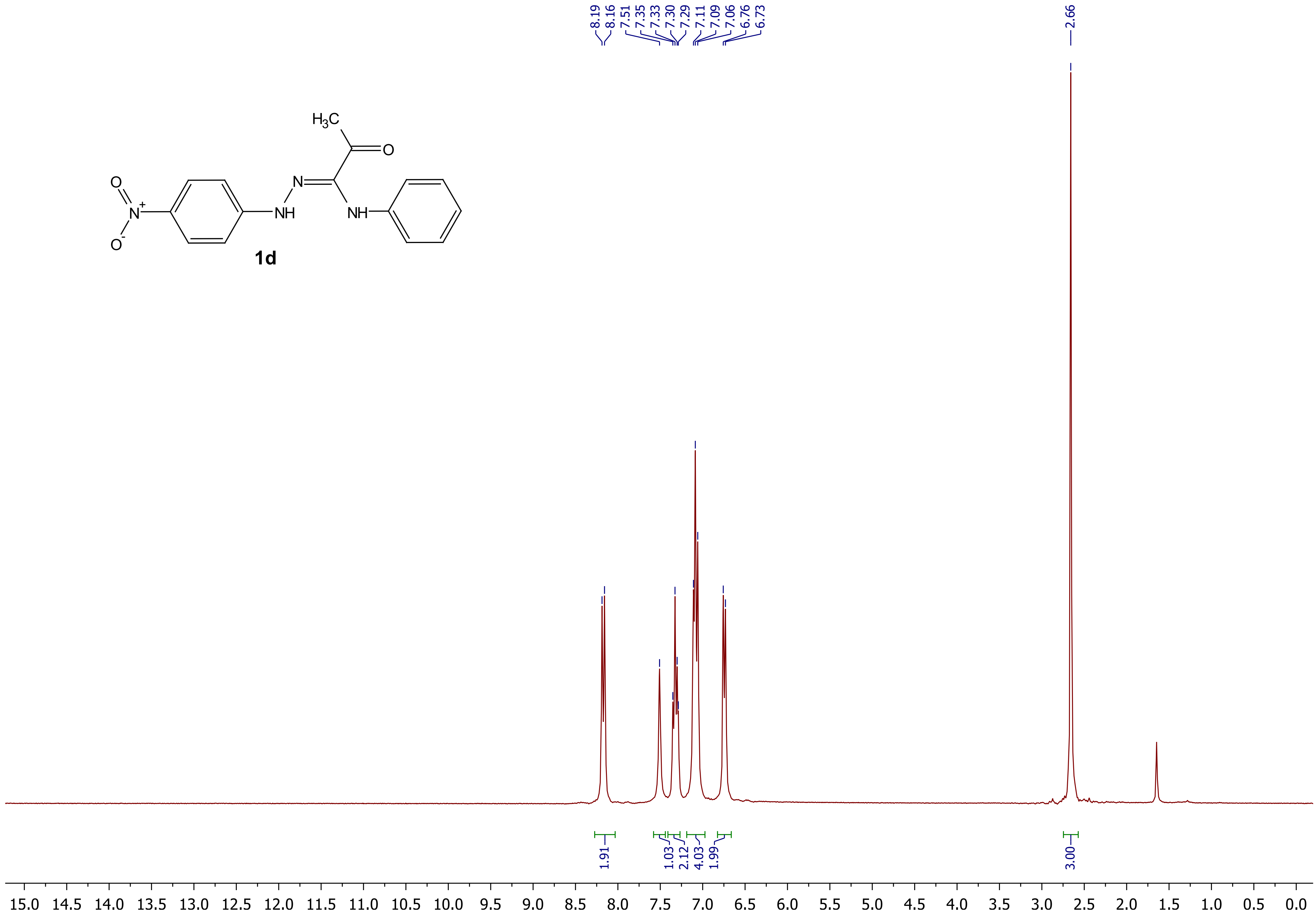

8.19
8.16
7.51
7.35
7.33
7.30
7.29
7.11
7.09
7.06
6.76
6.73
2.66
H3C
O
N
NH
NH
N+
O-
1d
1.91
1.03
2.12
4.03
1.99
3.00
15.0 14.5 14.0 13.5 13.0 12.5 12.0 11.5 11.0 10.5 10.0 9.5 9.0 8.5 8.0 7.5 7.0 6.5 6.0 5.5 5.0 4.5 4.0 3.5 3.0 2.5 2.0 1.5 1.0 0.5 0.0

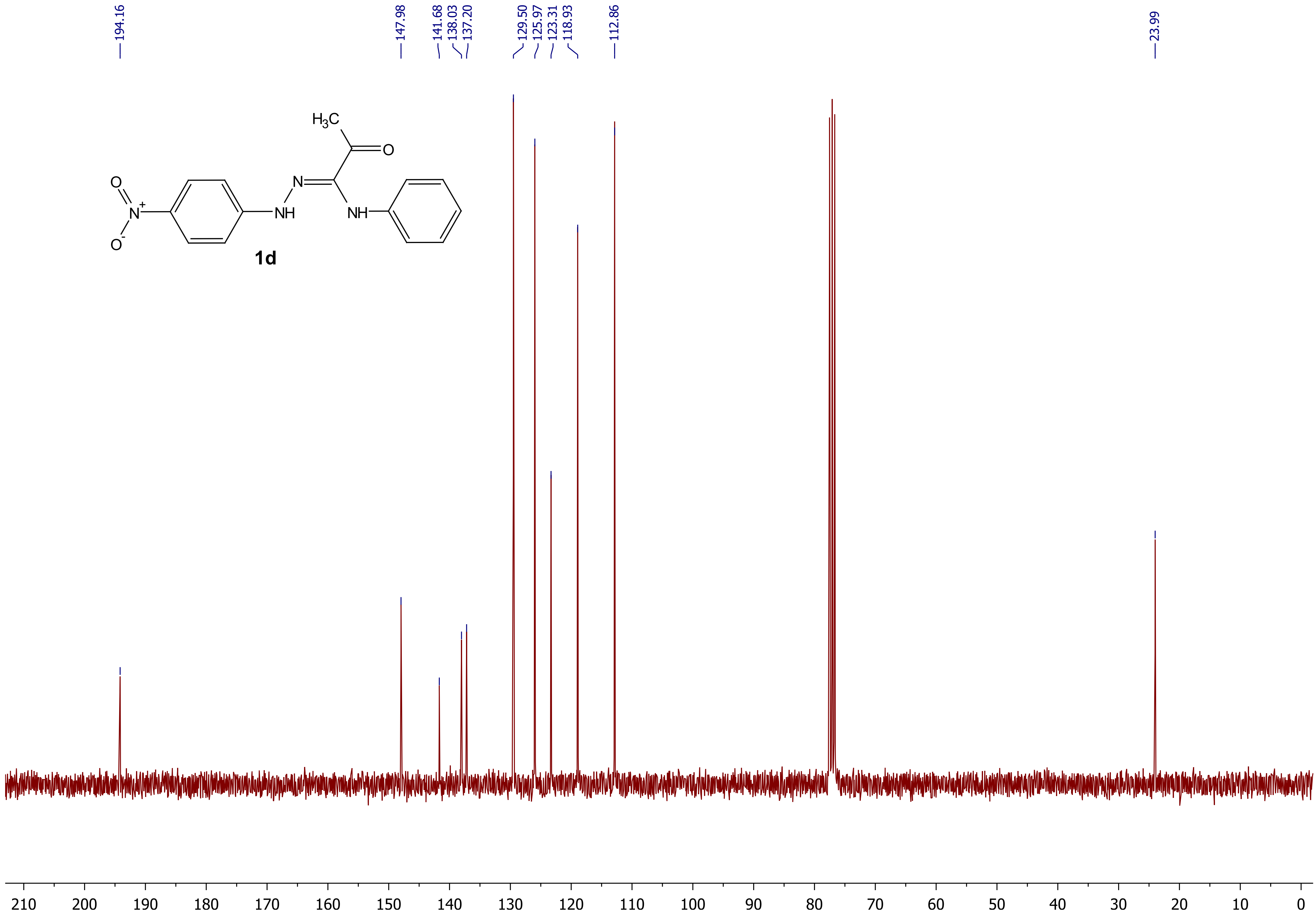
194.16
147.98
141.68
138.03
137.20
129.50
125.97
123.31
118.93
112.86
23.99
H_3C
O
N
NH
NH
1d
210
200
190
180
170
160
150
140
130
120
110
100
90
80
70
60
50
40
30
20
10
0

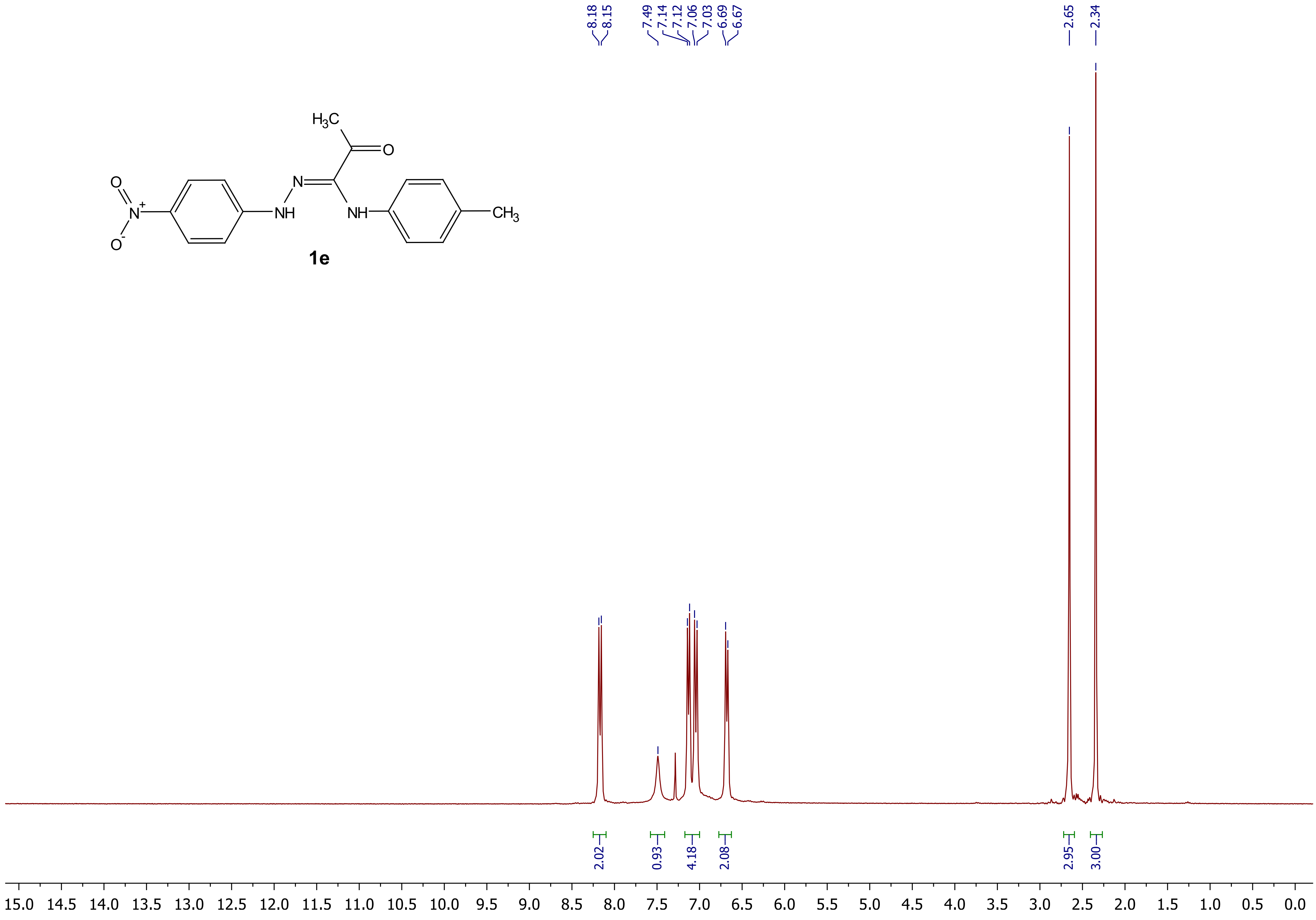

8.18
8.15
7.49
7.14
7.12
7.06
7.03
6.69
6.67
2.65
2.34
H3C
O
N
NH
NH
CH3
1e
2.02
0.93
4.18
2.08
2.95
3.00
15.0 14.5 14.0 13.5 13.0 12.5 12.0 11.5 11.0 10.5 10.0 9.5 9.0 8.5 8.0 7.5 7.0 6.5 6.0 5.5 5.0 4.5 4.0 3.5 3.0 2.5 2.0 1.5 1.0 0.5 0.0

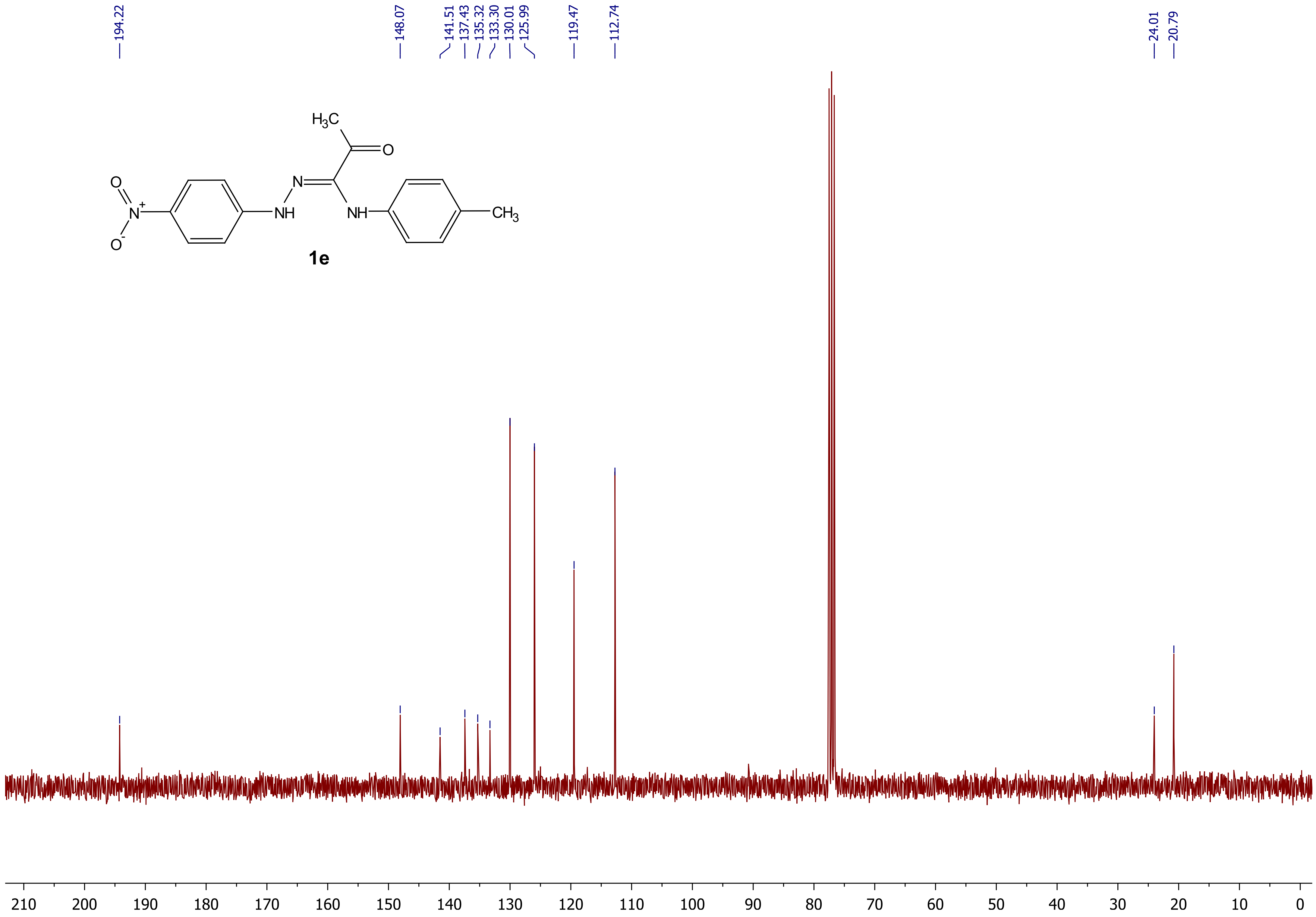
194.22
148.07
141.51
137.43
135.32
133.30
130.01
125.99
119.47
112.74
24.01
20.79
H3C
O
N
NH
NH
CH3
1e
210 200 190 180 170 160 150 140 130 120 110 100 90 80 70 60 50 40 30 20 10 0

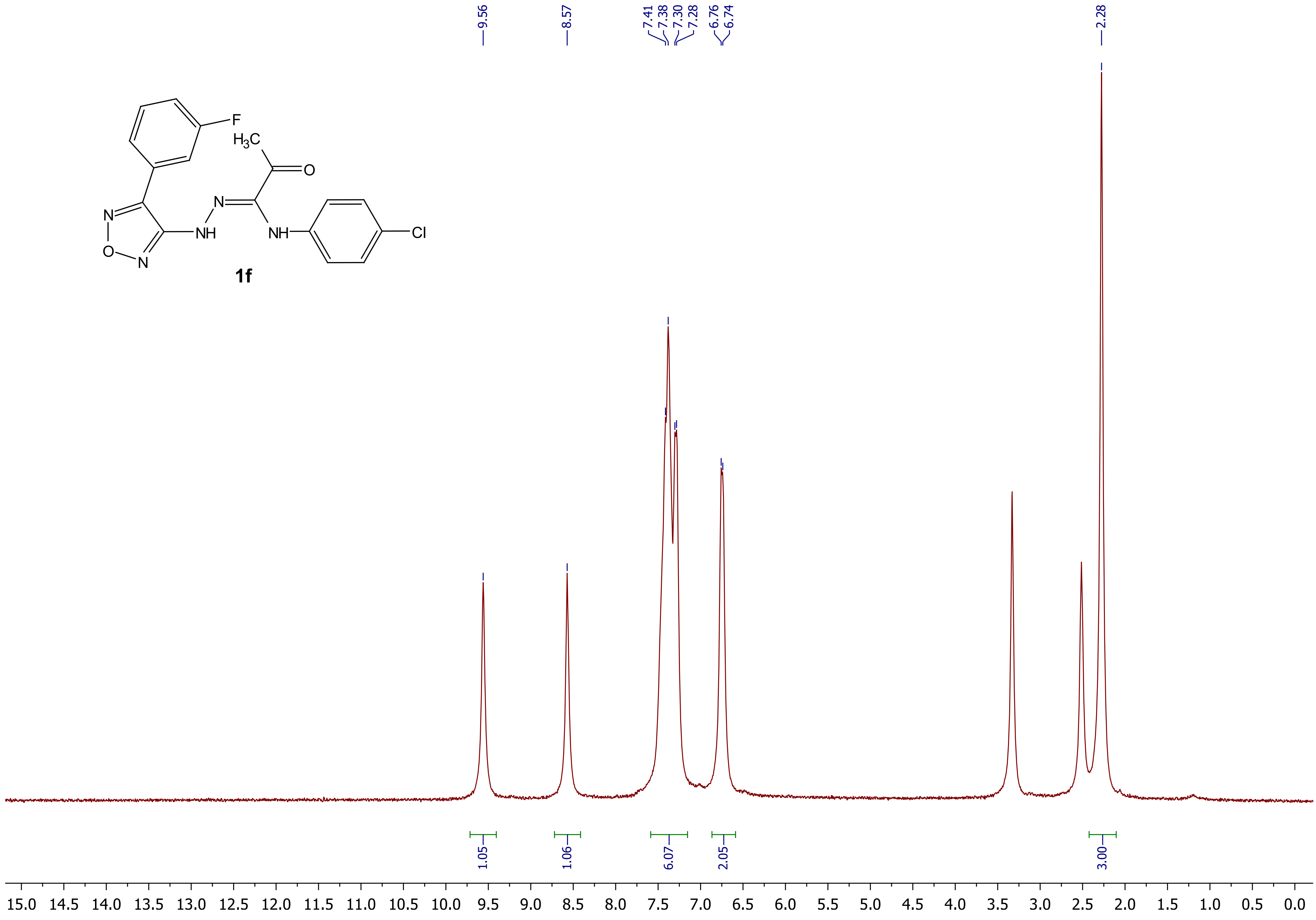
9.56
8.57
7.41
7.38
7.30
7.28
6.76
6.74
2.28
F
H3C
O
N
NH
NH
Cl
N
O
N
1f
1.05
1.06
6.07
2.05
3.00
15.0 14.5 14.0 13.5 13.0 12.5 12.0 11.5 11.0 10.5 10.0 9.5 9.0 8.5 8.0 7.5 7.0 6.5 6.0 5.5 5.0 4.5 4.0 3.5 3.0 2.5 2.0 1.5 1.0 0.5 0.0

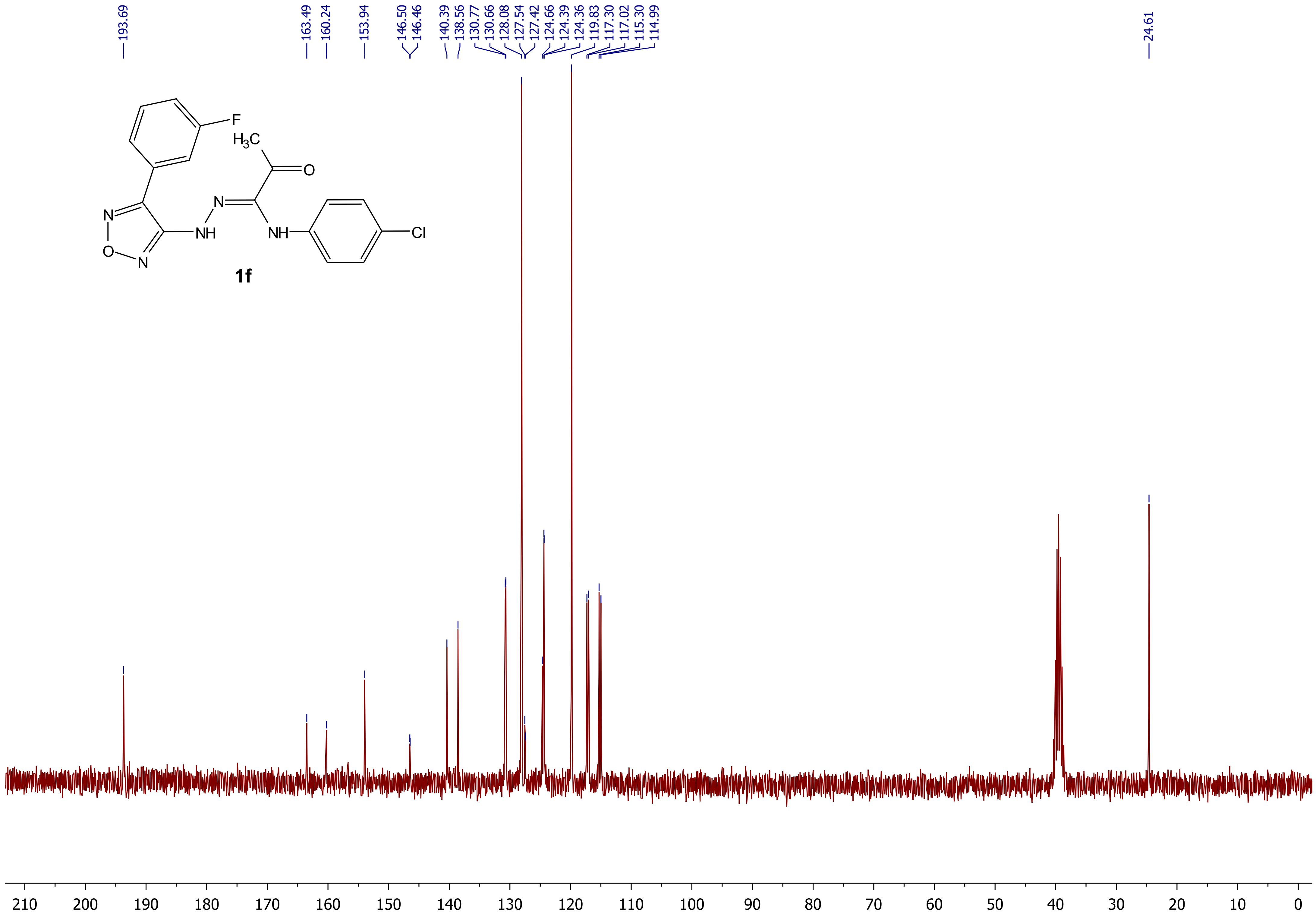

193.69
163.49
160.24
153.94
146.50
146.46
140.39
138.56
130.77
130.66
128.08
127.54
127.42
124.66
124.39
124.36
119.83
117.30
117.02
115.30
114.99
24.61
F
H3C
O
N
NH
NH
Cl
N
O
N
1f
210
200
190
180
170
160
150
140
130
120
110
100
90
80
70
60
50
40
30
20
10
0

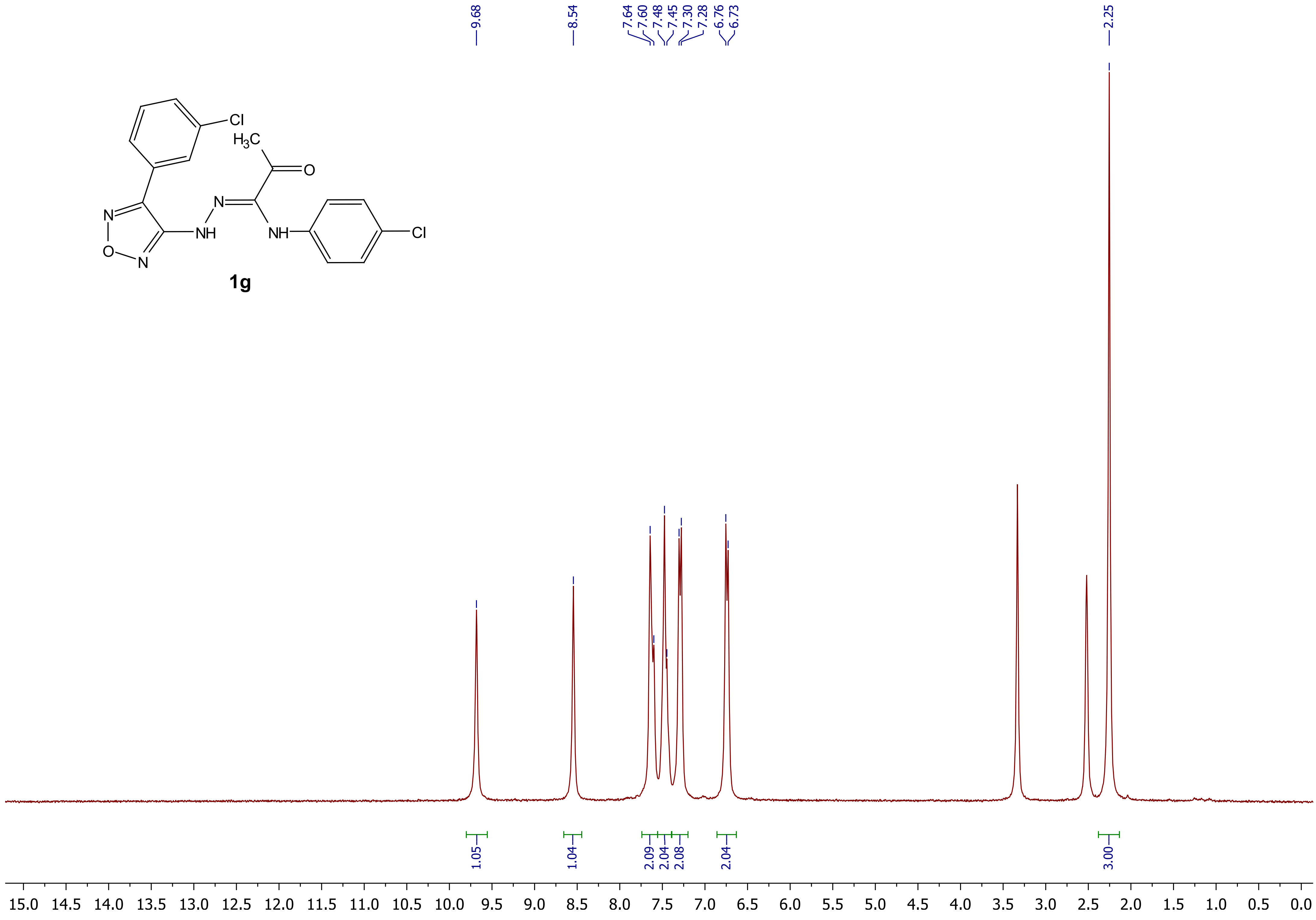
9.68
8.54
7.64
7.60
7.48
7.45
7.30
7.28
6.76
6.73
2.25
Cl
H3C
O
N
NH
NH
Cl
N
O
N
1g
1.05
1.04
2.09
2.04
2.08
2.04
3.00
15.0 14.5 14.0 13.5 13.0 12.5 12.0 11.5 11.0 10.5 10.0 9.5 9.0 8.5 8.0 7.5 7.0 6.5 6.0 5.5 5.0 4.5 4.0 3.5 3.0 2.5 2.0 1.5 1.0 0.5 0.0

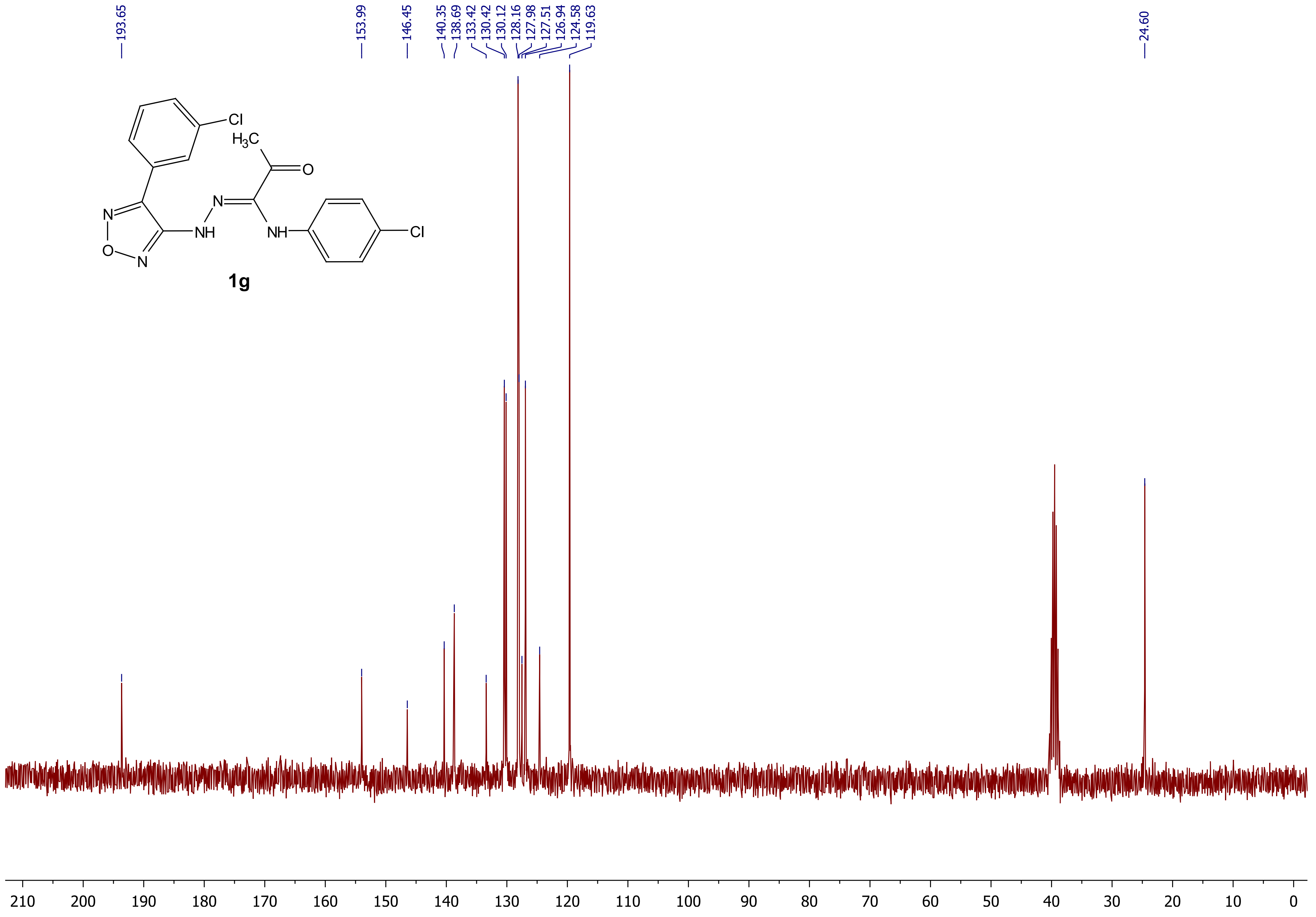
193.65
153.99
146.45
140.35
138.69
133.42
130.42
130.12
128.16
127.98
127.51
126.94
124.58
119.63
24.60
Cl
H3C
O
N
NH
NH
Cl
N
O
N
1g
210 200 190 180 170 160 150 140 130 120 110 100 90 80 70 60 50 40 30 20 10 0

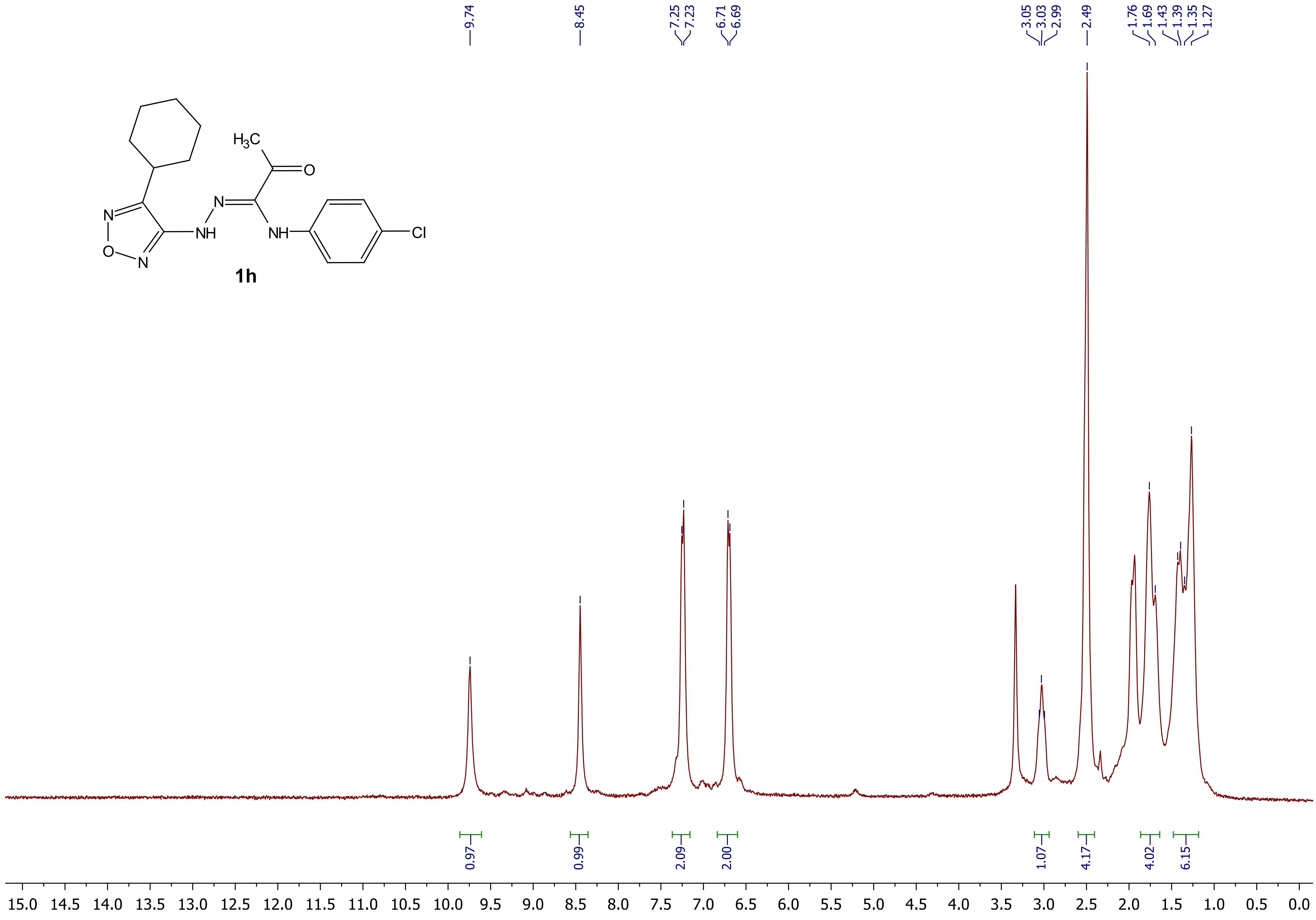

9.74
8.45
7.25
7.23
6.71
6.69
3.05
3.03
2.99
2.49
1.76
1.69
1.43
1.39
1.35
1.27
H3C
O
N
NH
NH
Cl
N
O
N
1h
0.97
0.99
2.09
2.00
1.07
4.17
4.02
6.15
15.0 14.5 14.0 13.5 13.0 12.5 12.0 11.5 11.0 10.5 10.0 9.5 9.0 8.5 8.0 7.5 7.0 6.5 6.0 5.5 5.0 4.5 4.0 3.5 3.0 2.5 2.0 1.5 1.0 0.5 0.0

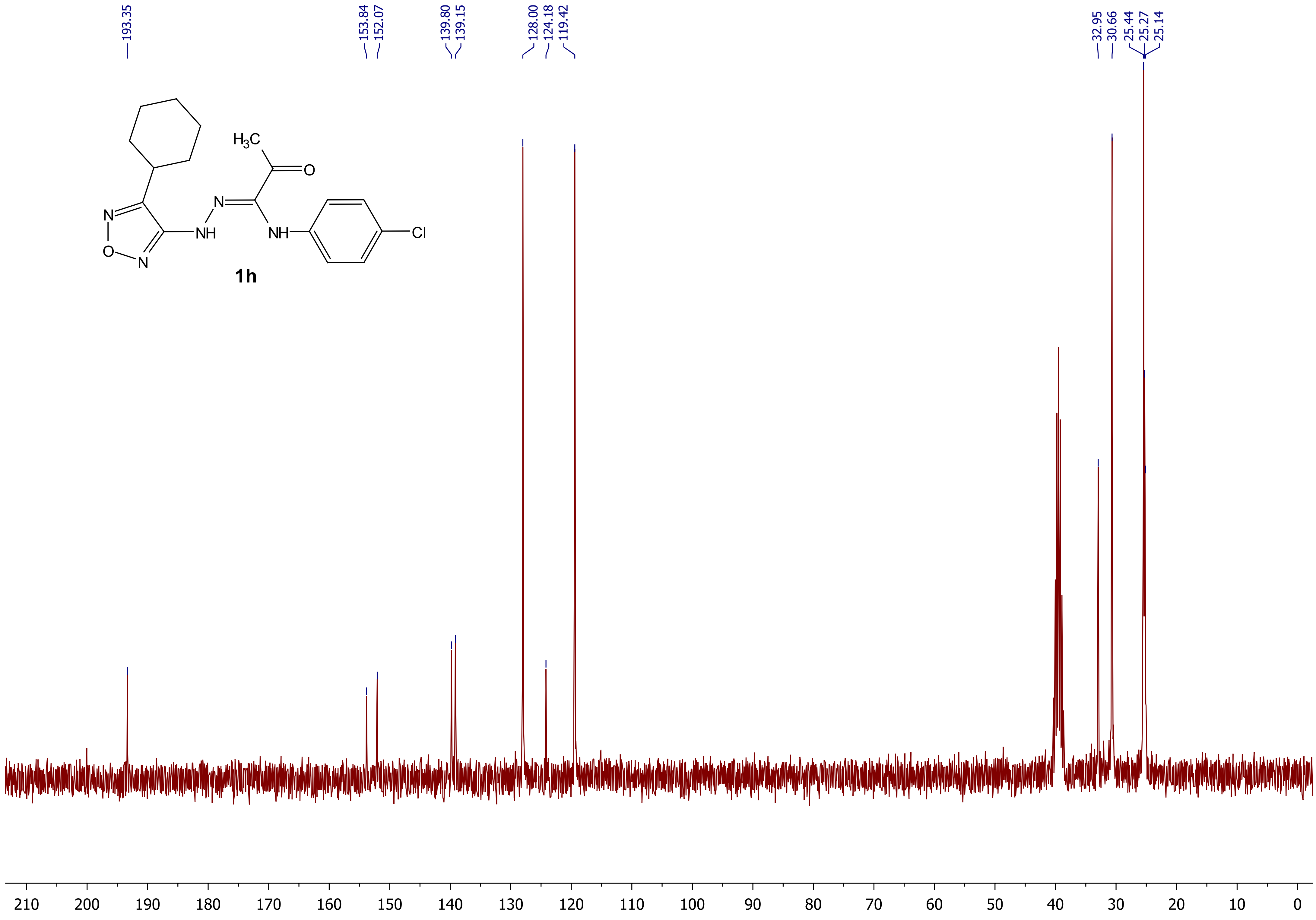
193.35
153.84
152.07
139.80
139.15
128.00
124.18
119.42
32.95
30.66
25.44
25.27
25.14
H3C
O
N
NH
NH
Cl
N
O
N
1h
210 200 190 180 170 160 150 140 130 120 110 100 90 80 70 60 50 40 30 20 10 0

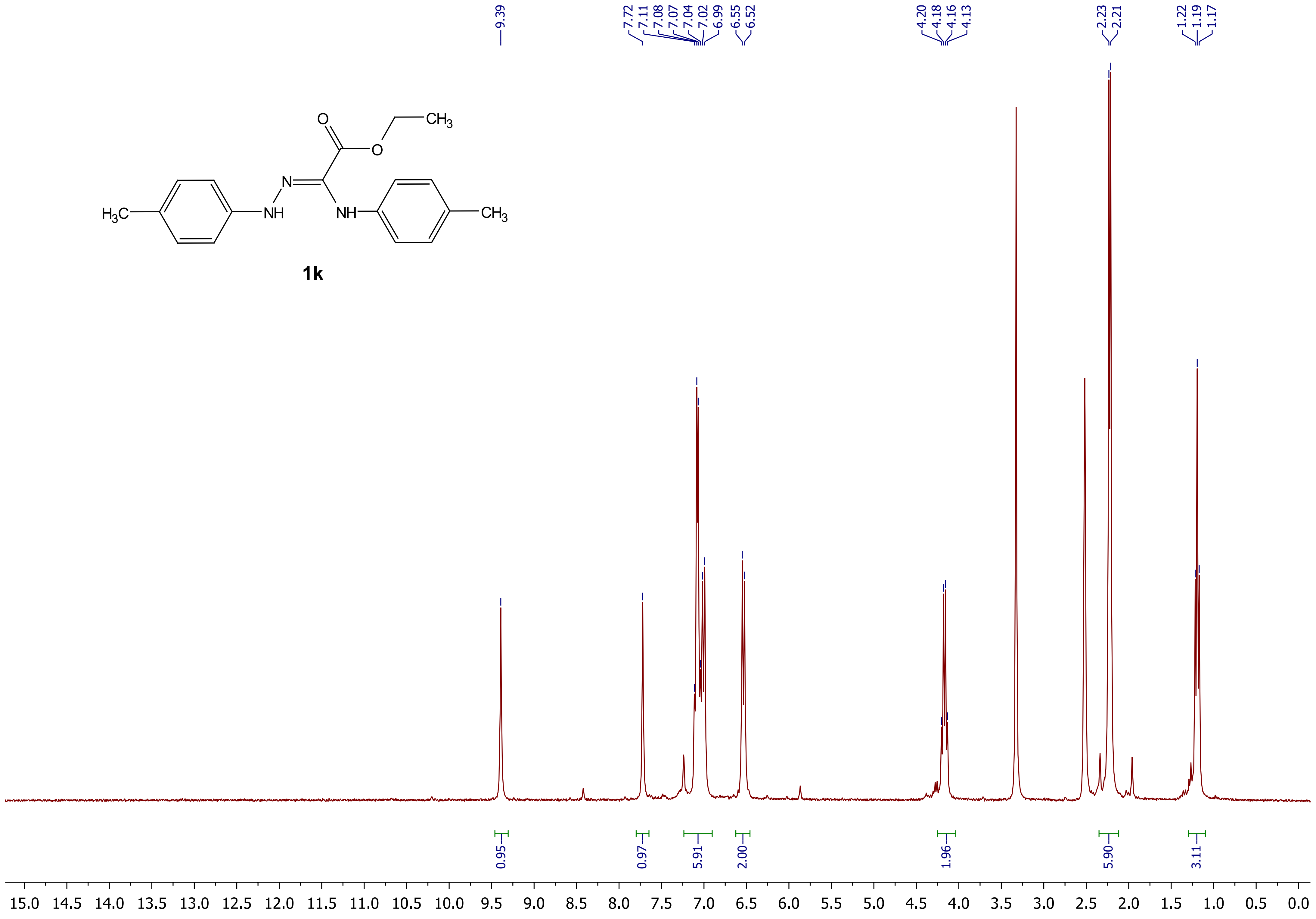
9.39
7.72
7.11
7.08
7.07
7.04
7.02
6.99
6.55
6.52
4.20
4.18
4.16
4.13
2.23
2.21
1.22
1.19
1.17
O
CH3
O
N
H3C
NH
NH
CH3
1k
0.95
0.97
5.91
2.00
1.96
5.90
3.11
15.0 14.5 14.0 13.5 13.0 12.5 12.0 11.5 11.0 10.5 10.0 9.5 9.0 8.5 8.0 7.5 7.0 6.5 6.0 5.5 5.0 4.5 4.0 3.5 3.0 2.5 2.0 1.5 1.0 0.5 0.0

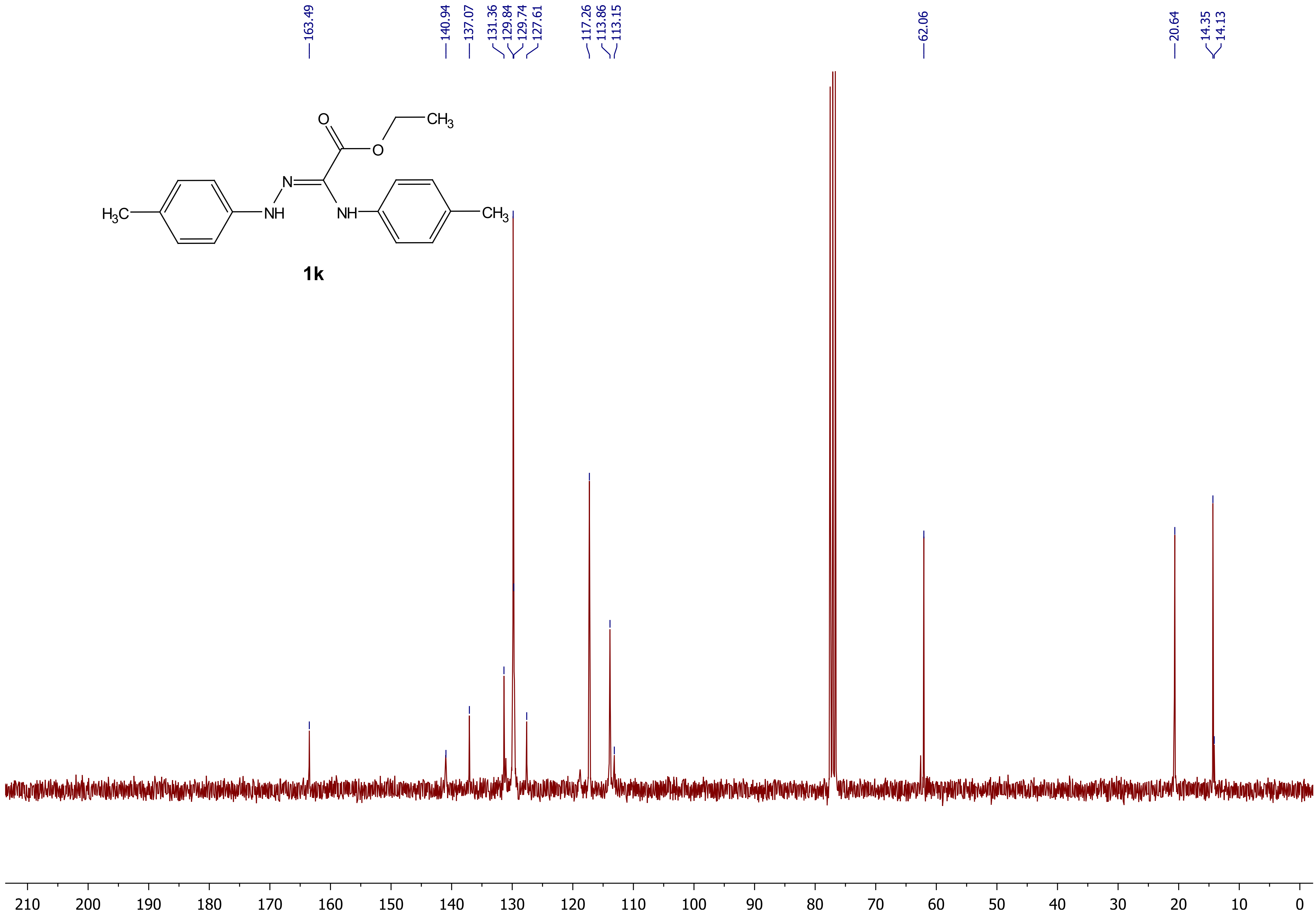

163.49
140.94
137.07
131.36
129.84
129.74
127.61
117.26
113.86
113.15
62.06
20.64
14.35
14.13
O
O
CH3
N
H3C
NH
NH
CH3
1k
210
200
190
180
170
160
150
140
130
120
110
100
90
80
70
60
50
40
30
20
10
0

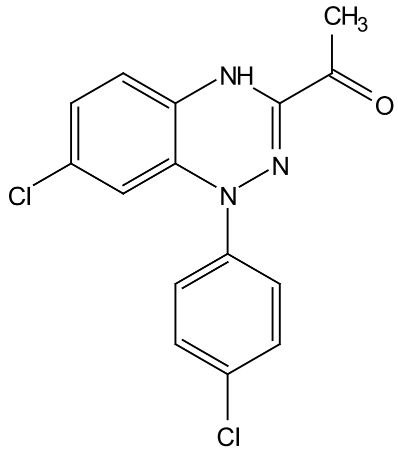
CH3
NH
O
N
Cl
N
Cl

5a

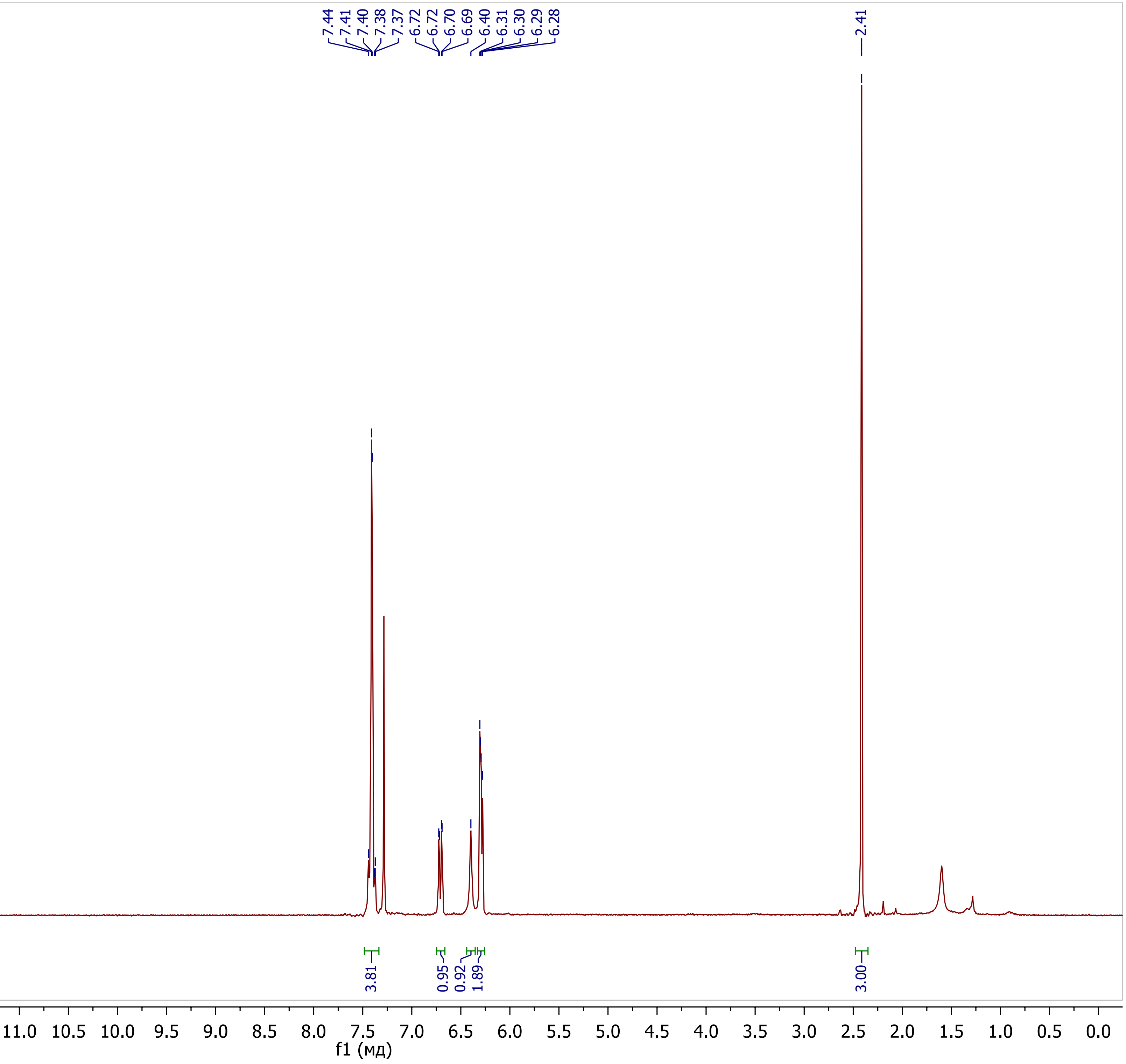
7.44
7.41
7.40
7.38
7.37
6.72
6.72
6.70
6.69
6.40
6.31
6.30
6.29
6.28
2.41
3.81
0.95
0.92
1.89
3.00
15.0 14.5 14.0 13.5 13.0 12.5 12.0 11.5 11.0 10.5 10.0 9.5 9.0 8.5 8.0 7.5 7.0 6.5 6.0 5.5 5.0 4.5 4.0 3.5 3.0 2.5 2.0 1.5 1.0 0.5 0.0
f1 (мд)

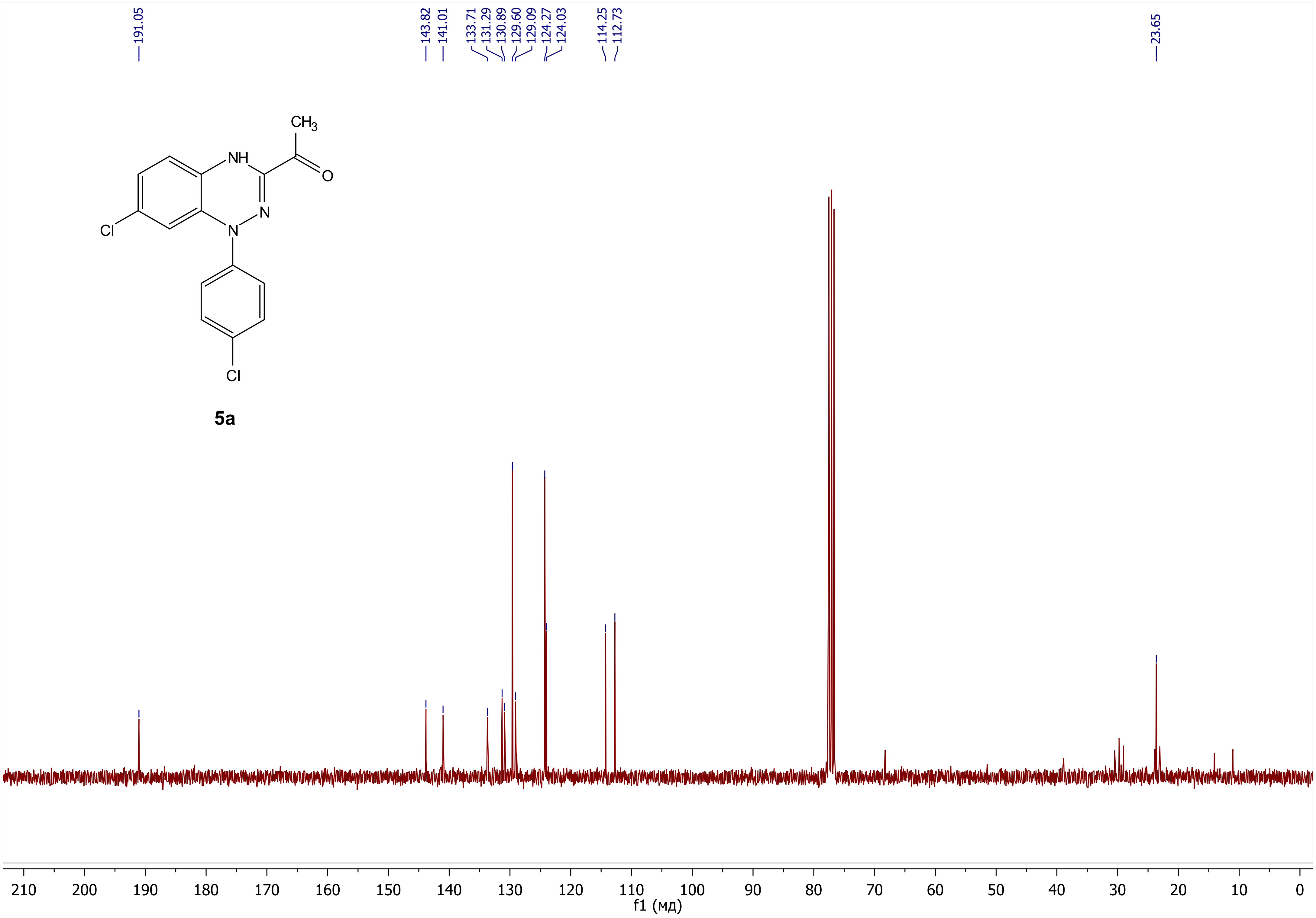

191.05
143.82
141.01
133.71
131.29
130.89
129.60
129.09
124.27
124.03
114.25
112.73
23.65
CH3
NH
O
N
Cl
N
Cl
5a
210 200 190 180 170 160 150 140 130 120 110 100 90 80 70 60 50 40 30 20 10 0
f1 (мд)

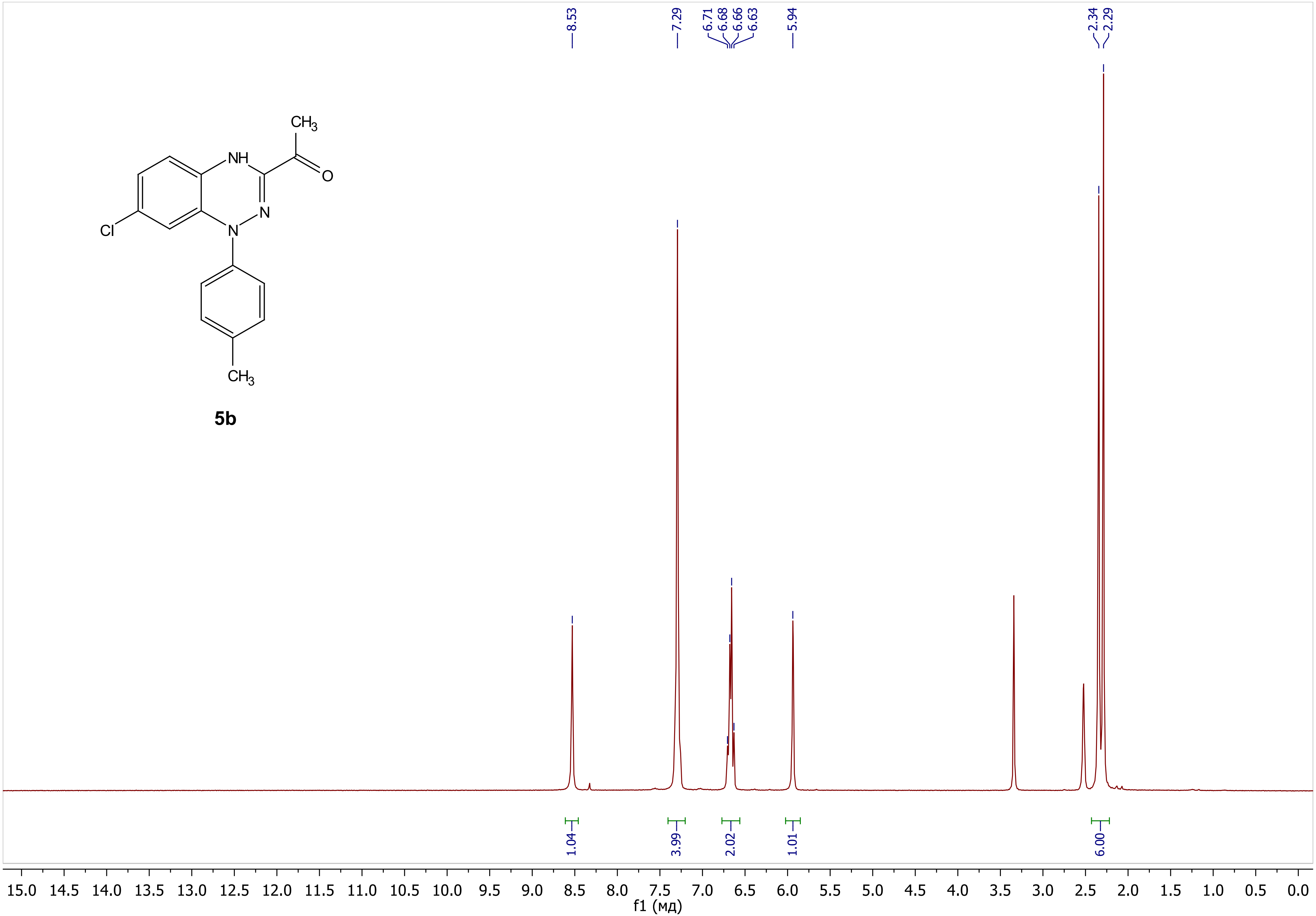
8.53
7.29
6.71
6.68
6.66
6.63
5.94
2.34
2.29
CH3
NH
O
N
Cl
N
CH3
5b
1.04
3.99
2.02
1.01
6.00
15.0 14.5 14.0 13.5 13.0 12.5 12.0 11.5 11.0 10.5 10.0 9.5 9.0 8.5 8.0 7.5 7.0 6.5 6.0 5.5 5.0 4.5 4.0 3.5 3.0 2.5 2.0 1.5 1.0 0.5 0.0
f1 (мд)

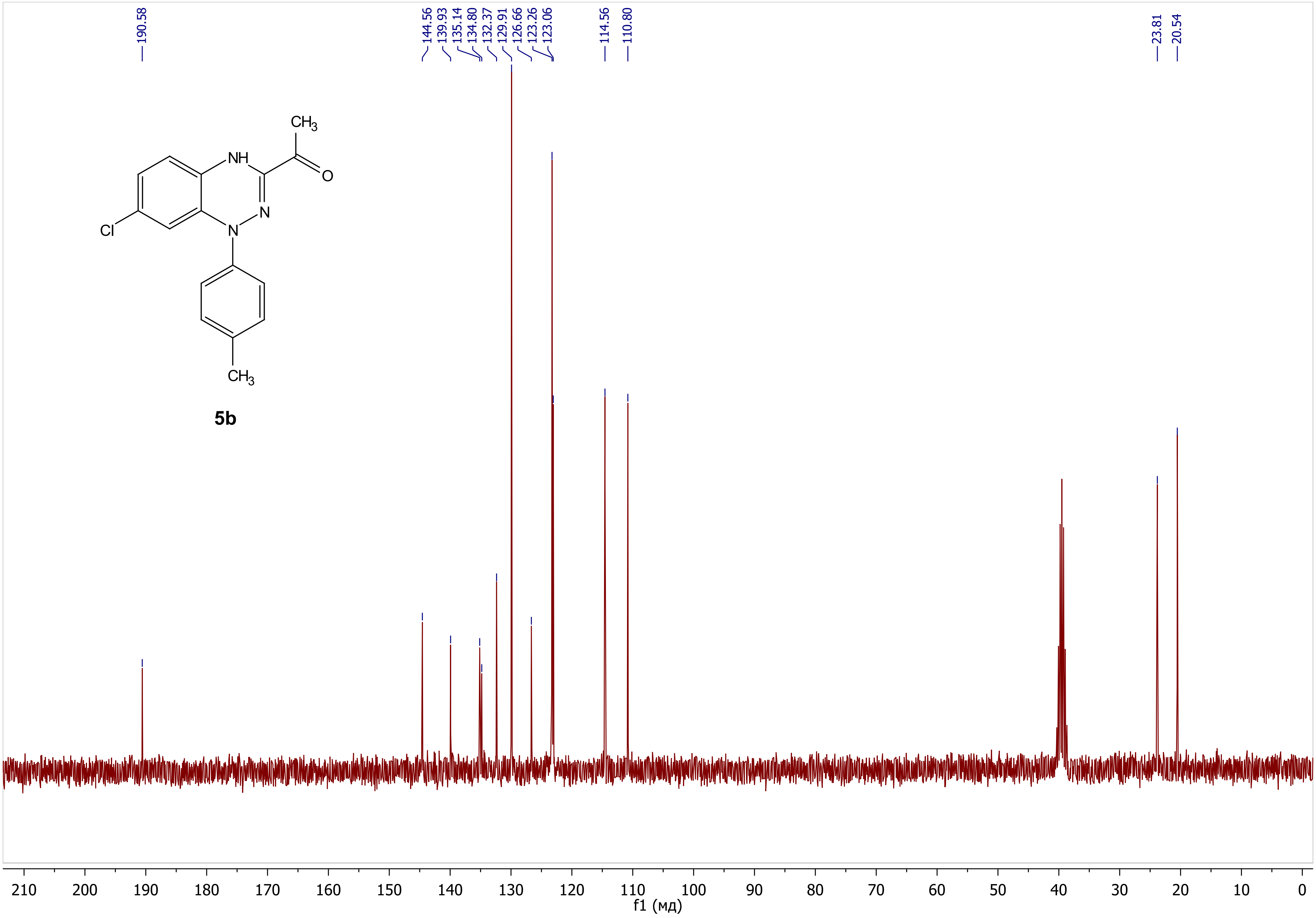
190.58
144.56
139.93
135.14
134.80
132.37
129.91
126.66
123.26
123.06
114.56
110.80
23.81
20.54
CH3
NH
O
N
Cl
N
CH3
5b
210 200 190 180 170 160 150 140 130 120 110 100 90 80 70 60 50 40 30 20 10 0
f1 (мд)

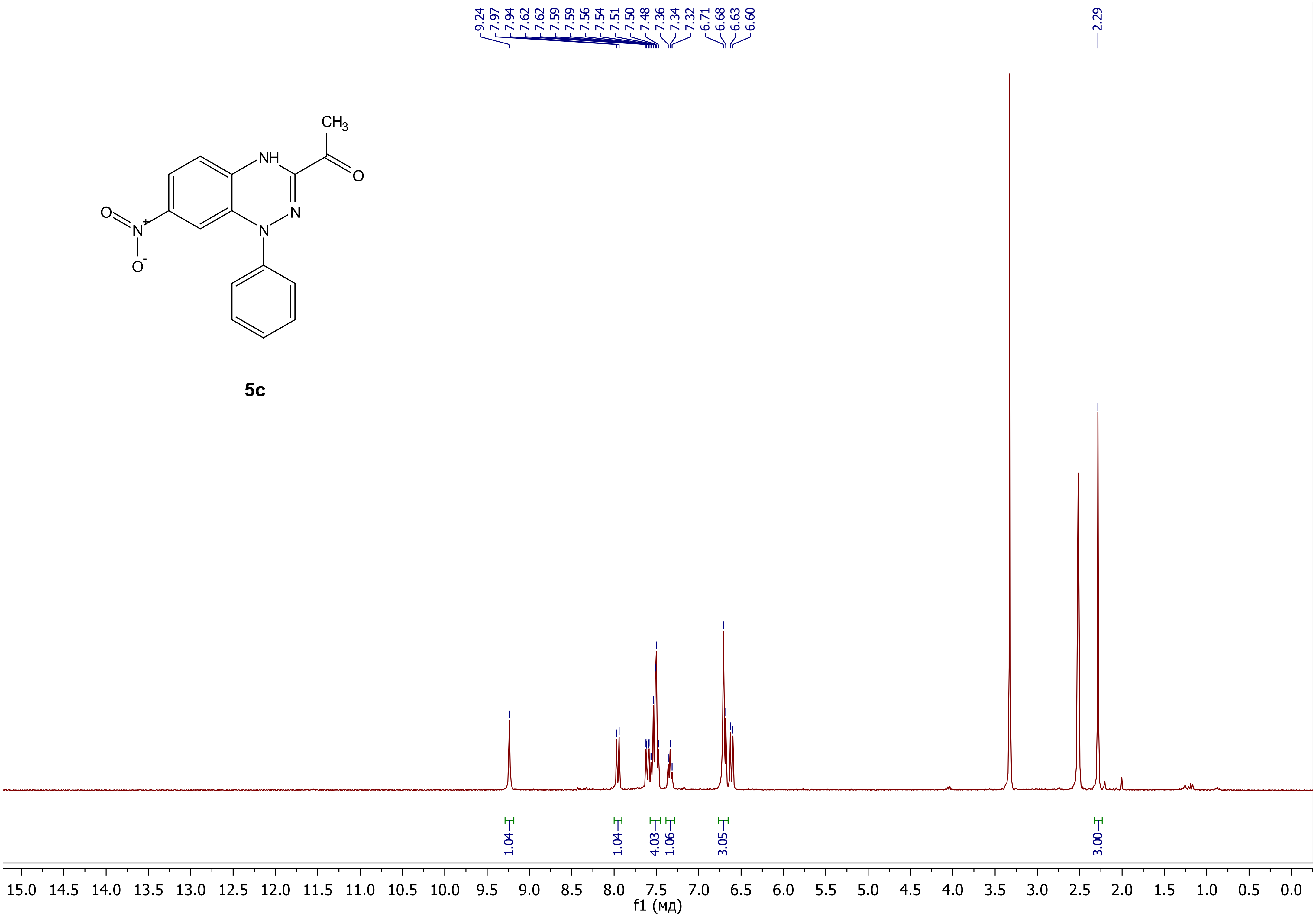
9.24
7.97
7.94
7.62
7.62
7.59
7.59
7.56
7.54
7.51
7.50
7.48
7.36
7.34
7.32
6.71
6.68
6.63
6.60
2.29
CH3
NH
O
N
N
O
N+
O-
5c
1.04
1.04
4.03
1.06
3.05
3.00
15.0 14.5 14.0 13.5 13.0 12.5 12.0 11.5 11.0 10.5 10.0 9.5 9.0 8.5 8.0 7.5 7.0 6.5 6.0 5.5 5.0 4.5 4.0 3.5 3.0 2.5 2.0 1.5 1.0 0.5 0.0
f1 (мд)

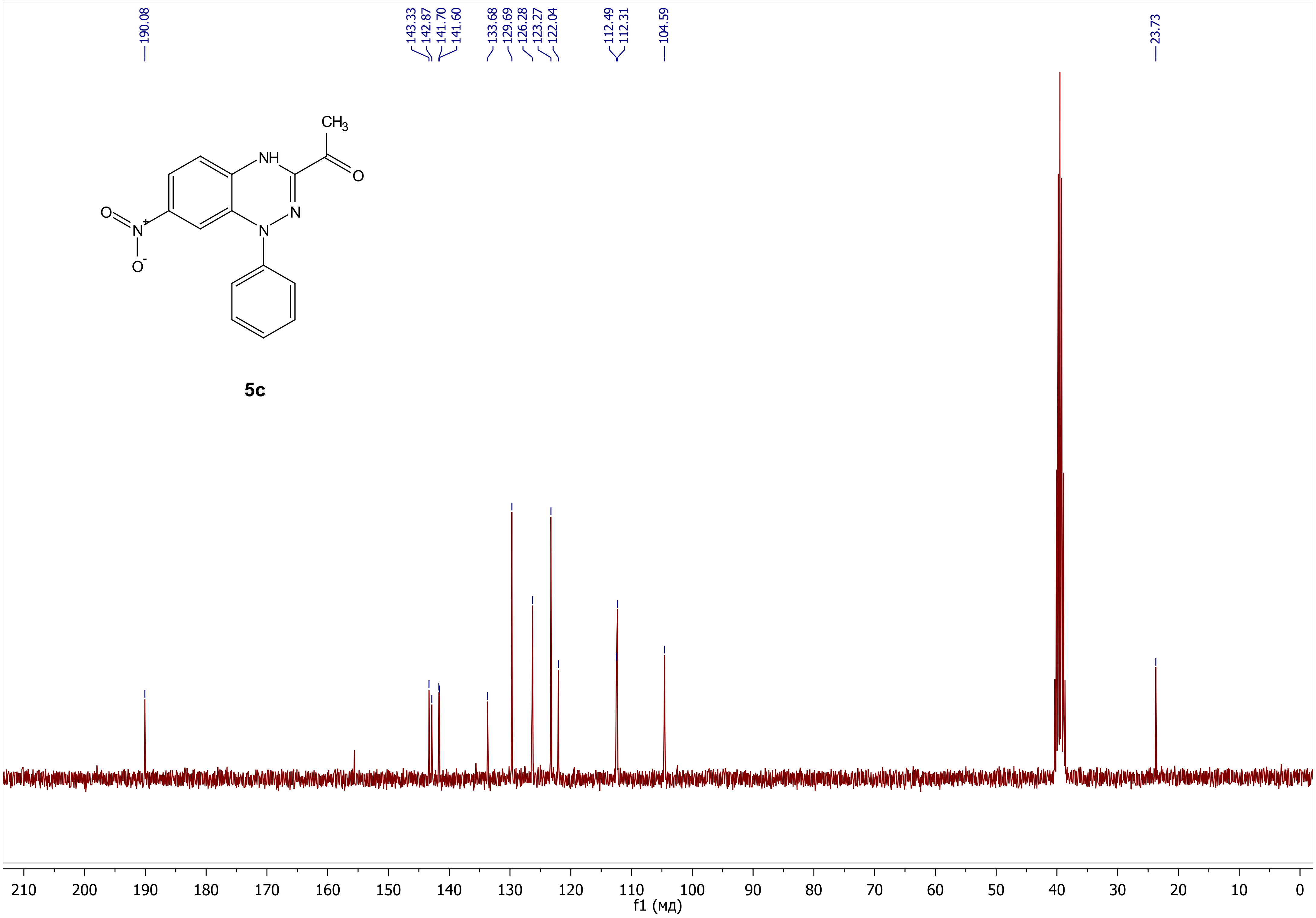
190.08
143.33
142.87
141.70
141.60
133.68
129.69
126.28
123.27
122.04
112.49
112.31
104.59
23.73
CH3
NH
O
N
N
O
N+
O-
5c
210 200 190 180 170 160 150 140 130 120 110 100 90 80 70 60 50 40 30 20 10 0
f1 (мд)

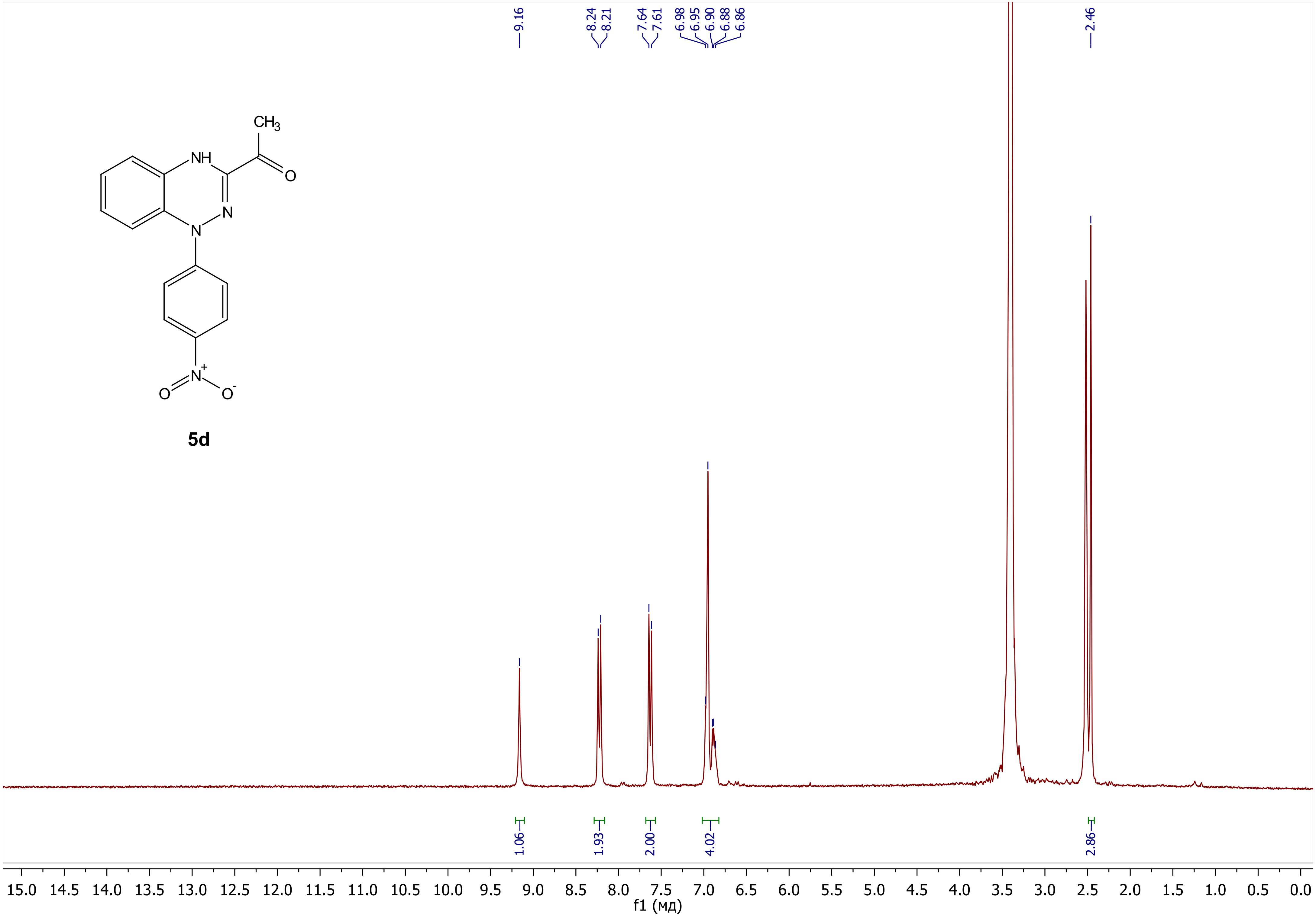

9.16
8.24
8.21
7.64
7.61
6.98
6.95
6.90
6.88
6.86
2.46
CH_3
NH
N
N
N^+
O
O^-
5d
1.06
1.93
2.00
4.02
2.86
15.0 14.5 14.0 13.5 13.0 12.5 12.0 11.5 11.0 10.5 10.0 9.5 9.0 8.5 8.0 7.5 7.0 6.5 6.0 5.5 5.0 4.5 4.0 3.5 3.0 2.5 2.0 1.5 1.0 0.5 0.0
f1 (мд)

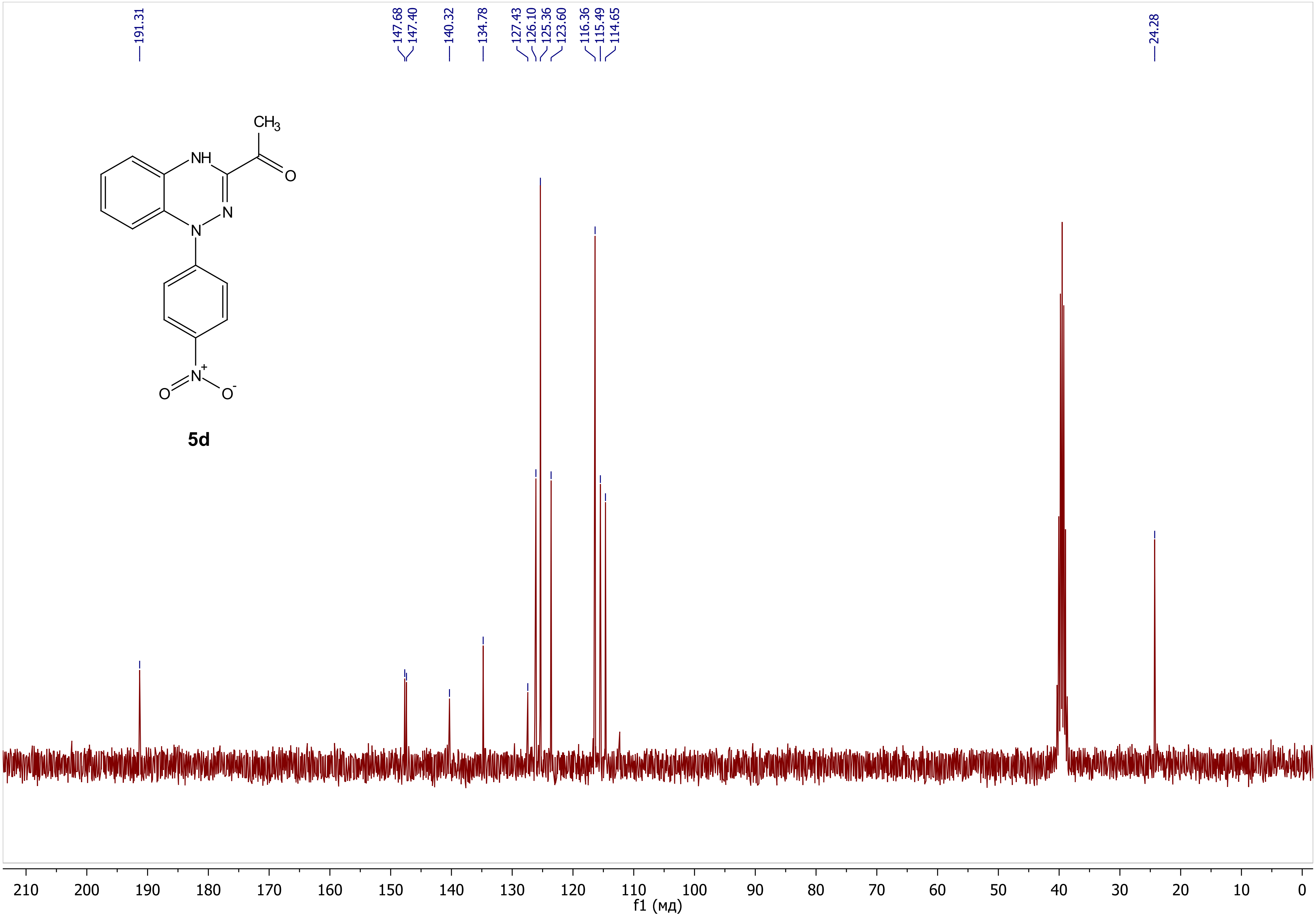
191.31
147.68
147.40
140.32
134.78
127.43
126.10
125.36
123.60
116.36
115.49
114.65
24.28
CH3
NH
N
N
N+
O
O-
5d
210 200 190 180 170 160 150 140 130 120 110 100 90 80 70 60 50 40 30 20 10 0
f1 (мд)

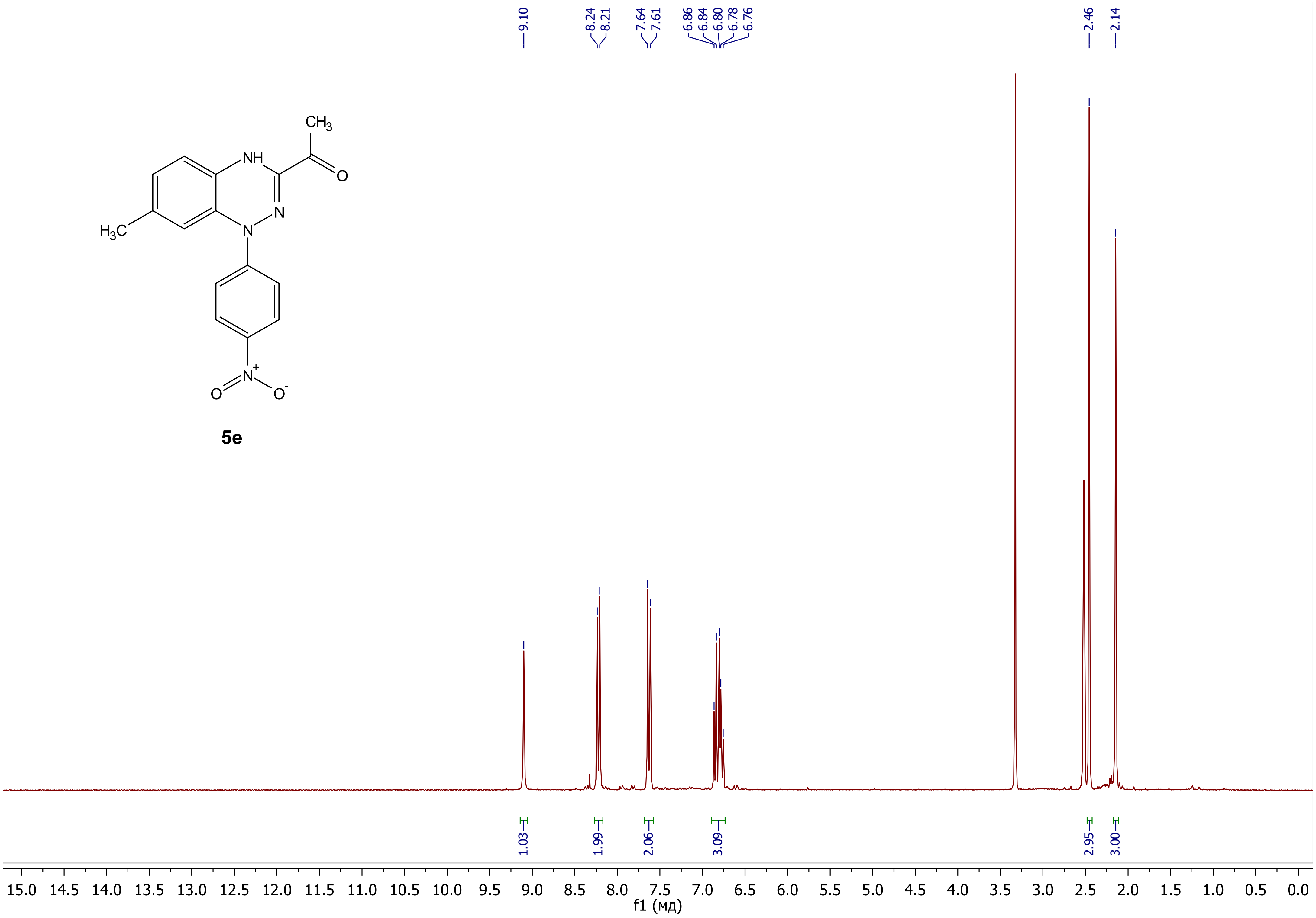
9.10
8.24
8.21
7.64
7.61
6.86
6.84
6.80
6.78
6.76
2.46
2.14
CH3
NH
O
N
N
H3C
N+
O
O-
5e
1.03
1.99
2.06
3.09
2.95
3.00
15.0 14.5 14.0 13.5 13.0 12.5 12.0 11.5 11.0 10.5 10.0 9.5 9.0 8.5 8.0 7.5 7.0 6.5 6.0 5.5 5.0 4.5 4.0 3.5 3.0 2.5 2.0 1.5 1.0 0.5 0.0
f1 (мд)

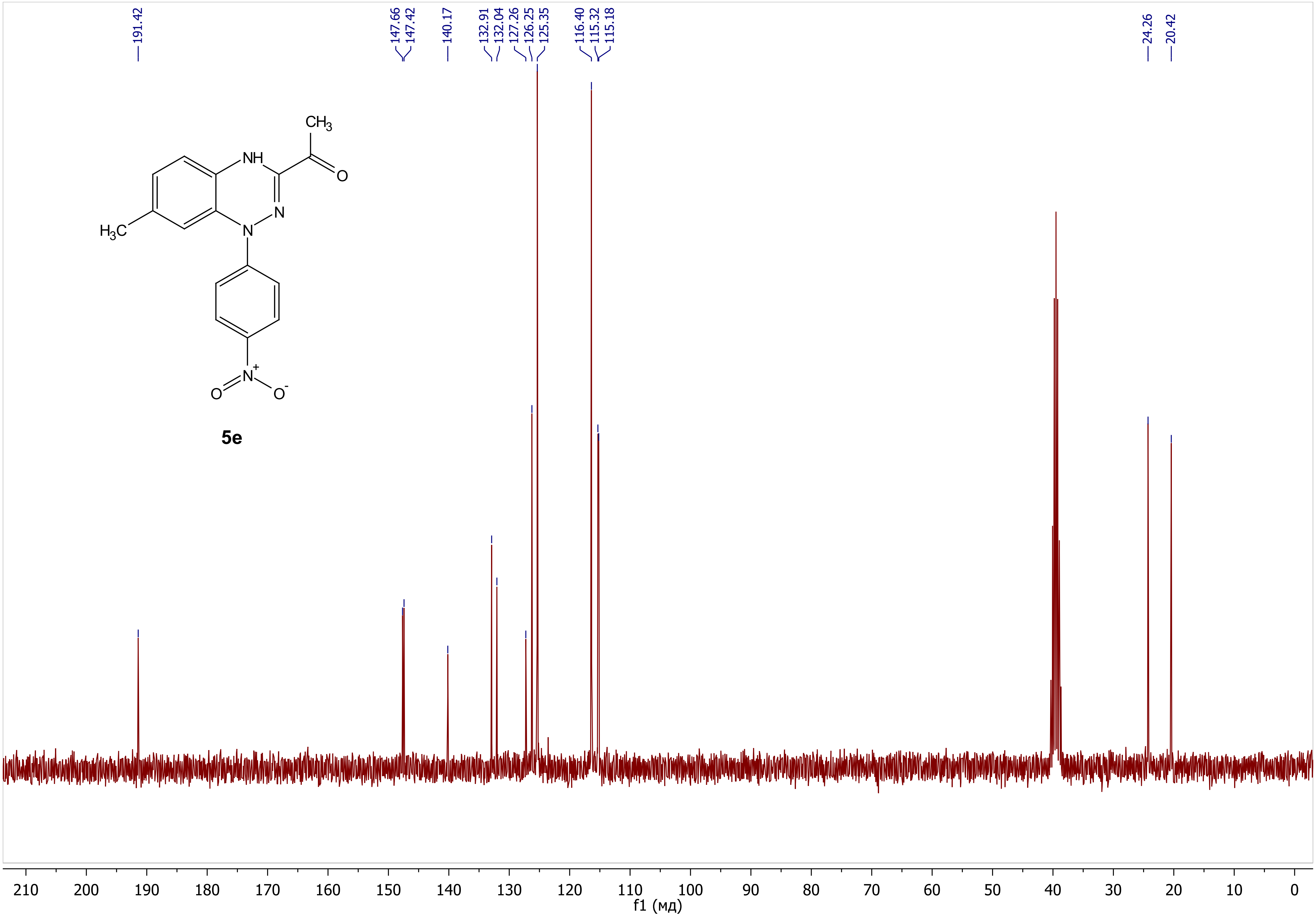

191.42
147.66
147.42
140.17
132.91
132.04
127.26
126.25
125.35
116.40
115.32
115.18
24.26
20.42
CH3
NH
O
N
H3C
N
N+
O
O-
5e
210 200 190 180 170 160 150 140 130 120 110 100 90 80 70 60 50 40 30 20 10 0
f1 (мд)

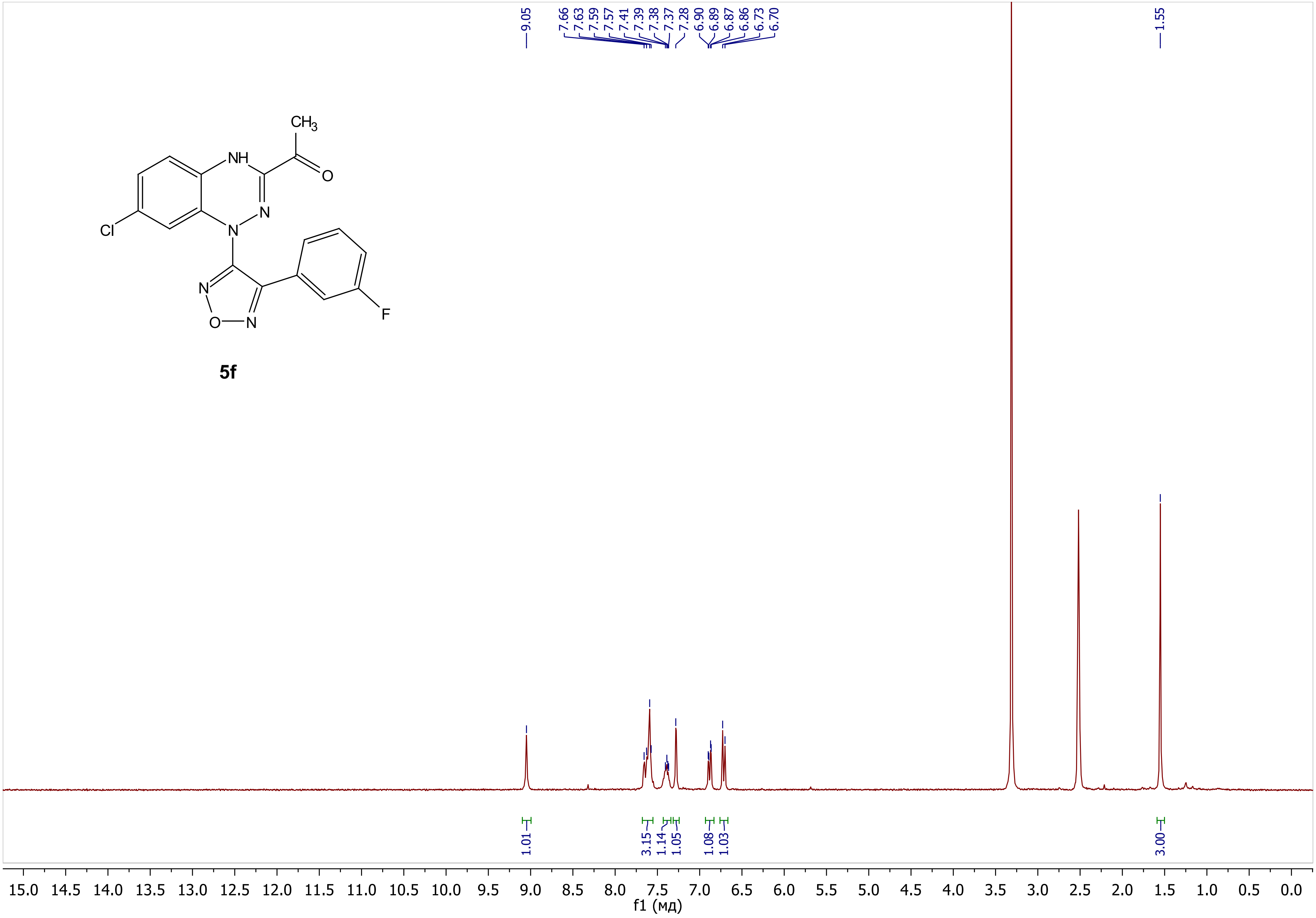
9.05
7.66 7.63 7.59 7.57 7.41 7.39 7.38 7.37 7.28 6.90 6.89 6.87 6.86 6.73 6.70
1.55
CH3
NH
O
N
N
Cl
N
O
N
F
5f
1.01
3.15
1.14
1.05
1.08
1.03
3.00
15.0 14.5 14.0 13.5 13.0 12.5 12.0 11.5 11.0 10.5 10.0 9.5 9.0 8.5 8.0 7.5 7.0 6.5 6.0 5.5 5.0 4.5 4.0 3.5 3.0 2.5 2.0 1.5 1.0 0.5 0.0
f1 (мд)

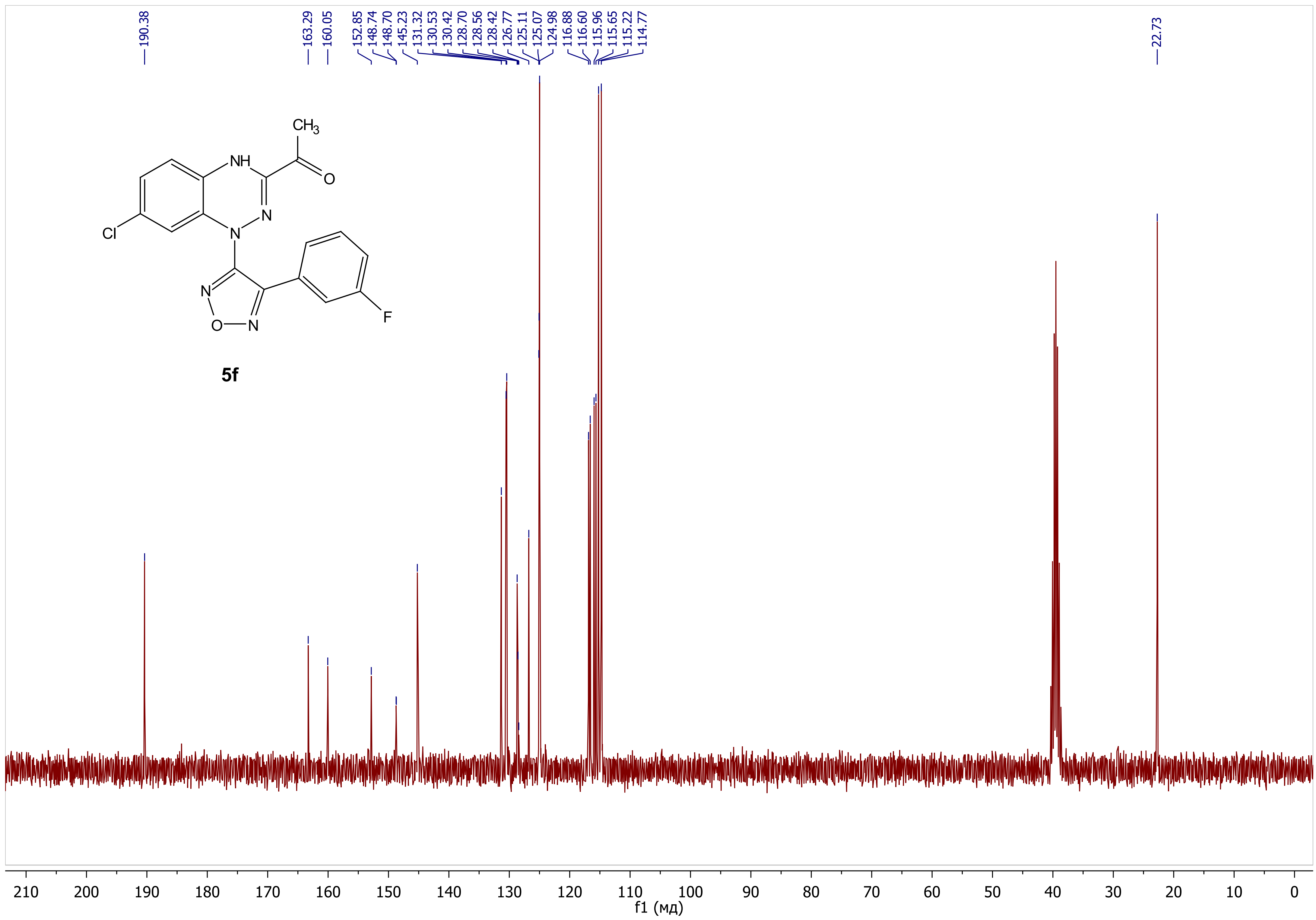

190.38
163.29
160.05
152.85
148.74
148.70
145.23
131.32
130.53
130.42
128.70
128.56
128.42
126.77
125.11
125.07
124.98
116.88
116.60
115.96
115.65
115.22
114.77
22.73
CH3
NH
O
N
Cl
N
N
O
N
F
5f
f1 (мд)

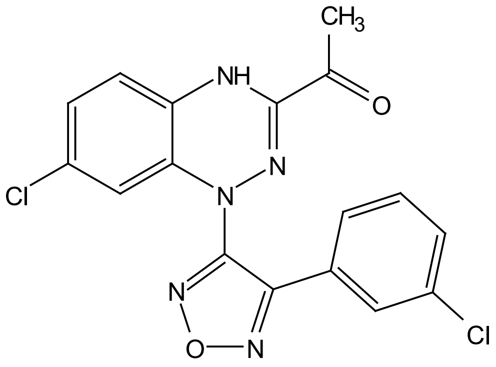

5g

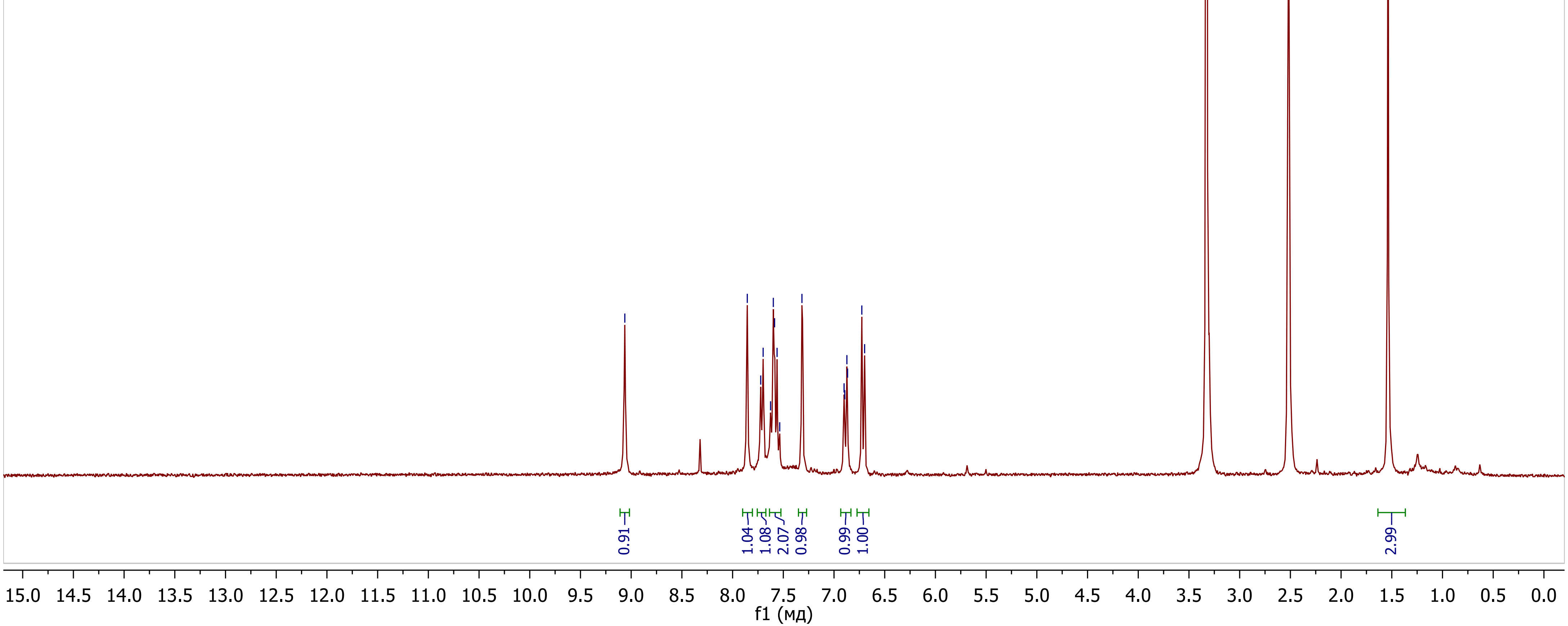
9.06
7.86
7.72
7.70
7.63
7.60
7.59
7.56
7.54
7.32
6.90
6.89
6.87
6.87
6.73
6.70
1.54
0.91
1.04
1.08
2.07
0.98
0.99
1.00
2.99
15.0 14.5 14.0 13.5 13.0 12.5 12.0 11.5 11.0 10.5 10.0 9.5 9.0 8.5 8.0 7.5 7.0 6.5 6.0 5.5 5.0 4.5 4.0 3.5 3.0 2.5 2.0 1.5 1.0 0.5 0.0
f1 (мд)

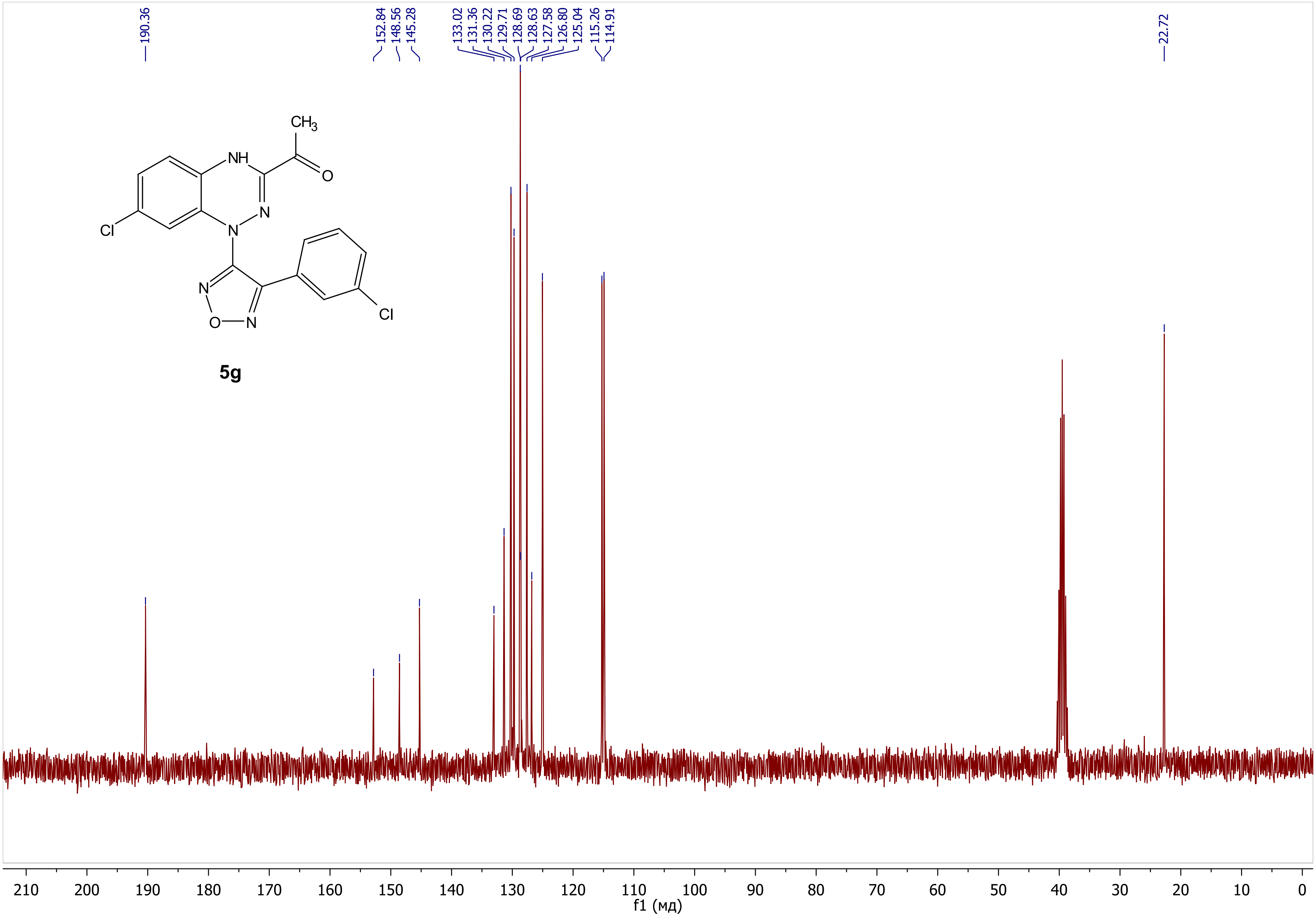
190.36
152.84
148.56
145.28
133.02
131.36
130.22
129.71
128.69
128.63
127.58
126.80
125.04
115.26
114.91
22.72
CH3
NH
O
N
Cl
N
N
O
N
Cl
5g
210
200
190
180
170
160
150
140
130
120
110
100
90
80
70
60
50
40
30
20
10
0
f1 (мд)

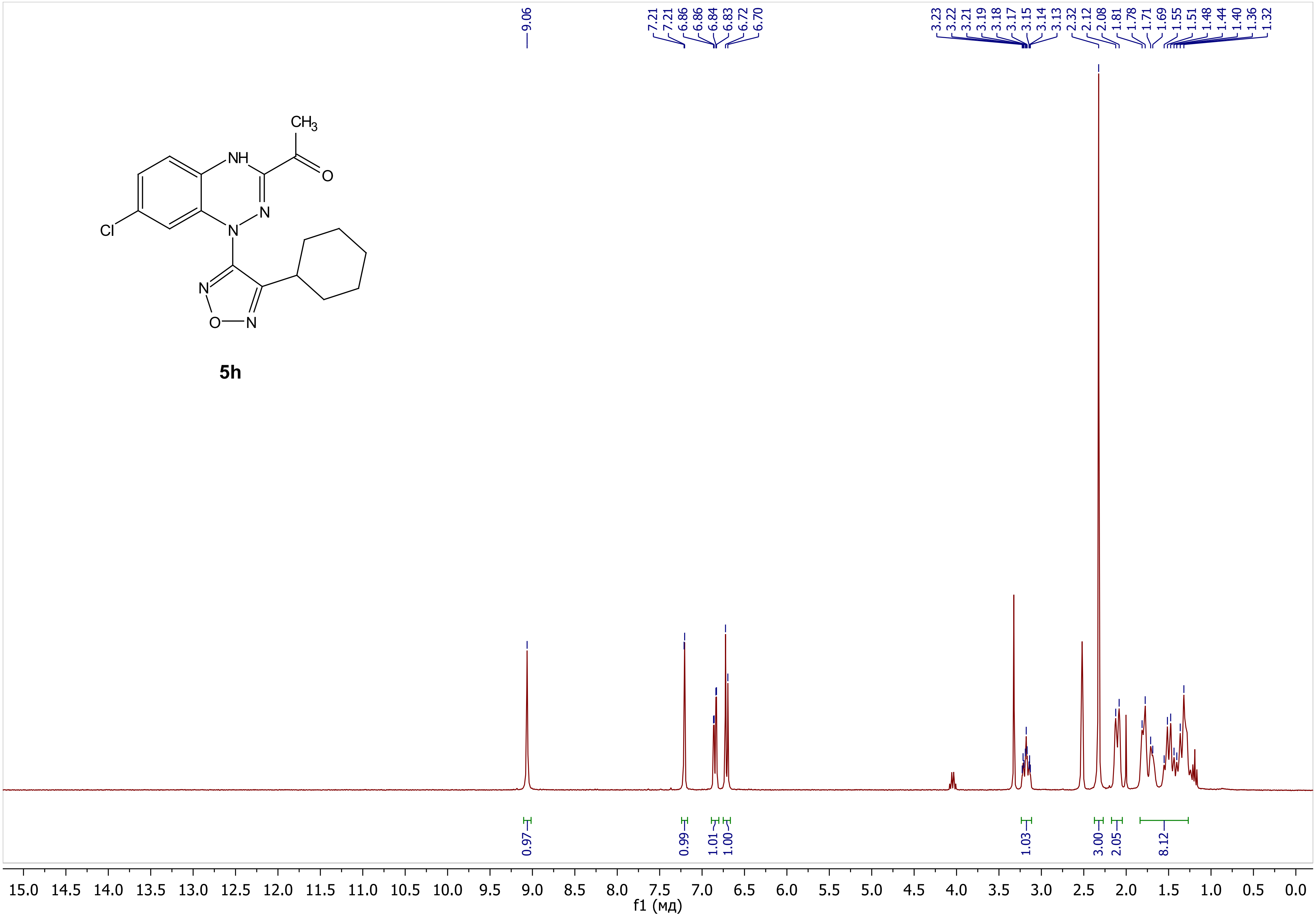
CH3
NH
O
N
Cl
N
N
O
N
9.06
7.21
7.21
6.86
6.86
6.84
6.83
6.72
6.70
3.23
3.22
3.21
3.19
3.18
3.17
3.15
3.14
3.13
2.32
2.12
2.08
1.81
1.78
1.71
1.69
1.55
1.51
1.48
1.44
1.40
1.36
1.32
0.97
0.99
1.01
1.00
1.03
3.00
2.05
8.12
15.0 14.5 14.0 13.5 13.0 12.5 12.0 11.5 11.0 10.5 10.0 9.5 9.0 8.5 8.0 7.5 7.0 6.5 6.0 5.5 5.0 4.5 4.0 3.5 3.0 2.5 2.0 1.5 1.0 0.5 0.0
f1 (мд)

5h

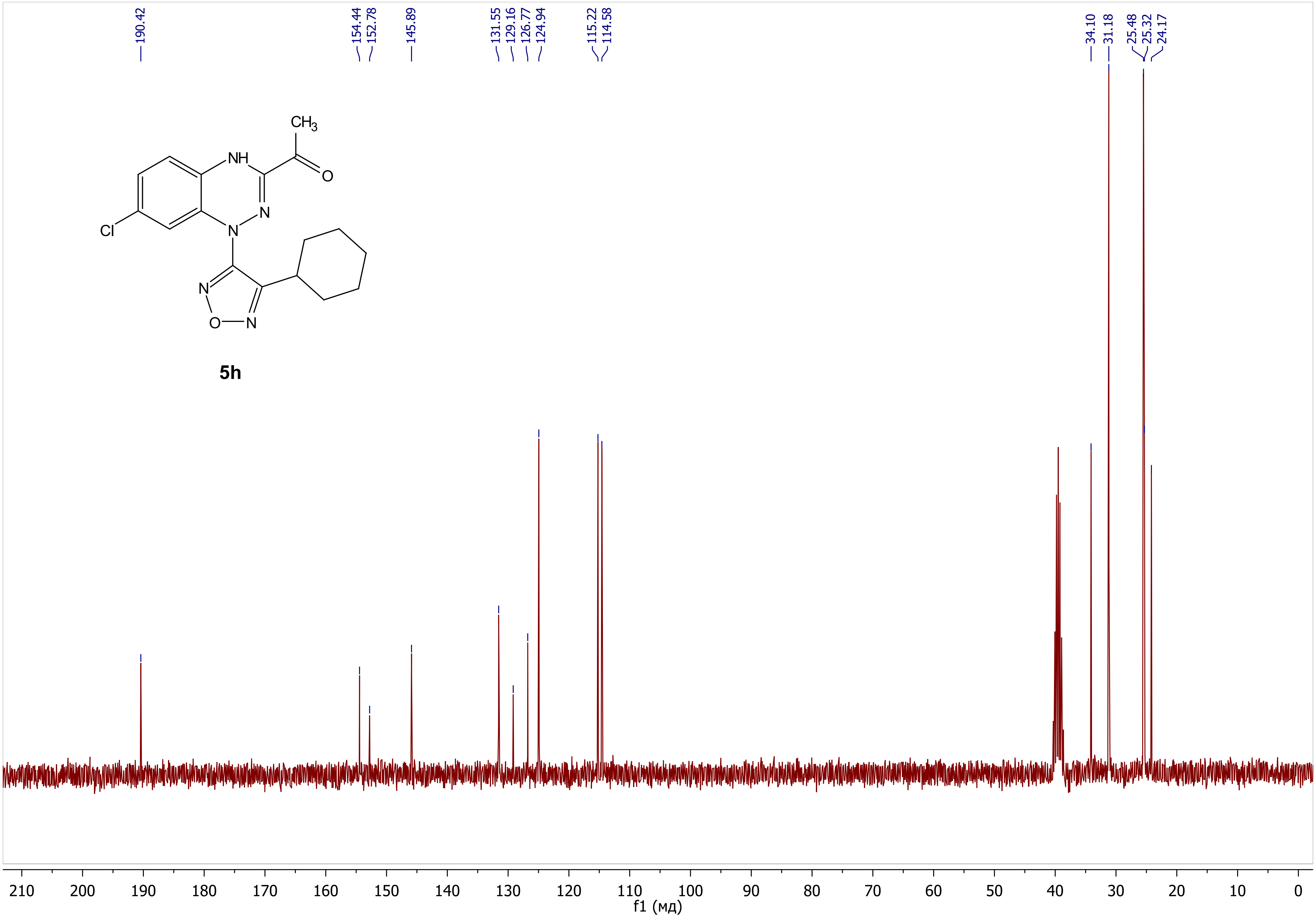
190.42
154.44
152.78
145.89
131.55
129.16
126.77
124.94
115.22
114.58
34.10
31.18
25.48
25.32
24.17
CH3
NH
O
N
Cl
N
N
O
N
5h
210
200
190
180
170
160
150
140
130
120
110
100
90
80
70
60
50
40
30
20
10
0
f1 (мд)

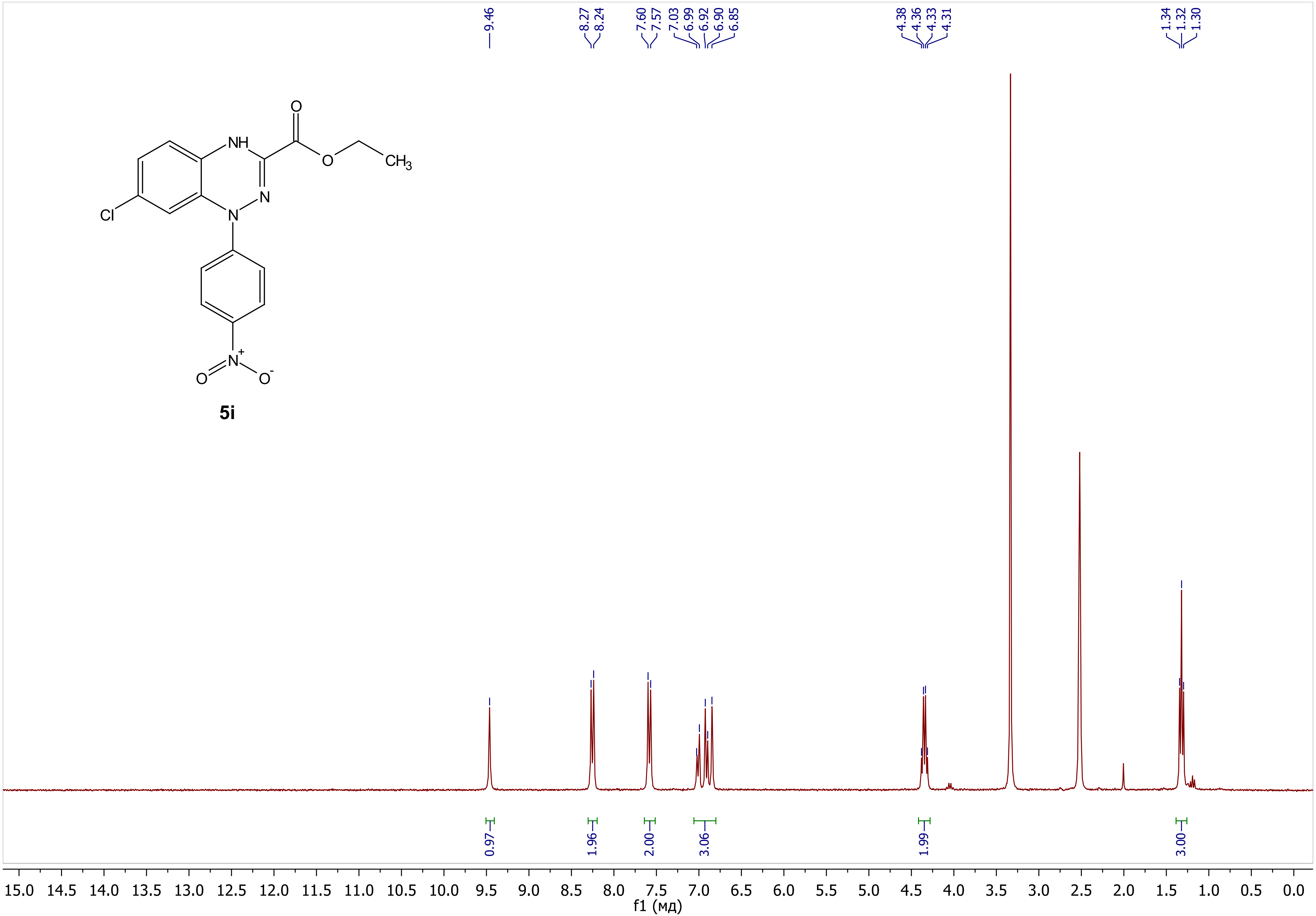

NH
O
CH3
N
N
Cl
O
N+
O-
5i
9.46
8.27
8.24
7.60
7.57
7.03
6.99
6.92
6.90
6.85
4.38
4.36
4.33
4.31
1.34
1.32
1.30
0.97
1.96
2.00
3.06
1.99
3.00
15.0 14.5 14.0 13.5 13.0 12.5 12.0 11.5 11.0 10.5 10.0 9.5 9.0 8.5 8.0 7.5 7.0 6.5 6.0 5.5 5.0 4.5 4.0 3.5 3.0 2.5 2.0 1.5 1.0 0.5 0.0
f1 (мд)

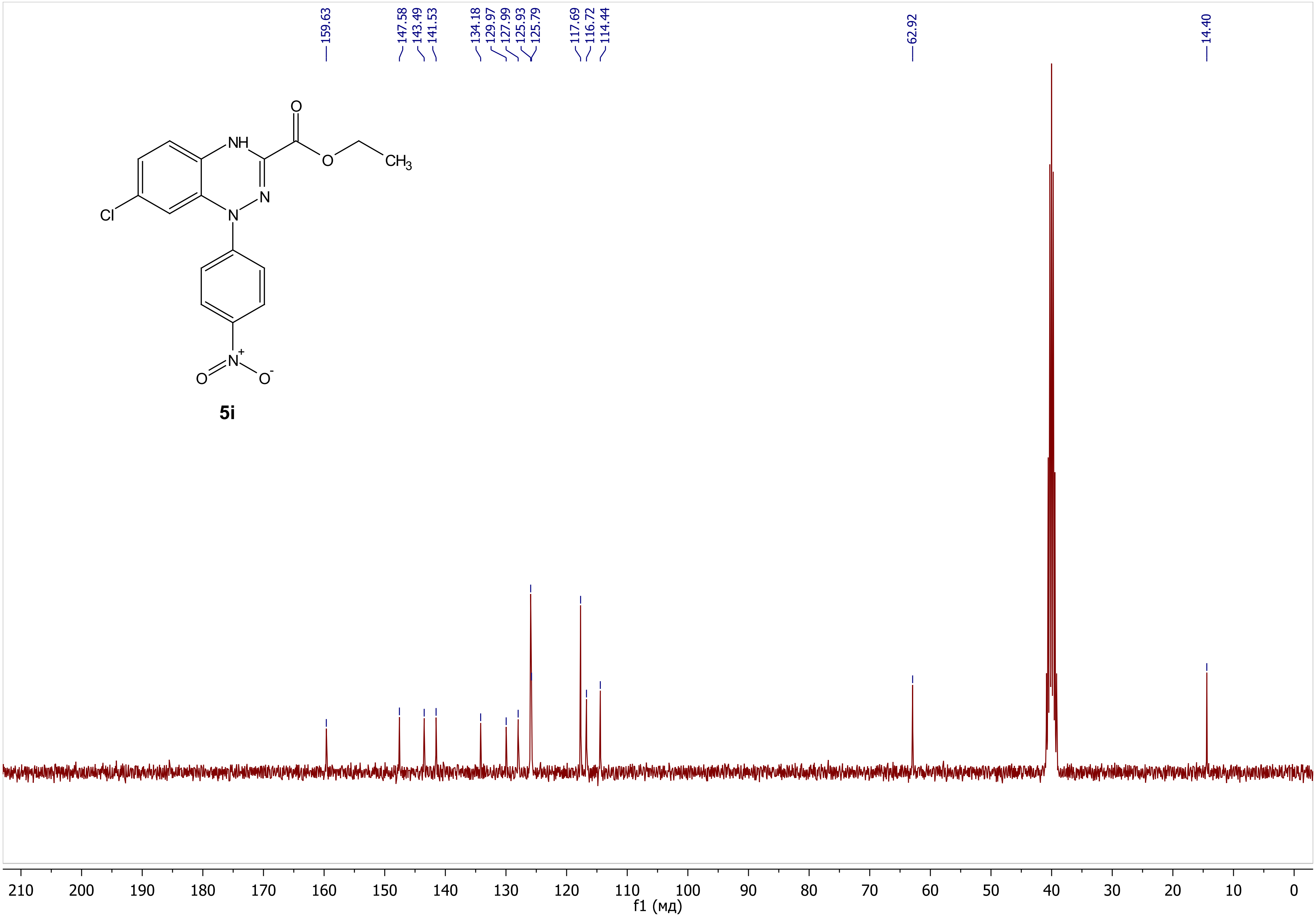
159.63
147.58
143.49
141.53
134.18
129.97
127.99
125.93
125.79
117.69
116.72
114.44
62.92
14.40
NH
O
O
CH3
N
N
Cl
N+
O
O-
5i
210 200 190 180 170 160 150 140 130 120 110 100 90 80 70 60 50 40 30 20 10 0
f1 (мд)

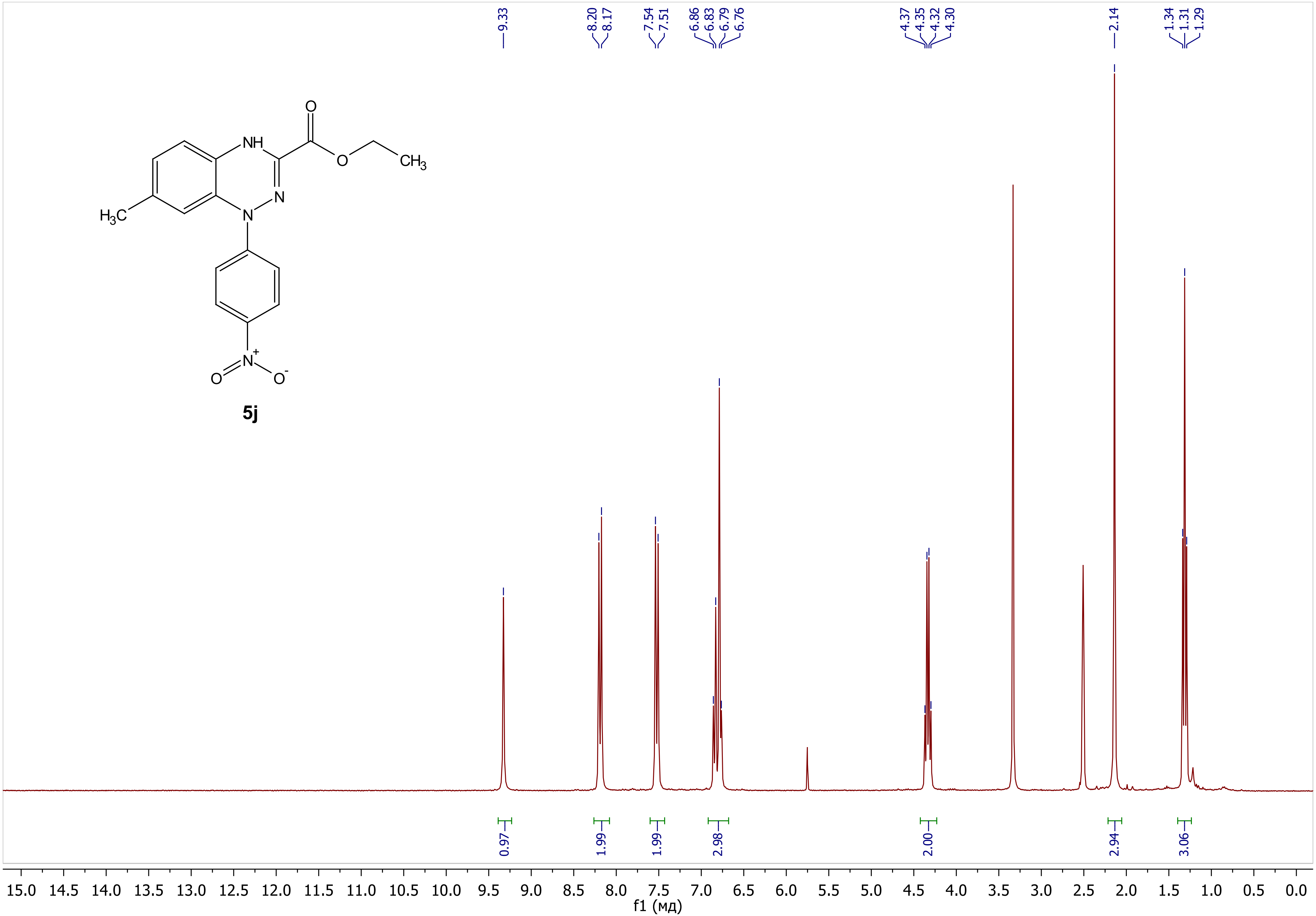
9.33
8.20
8.17
7.54
7.51
6.86
6.83
6.79
6.76
4.37
4.35
4.32
4.30
2.14
1.34
1.31
1.29
NH
O
O
CH3
N
N
H3C
N+
O
O-
5j
0.97
1.99
1.99
2.98
2.00
2.94
3.06
15.0 14.5 14.0 13.5 13.0 12.5 12.0 11.5 11.0 10.5 10.0 9.5 9.0 8.5 8.0 7.5 7.0 6.5 6.0 5.5 5.0 4.5 4.0 3.5 3.0 2.5 2.0 1.5 1.0 0.5 0.0
f1 (мд)

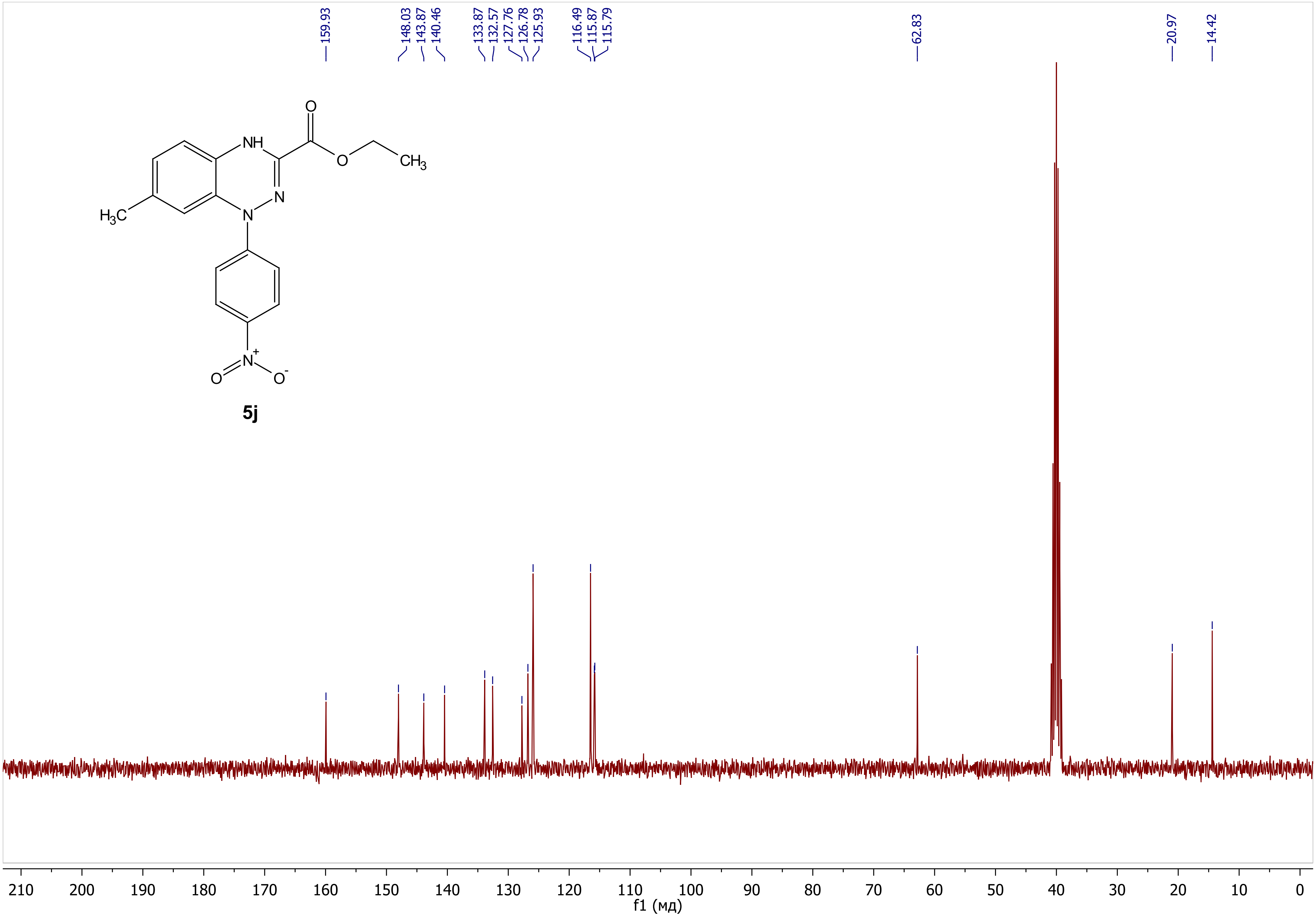

159.93
148.03
143.87
140.46
133.87
132.57
127.76
126.78
125.93
116.49
115.87
115.79
62.83
20.97
14.42
5j
210
200
190
180
170
160
150
140
130
120
110
100
90
80
70
60
50
40
30
20
10
0
f1 (мд)

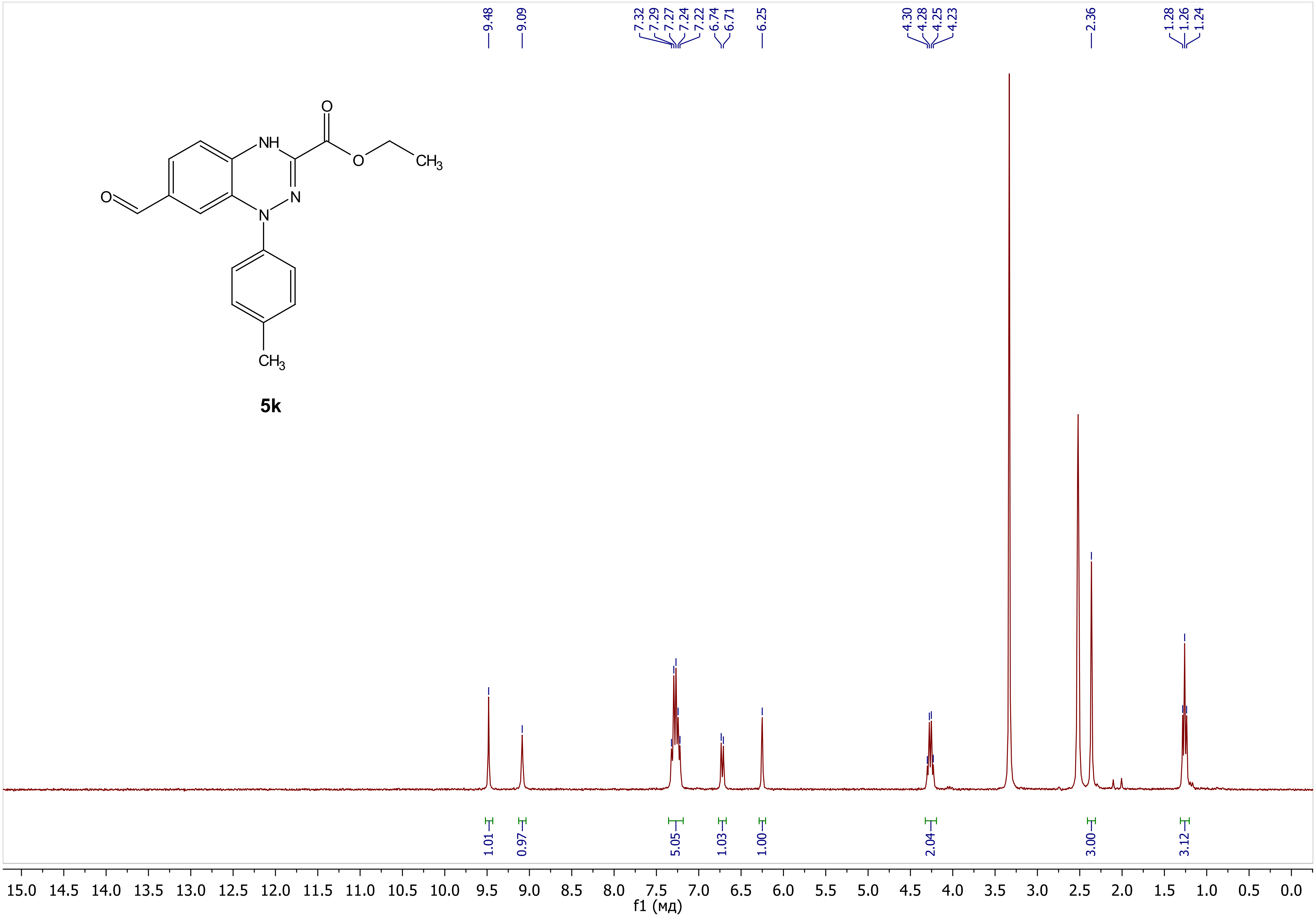

9.48
9.09
7.32
7.29
7.27
7.24
7.22
6.74
6.71
6.25
4.30
4.28
4.25
4.23
2.36
1.28
1.26
1.24
O
NH
O
CH3
O
N
N
CH3
5k
1.01
0.97
5.05
1.03
1.00
2.04
3.00
3.12
15.0 14.5 14.0 13.5 13.0 12.5 12.0 11.5 11.0 10.5 10.0 9.5 9.0 8.5 8.0 7.5 7.0 6.5 6.0 5.5 5.0 4.5 4.0 3.5 3.0 2.5 2.0 1.5 1.0 0.5 0.0
f1 (мд)

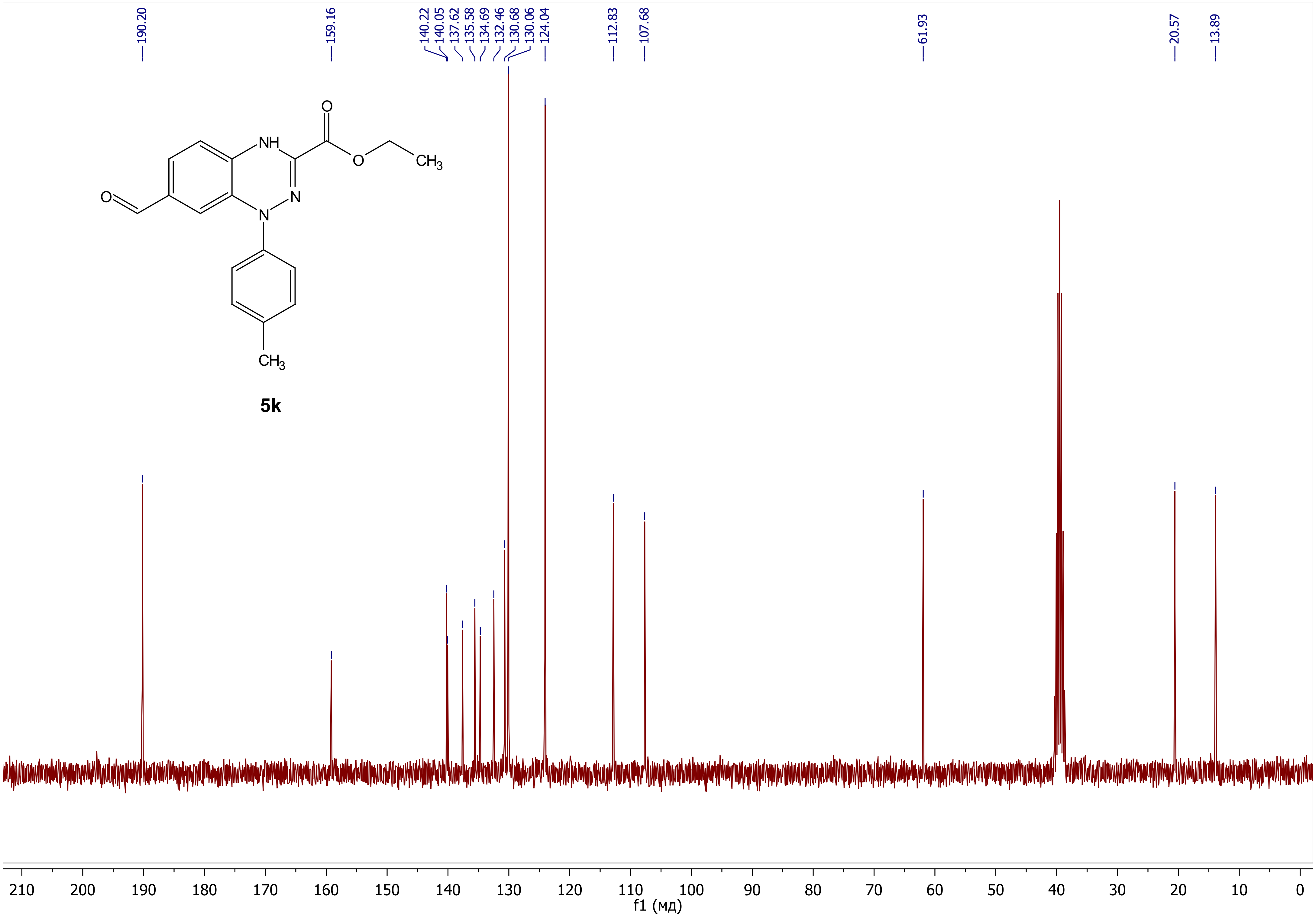
190.20
159.16
140.22
140.05
137.62
135.58
134.69
132.46
130.68
130.06
124.04
112.83
107.68
61.93
20.57
13.89
O
NH
O
CH3
O
N
N
CH3
5k
210 200 190 180 170 160 150 140 130 120 110 100 90 80 70 60 50 40 30 20 10 0
f1 (мд)

Display Report

Analysis Info

Analysis Name	D:\Data\Kolotyrkina\2021\Epishina\1005006.d	Acquisition Date	05.10.2021 11:06:32
Method	tune_50-1600.m	Operator	BDAL@DE
Sample Name	/PVYU 1073R	Instrument / Ser#	micrOTOF 10248
Comment	C15H10Cl2N3O mH 319.0273 calibrant added, CH3CN		

Acquisition Parameter

Source Type	ESI	Ion Polarity	Positive	Set Nebulizer	1.0 Bar
Focus	Not active			Set Dry Heater	200 °C
Scan Begin	50 m/z	Set Capillary	4500 V	Set Dry Gas	4.0 l/min
Scan End	1600 m/z	Set End Plate Offset	-500 V	Set Divert Valve	Waste

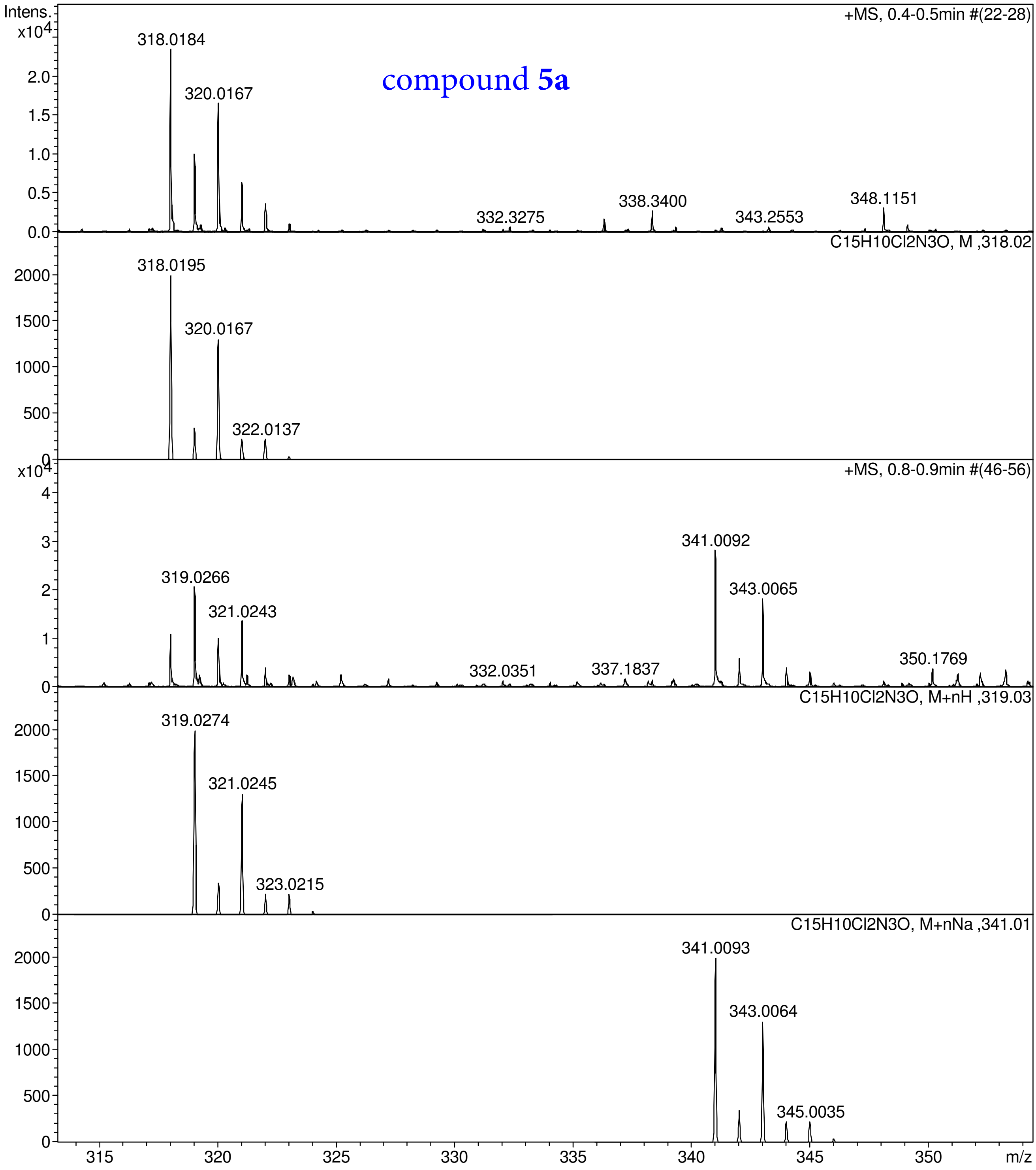

Display Report

Analysis Info

Analysis Name	D:\Data\Kolotyrkina\2021\Epishina\1109002.d	Acquisition Date	09.11.2021 9:51:51
Method	tune_50-1600.m	Operator	BDAL@DE
Sample Name	/PVYU 1082	Instrument / Ser#	micrOTOF 10248
Comment	C15H12N4O3 mH, 297.0982 calibrant added CH3CN		

Acquisition Parameter

Source Type	ESI	Ion Polarity	Positive	Set Nebulizer	1.0 Bar
Focus	Not active			Set Dry Heater	200 °C
Scan Begin	50 m/z	Set Capillary	4500 V	Set Dry Gas	4.0 l/min
Scan End	1600 m/z	Set End Plate Offset	-500 V	Set Divert Valve	Waste

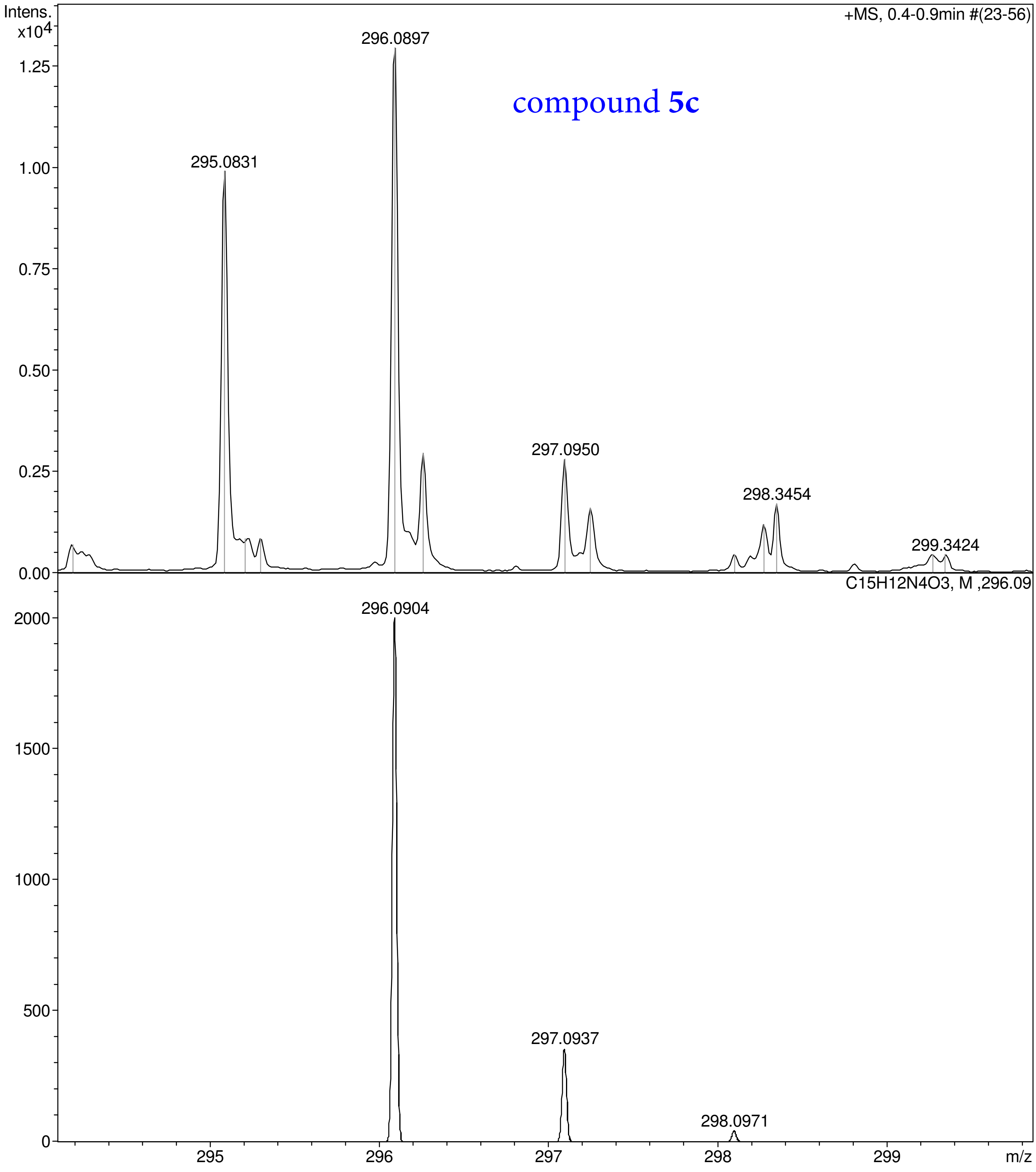

Display Report

Analysis Info

Analysis Name	D:\Data\Kolotyrkina\2022\Epishina\0118005.d	Acquisition Date	18.01.2022 12:05:40
Method	tune_50-1600_pos_15_12.m	Operator	BDAL@DE
Sample Name	/PVYU 1104-H_Rep	Instrument / Ser#	micrOTOF 10248
Comment	C18H18N3O2 m 309.1401 calibrant added CH3CN		

Acquisition Parameter

Source Type	ESI	Ion Polarity	Positive	Set Nebulizer	0.4 Bar
Focus	Not active			Set Dry Heater	180 °C
Scan Begin	50 m/z	Set Capillary	4500 V	Set Dry Gas	4.0 l/min
Scan End	1600 m/z	Set End Plate Offset	-500 V	Set Divert Valve	Waste

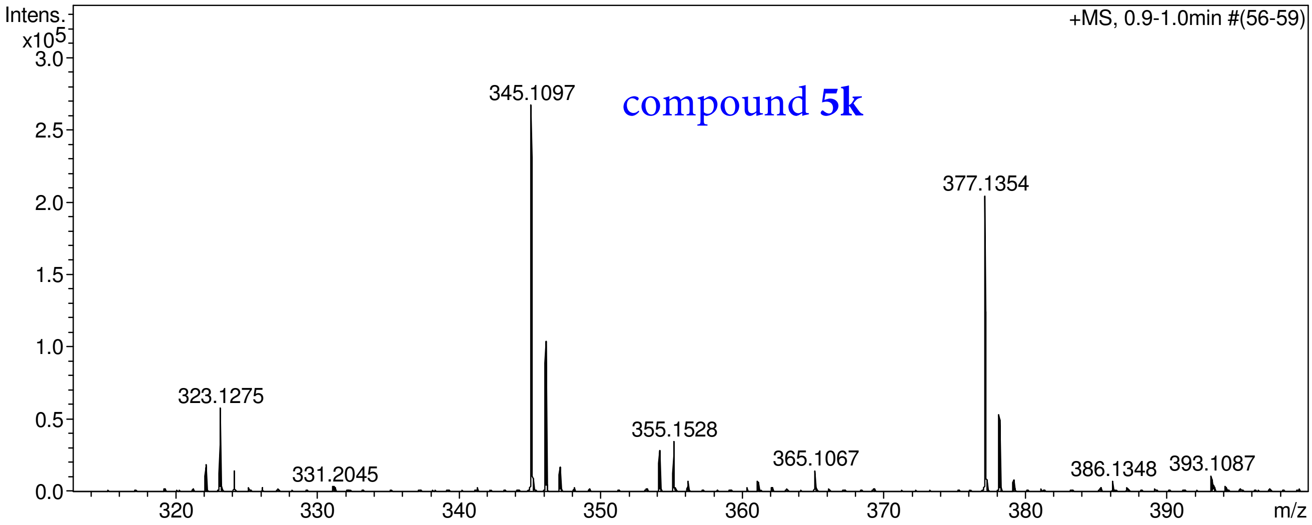

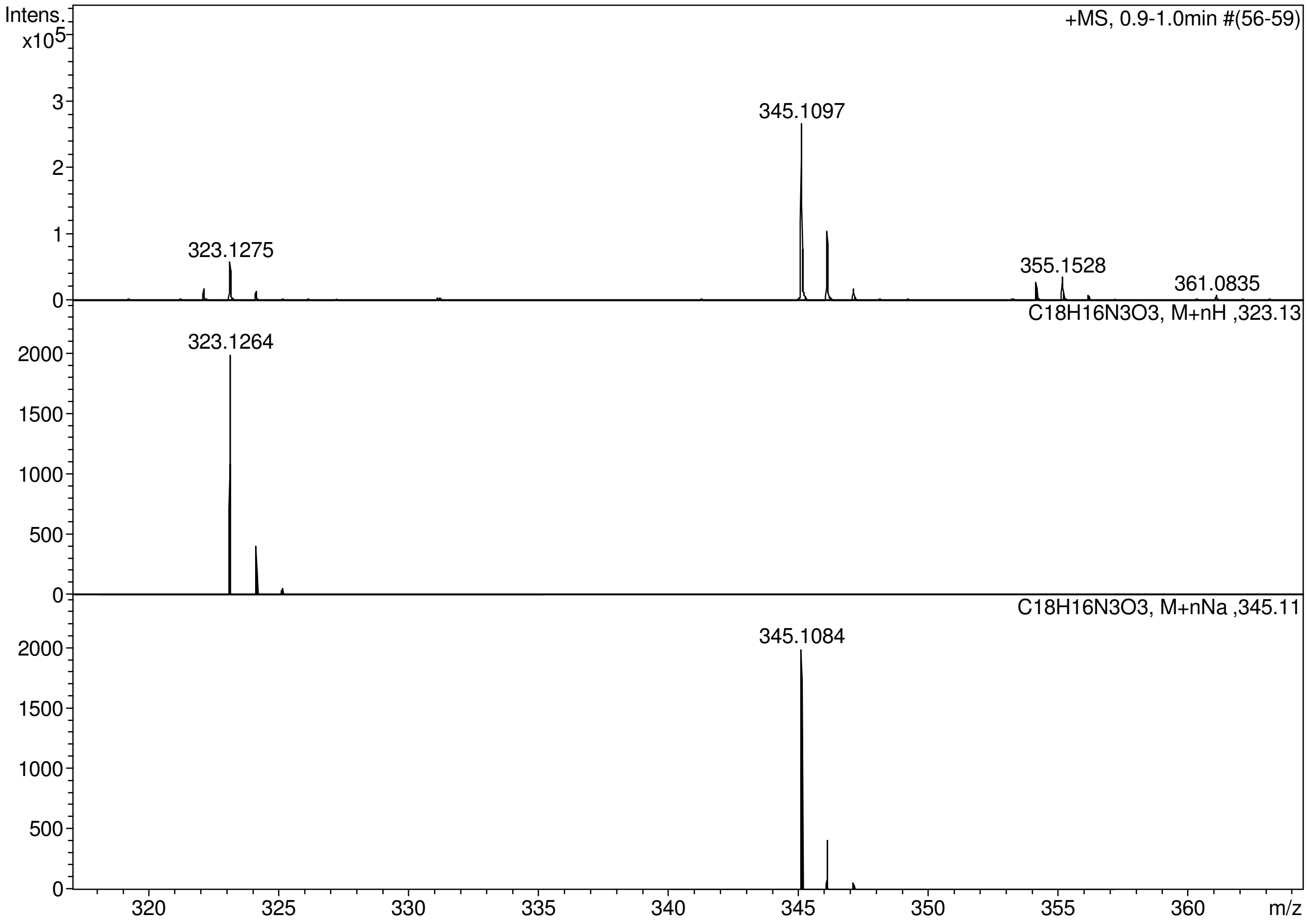

Display Report

Analysis Info

Analysis Name	D:\Data\Kolotyrkina\2021\Epishina\1209010.d	Acquisition Date	09.12.2021 11:05:38
Method	tune_50-1600.m	Operator	BDAL@DE
Sample Name	/PVYU 1099-K	Instrument / Ser#	micrOTOF 10248
Comment	C16H12Cl2N3O2 mH 349.0379 clb added CH3CN		

Acquisition Parameter

Source Type	ESI	Ion Polarity	Positive	Set Nebulizer	1.0 Bar
Focus	Not active			Set Dry Heater	200 °C
Scan Begin	50 m/z	Set Capillary	4500 V	Set Dry Gas	4.0 l/min
Scan End	1600 m/z	Set End Plate Offset	-500 V	Set Divert Valve	Waste

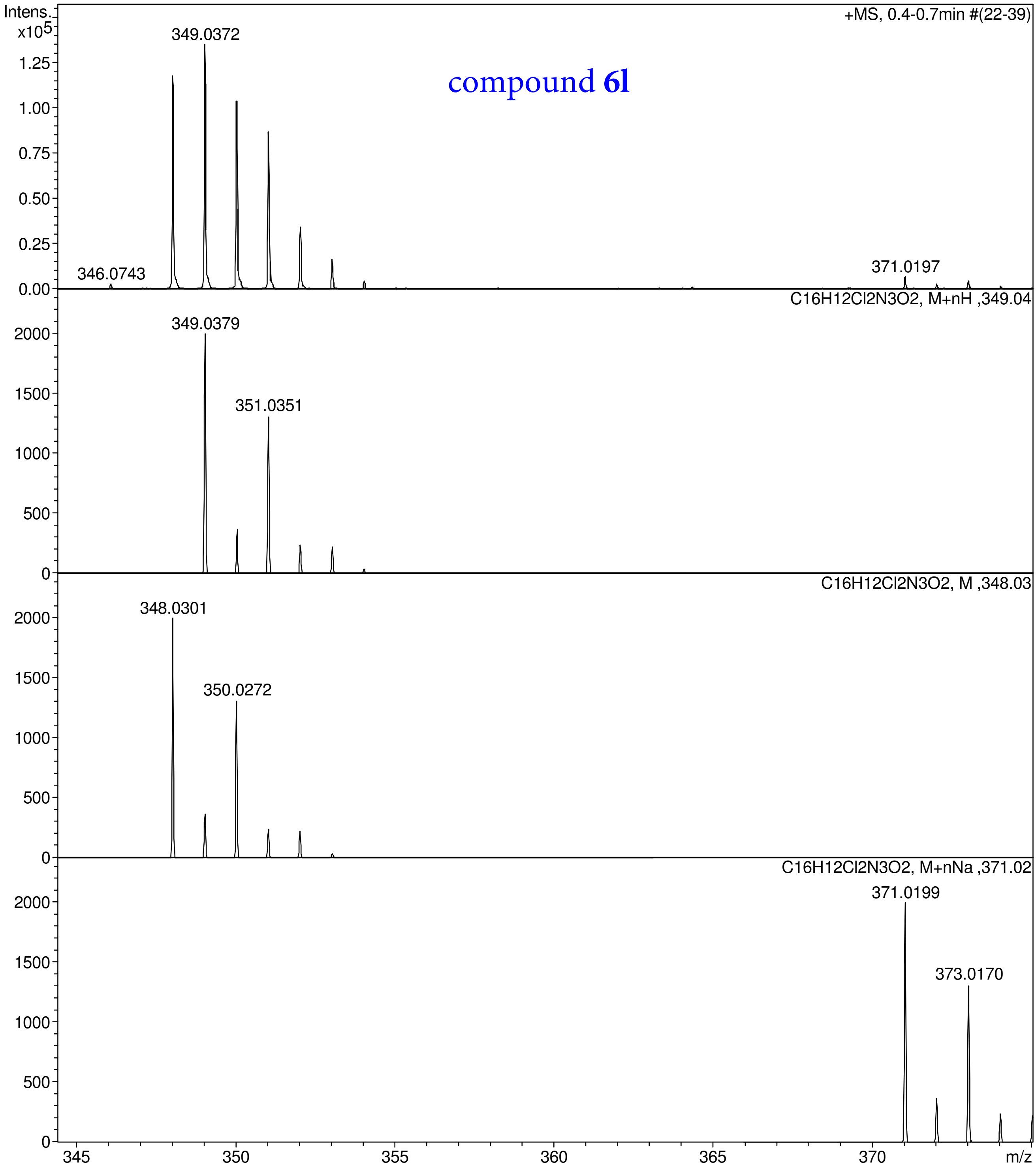

Display Report

Analysis Info

Analysis Name	D:\Data\Kolotyrkina\2021\Epishina\1214001.d	Acquisition Date	14.12.2021 10:18:56
Method	tune_50-1600.m	Operator	BDAL@DE
Sample Name	/PVYU 1099-C	Instrument / Ser#	micrOTOF 10248
Comment	C16H12Cl2N3O2 mH 335.0348, calibrant added		

Acquisition Parameter

Source Type	ESI	Ion Polarity	Positive	Set Nebulizer	1.0 Bar
Focus	Not active			Set Dry Heater	200 °C
Scan Begin	50 m/z	Set Capillary	4500 V	Set Dry Gas	4.0 l/min
Scan End	1600 m/z	Set End Plate Offset	-500 V	Set Divert Valve	Waste

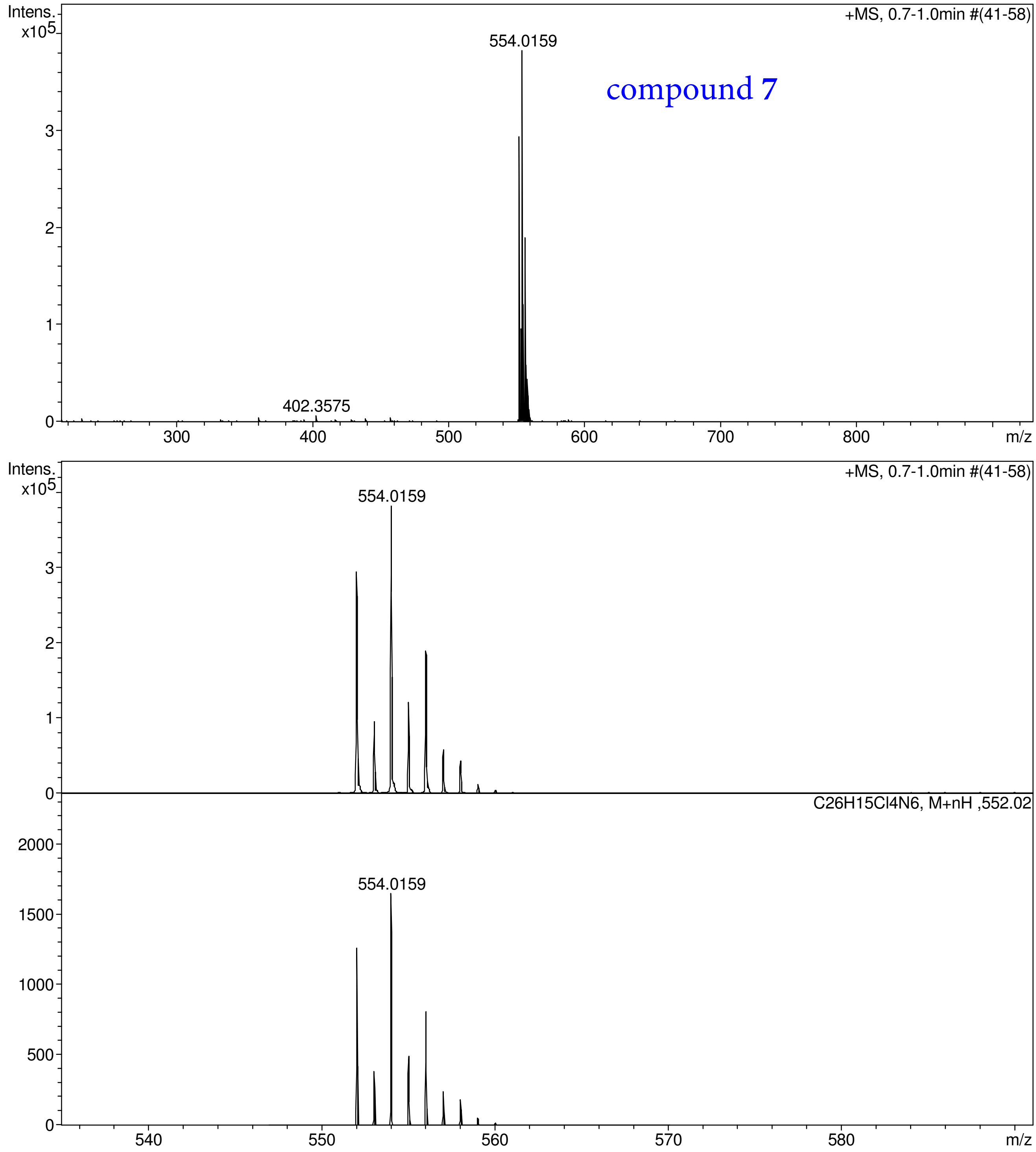

Display Report

Analysis Info

Analysis Name	D:\Data\Kolotyrkina\2021\Epishina\1214001.d	Acquisition Date	14.12.2021 10:18:56
Method	tune_50-1600.m	Operator	BDAL@DE
Sample Name	/PVYU 1099-C	Instrument / Ser#	micrOTOF 10248
Comment	C16H13Cl2N3O2 mH 334.0348, calibrant added		

Acquisition Parameter

Source Type	ESI	Ion Polarity	Positive	Set Nebulizer	1.0 Bar
Focus	Not active			Set Dry Heater	200 °C
Scan Begin	50 m/z	Set Capillary	4500 V	Set Dry Gas	4.0 l/min
Scan End	1600 m/z	Set End Plate Offset	-500 V	Set Divert Valve	Waste

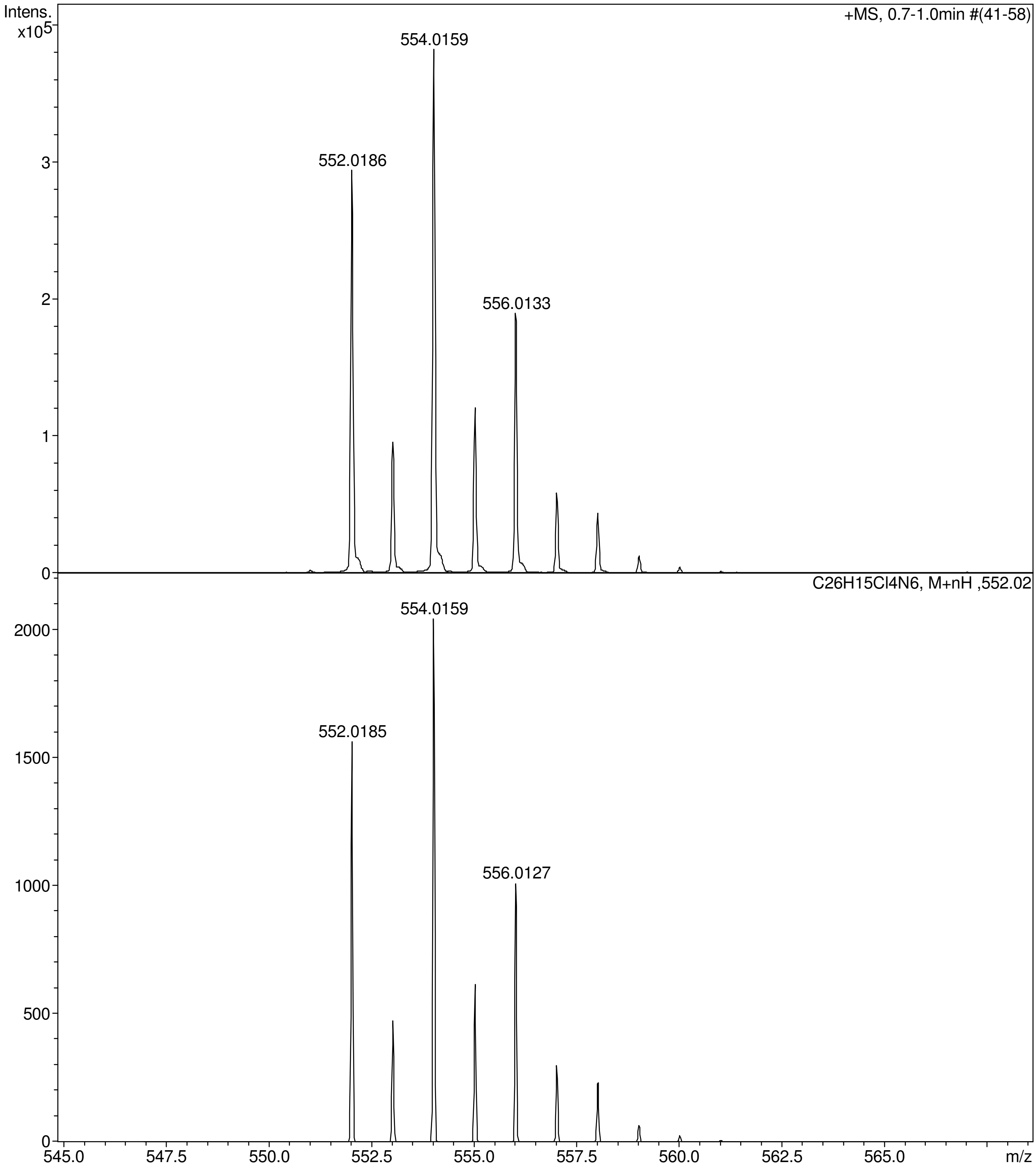

Supplement: Supplementary file 1 [file molecules-27-02575-s001.zip › molecules-1680249-supplementary.pdf]
